# Immobilization of functional nano-objects in living engineered bacterial biofilms for catalytic applications

**DOI:** 10.1093/nsr/nwz104

**Published:** 2019-07-30

**Authors:** Xinyu Wang, Jiahua Pu, Yi Liu, Fang Ba, Mengkui Cui, Ke Li, Yu Xie, Yan Nie, Qixi Mi, Tao Li, Lingli Liu, Manzhou Zhu, Chao Zhong

**Affiliations:** 1 Division of Materials and Physical Biology, School of Physical Science and Technology, ShanghaiTech University, Shanghai 201210, China; 2 Shanghai Institute of Ceramics, Chinese Academy of Sciences, Shanghai 200050, China; 3 University of Chinese Academy of Sciences, Beijing 100049, China; 4 School of Life Science and Technology, ShanghaiTech University, Shanghai 201210, China; 5 Shanghai Institute for Advanced Immunochemical Studies (SIAIS), ShanghaiTech University, Shanghai 201210, China; 6 College of Chemistry & Chemical Engineering, Anhui University, Hefei 230039, China

**Keywords:** nanoscale catalyst immobilization, semi-artificial photosynthesis, living catalysis, bio-inorganic hybrid system, hydrogen production

## Abstract

Nanoscale objects feature very large surface-area-to-volume ratios and are now understood as powerful tools for catalysis, but their nature as nanomaterials brings challenges including toxicity and nanomaterial pollution. Immobilization is considered a feasible strategy for addressing these limitations. Here, as a proof-of-concept for the immobilization of nanoscale catalysts in the extracellular matrix of bacterial biofilms, we genetically engineered amyloid monomers of the *Escherichia coli* curli nanofiber system that are secreted and can self-assemble and anchor nano-objects in a spatially precise manner. We demonstrated three scalable, tunable and reusable catalysis systems: biofilm-anchored gold nanoparticles to reduce nitro aromatic compounds such as the pollutant *p*-nitrophenol, biofilm-anchored hybrid Cd_0.9_Zn_0.1_S quantum dots and gold nanoparticles to degrade organic dyes and biofilm-anchored CdSeS@ZnS quantum dots in a semi-artificial photosynthesis system for hydrogen production. Our work demonstrates how the ability of biofilms to grow in scalable and complex spatial arrangements can be exploited for catalytic applications and clearly illustrates the design utility of segregating high-energy nano-objects from injury-prone cellular components by engineering anchoring points in an extracellular matrix.

## INTRODUCTION

Nanoscale objects, which feature very large surface-area-to-volume ratios, are now understood as uniquely powerful tools for designing catalysis-reaction systems [1–3]. Compared to conventional catalysts, nanoscale objects (e.g. nanoparticles and nanorods) frequently offer attractive enhancements in catalytic activity, selectivity and stability [4–7]. Despite their recognized potential and ongoing popularity among researchers, their nature as nanoscale objects brings along several attendant challenges that must be overcome before nanocatalysis approaches can become truly routine. For instance, the recovery of nanoscale objects typically requires energy-intensive ultra-centrifugation [8] and the *in situ* use of nanocatalysts in bio-remediation applications demands careful planning for recovery to prevent secondary pollution from the release of nano-objects [9]. It is also widely appreciated that the ability to reuse nanocatalysts over repeated reaction cycles is an important aspect of reaction design for both environmental and economic reasons [9–11].

The immobilization of nano-objects via a large number of technological approaches has been used to address these challenges, including inorganics (e.g. Au nanoclusters immobilized on SiO_2_ spheres [12]) and a variety of self-assembled biological substrates (NiO nanoparticles on DNA [13], Pt nanoparticles on peptide fibers [14], Au nanoparticles on protein fibers [15] and Ru nanoparticles on virus filaments [16]). However, immobilization methods that employ inorganic- or even biopolymer-based substrates lack the unique attributes of living systems like self-regeneration, cellular-growth-based scalability and functional flexibility through genetic engineering, not to mention the intrinsic advantage of synthesizing complex enzymes, functional molecules or other required reagents or components *in situ*. There have been some recent successes for the immobilization of nano-objects directly onto cellular membranes [17], for example the deposition of CdS nanoparticles on the outer membranes of *Escherichia coli* (*E. coli*) for hydrogen production [18] or on the surfaces of *Moorella thermoacetica* for acetic acid production [19]*.* Excitingly, the immobilization of such inorganic nano-objects with living substrates has been considered as an emerging catalysis strategy, potentially integrating the unique functionalities of synthetic nanomaterials with nature’s catalytic machinery, for future sustainable bioenergy [20] and bioremediation applications [21]. However, in light of the widely demonstrated use of high-energy nano-objects as biocidal agents [22,23], the reported short-term viability of cells with membrane-displayed nano-objects as well as the relatively loose interfaces formed between nanoparticles and cell membrane due to weak force interactions, it is clear that plenty of room still remains for creating more robust and benign abiotic/biotic interfaces for immobilization of the generally toxic inorganic nano-objects with living cells.

Biofilms are living multicellular communities tightly held together by a self-produced extracellular matrix [24]. They are widespread in nature, often exist as hierarchical ‘living coatings’ over various interfaces across multiple size scales [25] and have many extraordinary functional attributes including evolvability, environmental responsiveness, self-regeneration, excellent mechanical properties and ultra-stability in extreme and hostile environments [26–28]. Natural biofilms have been used in diverse functional applications such as water treatment [29], biofilm reactors [30], surface property modifiers [31], detoxification of toxic compounds [21] and electron transport [32]. Bacteria often use amyloid fibers—proteins that self-assemble to form various cross-beta nano-architectures—as scaffolds for biofilm formation [33]. Excitingly, recent foundational work in the engineering of bacterial biofilms has established that genetically engineered amyloid monomers with a variety of fused functional domains do not alter the monomers’ intrinsic ability to self-assemble into fibers and can endow bacterial biofilms with new functionalities [21,34–36].

**Figure 1:**
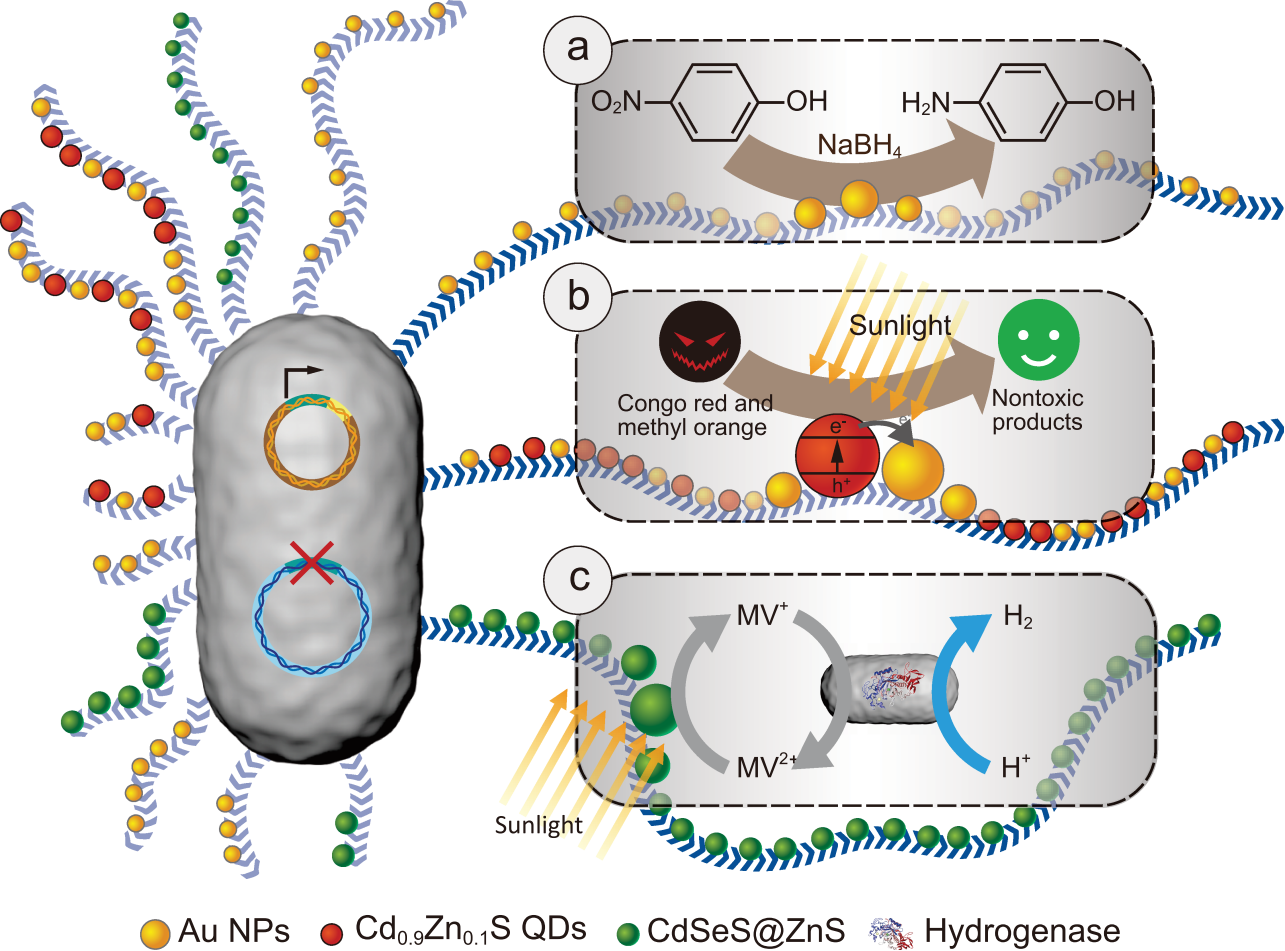
Diverse catalytic applications of tunable functional *E. coli* biofilms with anchored nano-objects. (a) The biofilm-anchored Au NPs enable the recyclable catalytic reduction of the toxic *p*-nitrophenol (PNP) into the harmless *p*-aminophenol (PAP). (b) The biofilm-anchored heterogeneous nanostructures (Au NPs/Cd_0.9_Zn_0.1_S QDs) photocatalyse the degradation of organic dyes to low-toxic products based on facile light-induced charge separation. (c) The biofilm-anchored quantum dots coupled with the engineered strain enable photo-induced hydrogen production. Electrons are transferred from QDs to hydrogenase using methyl viologen (MV) as a mediator.

Here, harnessing the engineerable feature of bacterial biofilms, we propose an amyloid matrix-anchoring concept to immobilize a variety of inorganic nano-objects, thereby addressing the aforementioned needs to robustly interface and immobilize nanoscale functional objects with living cells. Specifically, we engineered amyloid monomers to contain His-tags (CsgA_His_) and expressed the proteins in *E. coli* biofilms and simultaneously used ‘NTA-Metal-His’ coordination chemistry (Supplementary Fig. 1) and electrostatic interaction to anchor nano-objects to the biofilms [25]. To showcase the biofilm-anchored nano-objects for broad and diverse catalysis applications, we chose three reaction systems closely relevant to applications in the fields of environment and energy. We designed and successfully implemented three simple, scalable, tunable and reusable hybrid-reaction systems based on this amyloid biofilm matrix-anchoring concept: a living biofilm system to reduce the pollutant *p*-nitrophenol (PNP) based on the anchoring gold nanoparticles (Au NPs) (Fig. 1a), a living biofilm system to degrade organic dyes like Congo red (CR) based on anchoring Cd_0.9_Zn_0.1_S quantum dots (QDs) to biofilms (Fig. 1b) and a semi-artificial photosynthesis system based on living biofilms with anchored CdSeS@ZnS QDs and another engineered strain harboring multi-meric enzymes to achieve hydrogen production (Fig. 1c). The ability to use materials composed of living cells as integral parts of catalysis-reaction systems offers the ability to incorporate ‘biology-only’ attributes like self-regeneration [37], environmental responsiveness [38,39] and genetically tunable functionality [21]. Thus, our successful proof-of-concept demonstrations push the frontier forward for coupling the uniquely dynamic properties and capacities of living materials with functional nano-objects to achieve ever more capable, efficient and sustainable catalysis systems to solve challenges in bioremediation, bioconversion and energy. 

## RESULTS

### A biofilm-anchored Au NP living catalysis system for the reduction of the pollutant PNP

PNP, widely used in the production of pesticides and dyes, is a major organic pollutant in wastewater, and it is well established that Au nanoparticles (NPs) can catalyse the reduction of nitro aromatic compounds to corresponding amines in the presence of NaBH_4_ [15,40]. However, with the Au NP-catalysed reactions, two limitations have been extensively reported: (i) the requirement for complicated multi-step processes to anchor catalyst particles to control their location and (ii) the difficulties for recycling these catalyst particles [12,41–46]. We here anchored Au NPs to engineered *E. coli* biofilms with integrated His-tags via a one-step method that synchronized biofilm formation and Au NP assembly using either ‘NTA-Metal-His’ coordination chemistry or electrostatic interactions.

**Figure 2:**
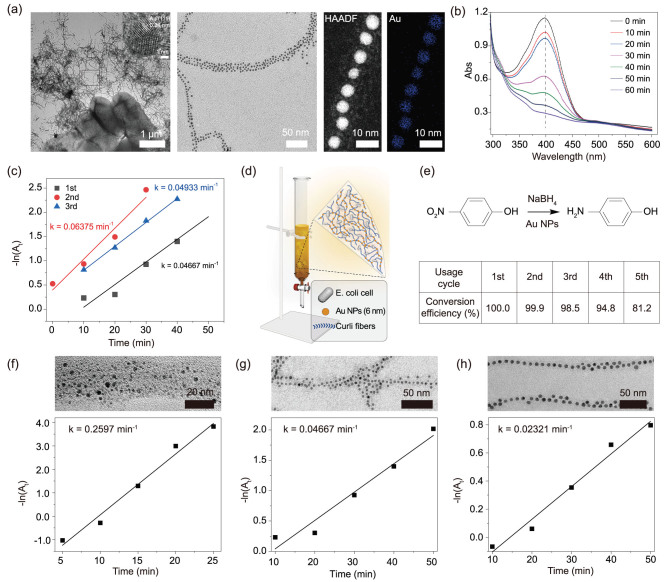
Recyclable reduction of *p*-nitrophenol with *E. coli* biofilm-anchored Au NPs as catalysts. (a) TEM, HRTEM, HAADF STEM imaging and EDS mapping of *E. coli* biofilm-anchored Au NPs (5.2 nm). (b) UV-Vis spectra monitoring of the reduction of *p*-nitrophenol catalysed by polystyrene-substrate-attached *E. coli* biofilm-anchored Au NPs (5.2 nm). (c) Catalytic activity comparison of three consecutive reuse instances of *E. coli* biofilm-anchored Au NPs (5.2 nm) on the bottom of a 12-well plate. (d) Illustration of a simple device for recyclable catalysis as enabled by functional PP-thin-flake-substrate-attached biofilm-anchored Au NPs (5.2 nm). (e) Conversion-efficiency comparison of five consecutive reuse instances of the device shown in (d). The conversion efficiencies are 100, 99.9, 98.5, 94.8 and 81.2% for five cycles of a 16-min reaction. (f)–(h) The catalytic efficiencies of *p*-nitrophenol reduction with *E. coli* biofilm-anchored Au NPs with a diameter of 2.1 ± 0.5 nm (f), 5.2 ± 0.5 nm (g) and 7.9 ± 0.6 nm (h). The TEM images in (f)–(h) were the enlarged images in the rectangular box in Supplementary Fig. 6. Note: ‘Au NPs’ here refer to water-soluble Au NPs.

Upon tetracycline induction, *E. coli* Tc_Receiver_/CsgA_His_ seed cells growing in M63 culture media can form biofilms on a given substrate after 2 days (Supplementary Figs 4 and 5). To prepare biofilm-anchored Au NPs, Tc_Receiver_/CsgA_His_ seed cells were purposely grown in 12-well plates containing low-nutrient M63 culture media, supplemented with Au NPs that were synthesized according to reported research [46,47]. Transmission electron microscopy (TEM), high-resolution transmission electron microscopy (HRTEM), high-angle annular dark-field scanning transmission electron microscopy **(**HAADF STEM) and energy-dispersive X-ray spectroscopy (EDS) mapping were used for morphological analysis and elemental mapping of the *E. coli* biofilm-anchored Au NPs (5.2 nm) in the hybrid system (Fig. 2a). The biofilms were richly coated with anchored Au NPs of a specific diameter (2.1, 5.2 or 7.9 nm), dependent on the type of Au NPs that were initially supplemented to the culture media (Supplementary Fig. 6a–c and Fig. 2f–h). Micrographs showed that Au NPs were neatly ordered along the curli fibrils of the biofilm and these anchored NPs had the same diameters as the originally synthesized free Au NPs. EDS mapping confirmed that the NPs were indeed gold (Fig. 2a).

The PNP reduction was carried out in the aforementioned 12-well plates using *in vivo* reaction systems with a NaBH_4_-to-PNP molar ratio of 300:1, which guaranteed first-order kinetics for the reduction of PNP to *p*-aminophenol (PAP) and would not impair the stability of our living systems as demonstrated later on. The reaction proceeded to completion in 1 h. Importantly, the Au NPs remained anchored to the biofilm following the reaction (Supplementary Fig. 7), implying that the ‘NTA-Metal-His’ coordination chemistry or electrostatic interactions were robust even after exposure to reaction solutions. The robustness of this operation system also enabled testing over multiple cycles. Monitoring catalytic performance with UV-Vis spectroscopy showed that these systems could efficiently convert PNP to the reduction product PAP, as indicated by the appearance of the sharp peak at 300 nm in the spectra (Fig. 2b and Supplementary Fig. 8). Since smaller Au NPs have larger catalytic surface areas, the catalytic rate constants for the *in vivo* hybrid-reaction systems with the 2.1, 5.2 and 7.9 nm Au NPs were 0.2597, 0.04667 and 0.02321 min^−1^, respectively (Fig. 2f–h and Supplementary Fig. 6a–c), showing an increasing trend as anticipated. We applied the intermediate size of Au NPs (5.2 nm) as the representative of Au NPs for subsequent demonstrations, as the preparation of 5.2 nm Au NPs is less time-consuming (compared with 2.1 nm) and the size practically facilitates the operation and evaluation of the recycling property of our living catalytic reaction system.

Using a standard addition method, we determined that the loading capacity of biofilms for 5.2 nm Au NPs was around 30.4 μg/mg (Au NPs/biofilms) (Supplementary Fig. 9a). Using the determined loading capacity of Au NPs of biofilms as a reference, we applied biofilm-anchored Au NPs samples with a variably loaded amount of Au NPs for PNP reduction; the rate constant increased with the increase in the amount of Au NPs anchored on the biofilms (Supplementary Fig. 9b). The reduction reaction only occurred at the bottom interface of the 12-well plate in our current reaction system as a result of the formation of biofilms at the bottom of the substrate. Thus, it is not surprising to notice that the rate constant (0.04667 min^−1^) based on this heterogeneous reaction system (Fig. 2g) was lower than free Au NPs in reaction solution (0.5904 min^−1^) (Supplementary Fig. 10). Intriguingly, when we scraped the biofilm-anchored Au NPs off the substrate and mixed the sample with the reaction solution, the rate constant for the freely suspended system increased to 0.5529 min^−1^ (Supplementary Fig. 10), which was comparable to that for the reaction system containing free Au NPs.

We next assessed the recycling property of our living catalytic reaction system. Similar PNP-reduction kinetics were maintained over three cycles of catalysis in these 12-well plates (Fig. 2c). Notably, bacterial biofilms remained alive even after the reaction processes, as revealed by the successful bacterial regrowth on agar plate based on a cell-regeneration assay (Supplementary Fig. 26a). Larger-scale experiments in which we grew biofilms and anchored 5.2 nm Au NPs on flat polypropylene (PP) blades (Fig. 2d and Supplementary Fig. 11) later revealed that the *in vivo* hybrid-reaction system retained 81% of its original reduction efficiency after five reaction cycles (Fig. 2e). Thus, our PNP-reduction systems (in both well-plate and PP-blade formats) featuring engineered biofilm-anchored nano-objects demonstrate proof-of-concept for reusable living biofilm matrix catalysis systems. Note that *E. coli* biofilms are easily grown in nutrient-deficient media and can be grown in a huge variety of 2D and 3D contexts [25]. Thus, our biofilm catalysis systems should in theory be expandable for large-scale practical industrial applications.

### Biofilm-anchored heterogeneous nano-objects (Au NPs and Cd_0.9_Zn_0.1_S QDs) for the photocatalytic degradation of organic dyes

Chalcogenide semiconductors with a wide band gap, such as Cd_1–x_Zn_x_S QDs, have been demonstated as highly active photocatalysts for the degradation of a variety of organic dyes that are often environmentally detrimental [48,49]. Nanosized semiconductors exhibit higher photocatalytic effeciencies compared with macroscale materials of the same type and are thus viewed as highly promising for catalysis applications [50], yet difficulties in immobilizing nanosized materials and attendant secondary pollution are commonly recognized problems [9]. We here aimed to accomplish the photocatalytic degradation of organic dyes such as CR using *in vivo* biofilm/Cd_0.9_Zn_0.1_S QDs/Au NPs hybrid-reaction systems.

**Figure 3:**
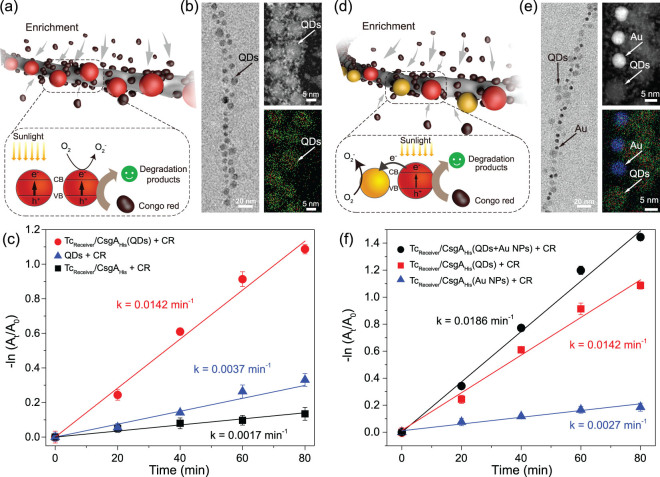
Accelerated photodegradation of Congo red using *E. coli* biofilm-anchored hybrid structures as catalysts. **(**a) Schematic for the degradation of Congo red using *E. coli* biofilm-anchored QDs as catalysts. (b) TEM (left), HAADF STEM (top right) and EDS mapping (bottom right) results of *E. coli* biofilm-anchored Cd_0.9_Zn_0.1_S QDs. (c) Photodegradation of CR with free QDs, Tc_Reciever_/CsgA_His_ biofilms or Tc_Reciever_/CsgA_His_ biofilm-anchored QDs as catalysts. (d) Schematic of CR degradation using *E. coli* biofilm-anchored Cd_0.9_Zn_0.1_S QDs and Au NPs as catalysts. (e) TEM (left), HAADF STEM (top right) and EDS-mapping (bottom right) results of *E. coli* biofilm-anchored Cd_0.9_Zn_0.1_S QDs and Au NPs. (f) Photodegradation of CR with Tc_Reciever_/CsgA_His_ biofilm-anchored Au NPs, Tc_Reciever_/CsgA_His_ biofilm-anchored QDs or Tc_Reciever_/CsgA_His_ biofilm-anchored QDs and Au NPs as catalysts. Notes: In the schematics of (a) and (d), the red spheres represent QDs while the yellow spheres represent Au NPs. The dark-brown particles represent Congo red molecules. O_2_ is the major sacrificial agent for accumulated photo-generated electrons. For the EDS-mapping results in (b) and (e), the green color represents Zn, the blue color represents Au and the red color represents Cd.

We especially chose Cd_0.9_Zn_0.1_S QDs because Cd_0.9_Zn_0.1_S, compared with other Cd_1–x_Zn_x_S QDs, had the absorption in the visible region that met the need for the photodegradation of organic dyes under visible light in our designed experiment (Supplementary Fig. 12). Specifically, Cd_0.9_Zn_0.1_S QDs—which were anchored in the engineered biofilm via His-tags—have a known ability to perform a light-induced, charge-separation-driven degradation reaction of CR into a variety of complex but low-toxic-breakdown products such as phenol and carboxylic acid derivatives [49,51], and the accumulated electrons generated from the reaction could be consumed by O_2_ under ambient conditions [52]. Briefly, the *in vivo* biofilm/Cd_0.9_Zn_0.1_S QDs/Au NPs hybrid-reaction system features two notable design elements. First, as the hydrophobic pockets present in amyloid biofilms are known to adsorb organic dyes [53], the affinity of our amyloid biofilms for the hydrophobic regions of organic molecules should enrich the local concentration of the dyes being targeted for degradation [39] (Fig. 3a). Second, the co-anchoring of Au NPs into the biofilm matrix via His-tags should exploit the reported ability of Au NPs to accept electrons generated by QDs to promote charge separation and thereby accelerate the catalytic rate of the CR-degradation reaction [52,54] (Fig. 3d).

TEM, HAADF STEM and EDS mapping were used to characterize the organization of the QDs and Au NPs along curli fibers in various configurations of the hybrid system, including QDs alone (Fig. 3b and Supplementary Fig. 14a) and co-anchored QDs and Au NPs (Fig. 3e and Supplementary Fig. 14b). QDs and Au NPs were copiously anchored to curli fibers and, for the system with both QDs and Au NPs, the two types of nanoparticles were distributed in close proximitty but in an apparently random manner. Further, EDS mapping revealed nanoparticles with the appropriate compositions of cadmium, zinc, sulphur and gold (Fig. 3e). Upon exposure to high-energy light from a Xe lamp, the degradation of CR was analysed via UV-VIS spectroscopy by monitoring the absorption at 496 nm and the sample curves were fitted using pseudo-first-order approximation [55] to facilitate quantitative comparisons.

We initially conducted control experiments comprising CR and free Cd_0.9_Zn_0.1_S QDs. There was only weak degradation of CR, with a rate constant of 3.7 × 10^−3^ min^−1^, indicating that free QDs do not effciently degrade CR; nor was there any obvious CR degradation in experiments with only the biofilm (1.7 × 10^−3^ min^−1^) (Fig. 3c and Supplementary Fig. 15). The hybrid biofilm-QD system lacking Au NPs dispersed along curli fibers and exhibited a degradation-rate constant of 14.2 ×10^−3^ min^−1^—three times higher than the rate for free QDs in solution (Fig. 3c and Supplementary Fig. 15). We speculate that this observed improvement in the effciency of the catalytic-degradation results from the aforementioned ability of hydrophobic pockets in amyloid biofilms to enrich the local concentration of CR near the anchored QDs in the biofilm matrix. Notably, the biofilm-anchored hybrid structures exhibited impressive robustness for long-hour and multiple rounds of dye degradation, as revealed by the maintained high level of the catalytic rate even after three continuous cycles of photodegradation (Supplementary Fig. 16a) and the intact hybrid structural features under TEM (Supplementary Fig. 16b).

Before testing the co-anchored heterogeneous nano-objects system, we also tested a control system in which only Au NPs were anchored in the biofilm matrix: only negligible degradation of CR (2.7 × 10^−3^ min^−1^) was observed. Excitingly, we detected a 31% increase in the rate of CR degradation for the biofilms anchored with both QDs and Au NPs (18.6 × 10^−3^ min^−1^), compared with biofilms anchored with only QDs (14.2 × 10^−3^ min^−1^) (Fig. 3f and Supplementary Fig. 15). Furthermore, we also found that the increase in Au-to-QDs volume ratios in the culture medium led to larger amount of Au NPs anchored onto the biofilms and thereby a higher degradation rate for CR photodegradation (Supplementary Fig. 17). We ascribe this increase to an Au NP-mediated increase in the charge separation that accelarates the QD-mediated degadation reactions. Indeed, the stead-state and time-resolved fluorescent spectra clearly revealed that substantially depressed fluorescent emission and faster fluorescence decay kinetics occurred for the biofilms anchored with heterogeneous structures compared to biofilms anchored with only QDs (Supplementary Fig. 18). Intriguingly, the bacterial-regeneration assay again showed that biofilms were alive after the reaction process and the solution seed could regrow into new colonies on the agar plate (Supplementary Fig. 26b). We also conducted experiments with the organic dye methyl orange (MO) and, beyond finding that biofilm-QD systems could degrade this organic dye efficiently, we noted a similar trend in the efficiency increase when Au NPs and QDs were co-anchored to the curli fibers of the biofilm (Supplementary Figs 19 and 20). Thus, the heterogeneous anchoring of QDs and Au NPs into biofilms to increase the efficiency of organic-dye-degradation reactions demonstrates proof-of-concept for living catalysis systems using more than one type of anchored nano-object.

### Biofilm-anchored QDs coupled with an engineered strain for photo-induced hydrogen production

Bio-inorganic hybrid systems have attracted a lot of interest for converting solar energy to chemical energy [56] and several strategies for creating such systems have employed photo-sensitive semiconductors and hydrogenase enzymes that catalyse the reversible reaction of protons to hydrogen [57]. Typically, purified hydrogenase enzymes are used with inorganic semiconductors, but the low O_2_ tolerance and high expense of generating matured and functional recombinant hydrogenase enzymes have hindered their practical usage [58]. Further, the photo-sensitive semiconductors used in these systems (e.g. CdTe nanoparticles, CdS nanorods) are difficult to separate and reuse [59,60]. Living engineered inorganic-bacteria hybrid structures have also been used for hydrogen or acetic acid production, although the direct contact of semiconductors with the bacteria surface may cause serious damage to the bacterial cells [19].

**Figure 4:**
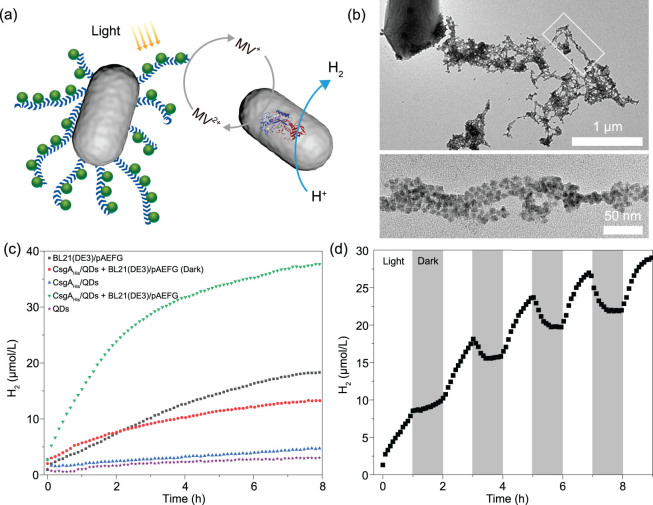
Biofilm-anchored quantum dots coupled with the engineered strain for photo-induced hydrogen production. (a) Schematic for the hydrogen-production process in our *in vivo* hybrid semi-artificial photosynthesis system. (b) TEM images of Tc_Receiver_/CsgA_His_ biofilm-anchored QDs. The bottom image is the zoomed-in image of the white rectangle in the top image. (c) Hydrogen production over time catalysed under different experimental conditions. (d) Hydrogen production under light–dark cycles. Notes: TEOA represents triethanolamine as a sacrificial agent. MV represents methyl viologen as an electron mediator. HydA represents [FeFe] hydrogenase. BL21(DE3)/pAEFG means *E. coli* cells containing hydrogenase. CsgA_His_/QDs refers to Tc_Receiver_/CsgA_His_ biofilms with anchored QDs. QDs used here was CdSeS@ZnS QDs. The light source used here was an artificial blue-light source with a current of 0.3 A.

We here designed a semi-artificial photosynthesis system comprising biofilm-anchored photosensitizer CdSeS@ZnS QDs as well as a second bacterial strain expressing a hydrogenase and its maturases (Fig. 4a and Supplementary Fig. 21). In our sytem, methyl viologen (MV) was purposely added as an electron mediator to efficiently transfer the photo-generated electrons to the introcellularly expressed hydrogenase. We first synthesized the photosensitizer CdSeS@ZnS QDs based on an estabilished method [25]. Engineered biofilms with extensive extracellular CsgA-His curli fibers were produced by growing the Tc_Receiver_/CsgA_His_ strain in a Petri dish for 2 days in growth media supplemented with the synthezied photosensitizer CdSeS@ZnS QDs. TEM analysis of harvested biofilms confirmed that the QDs were anchored to the curli fibers in the biofilm matrix (Fig. 4b). To generate the hydrogenase for our system to cataylyse H_2_ production, we leveraged the ACEMBL expression system for multigene expression [61] to make a single-fusion plasmid containing four genes from *Clostridium pasteurianum* encoding [FeFe] hydrogenase: HydA and the maturases HydE, HydF and HydG [62]. This multigene plasmid was expressed in BL21(DE3) cells to generate the hydrogenase-producing bacteria referred to as *E. coli* BL21(DE3)/pAEFG (Supplementary Figs 22, 23, 25 and 27–30).

For testing the photocatalytic H_2_ production using our *in vivo* two-strain system, we harvested the Tc_Receiver_/CsgA_His_ biofilm-acchored QDs and added the harvested sample to a buffered solution in a vial (PBS solution containing 5.0% glycerol, pH = 8.0) that also contained the BL21(DE3)/pAEFG strain (~100 mg wet weight) and a microsensor to detect the hydrogen production. The system also included triethanolamine (TEOA) (1.5%) as a sacrificial electron donor and MV (5 mM) as an electron mediator to shuttle electrons to the hydrogenase. When the complete two-strain system was exposed to light (an artificial blue-light source with a current of 0.3 A), it produced H_2_ and continuous illumination for 8 h led to a final H_2_ concentration of 37.7 μmol/L (Fig. 4c). The reaction activity obviously declined after 3 h, likely arising from the biodirectional activities of the hydrogenase that can catalyse either the proton reduction to hydrogen gas or hydrogen oxidation. Apparently, as the concentration of H_2_ in the solution increased, the production rate of H_2_ would decrease as a result of the rate increase of the reversible reaction.

In comparison, only a negligible amount of H_2_ production was observed in control experiments lacking the BL21(DE3)/pAEFG strain or in the reaction system containing only QDs. However, we observed low but appreciable levels of H_2_ production (~13 μmol/L) under darkness. In addition, H_2_ production was also detected in a system containing the BL21(DE3)/pAEFG strain but lacking the biofilm-anchored QDs (~18 μmol/L), perhaps owing to some endogenous metabolic actvity of the engineered bacterial strain. We also tested light–dark intervals and found that the H_2_ concentration generally increased during periods of illumination but decreased in the darkness. For the initial darkness period, when the H_2_ concentration was around 10 μmol/L, we did not observe a concentration decrease, but rather a slight increase. The final H_2_ concentration tested after 9 h (during the fifth light intervals) reached 29.2 μmol/L (Fig. 4d). Notably, QDs remained anchored to the biofilm following the reaction, and both the biofilms and the engineered strain harboring hydrogenases remained alive even after continous hydrogen production for 12 h (Supplementary Fig. 26c). Our results thus showcase a living photocatalytic catalyst system for H_2_ production that incorportates and synergizes two engineered strains and illustrates how two strains can be combined to share a division of labor by, on the one hand, anchoring the photorecptor nano-objects in the extracellular matrix of a biofilm for photo-reception and, on the other, using a whole-cell biocatalyst for the multi-enzymatic production of H_2_ from protons. Future work will likely engineer a single strain that integrates these two roles.

## CONCLUSION AND DISCUSSION

We here demonstrated three simple, robust and reusable catalysis systems based on anchoring nano-objects to living biofilms. Specifically, we designed and successfully implemented three *in vivo* hybrid-reaction systems based on the amyloid matrix-anchoring concept: a scalable system to reduce the pollutant PNP based on anchoring gold nanoparticles (Au NPs) to a biofilm; a photocatalytic system to degrade organic dyes like CR based on co-anchoring Cd_0.9_Zn_0.1_S QDs and Au NPs to a biofilm that employs an intriguing charge-separation mechanism to increase its efficiency; and a semi-artificial photosynthesis system based on biofilm-anchored CdSeS@ZnS QDs alongside a second engineered bacteria strain to successfully photocatalyse hydrogen production.

On the one hand, our choice to use living biofilms as scaffolds for the anchoring of nano-objects represents an advance in the area of inorganic nano-object immobilization by offering capacities not available with non-living systems, including the ease of scalability (both in 2D and 3D), self-regeneration and the ability of cells to biosynthesize, modify and secrete complex enzymes, substrates, co-enzymes, other required reagents or reaction components *in situ*, not to mention their evolvability that can be exploited to improve performance using directed evolution approaches. Additionally, we found that, after several rounds of catalysis, the nano-objects remained anchored in the biofilm matrix and retained activity. This stability suggests minimal leaching of nano-objects for environmentally sensitive applications.

On the other hand, our choice to engineer scaffold proteins that are secreted and reside outside cells represents a conceptual advance that establishes the design option of segregating high-energy nano-objects from injury-prone cellular sctructures. Fundamentally, our work demonstrates a new approach for conducting high-energy nano-object-mediated catalytic reactions in living systems that lessens the damage that should be expected to occur when NPs are placed in extremely close proximity to biological membranes. Indeed, many nano-objects are understood as biocidal agents that kill cells by disrupting membrane integrity [63,64] and previous work with CdS nanoparticles positioned on a bacterial surface reported that high light conditions resulted in the formation of large holes that led to the complete destruction of the cell membrane [19]. Also related to the extracellular location of the nano-objects in our systems, we demonstrated an increase in the degradation efficiency of CR by exploiting the ability of the hydrophobic pockets in extracellular amyloid fibers to increase the highly localized concentration of CR in close proximity to QDs. These examples of how the fine-tuning of spatial arrangements can impact the catalytic performance of our living biofilm-based systems underscore the very large scope of potential modifications to these systems to further improve performance.

A very large number of bacterial species are known to produce biofilms, which can feature some very substantial differences in their material properties, including, for example, *Geobacter* species that produce biofilms that are electrically conductive. Notably, at the nexus of ecology and engineering, microbial biofilms on their own have recently been considered as living biocatalysts for performing challenging conversions in controlled environments [30]. Beyond experimenting with various biofilm-producing species, it is easy to envision the development of catalytic systems that simultaneously use multiple types of engineered anchors in addition to His-tags (e.g. spycatcher [65,66], semiconductor-binding peptides [67], among many others) and/or systems that use the sequential genetic and nano-object functionalization of amyloid nanofibers to facilitate synergystic interactions favorable for catalysis. Considering, for example, the successful increase in the nano-object-mediated degradation effciency for CR and MO that we achieved by combining QD and Au NPs to exploit the charge-separation capacity of Au NPs, it is clear that these ideas and approaches deserve further exploration; we anticipate that substantial future advances with these flexible living catalysis systems will result both from the directed exploitation of their evolvability and from new developments in nano-objects with even higher surface-area-to-volume ratios.

## METHODS

### Strain construction

(i) MG 1655 *PRO* Δ*csgA ompR234* cells harboring a pZA-CmR-rr12-pL (tetO)-*csgA_His_* plasmid (Tc_Receiver_/CsgA_His_). *E. coli* MG1655 *PRO ∆csgA ompR234* was kindly presented by Timothy K. Lu’s lab, MIT. The construction details were in Lu’s previous work [68].

Note: *E. coli* Tc_Receiver_/CsgA_His_ meant MG 1655 *PRO* Δ*csgA ompR234* cells harboring a pZA-CmR-rr12-pL (tetO)-*csgA_His_* plasmid in this paper.

(ii) *E. coli* BL21(DE3) cell harboring a pACE-*hydA*-DC-*hydE*-DK-*hydF*-DS-*hydG* plasmid (BL21(DE3)/pAEFG).

Note: BL21(DE3)/pAEFG meant an *E. coli* BL21(DE3) cell harboring a pACE-*hydA*-DC *hydE*-DK-*hydF*-DS-*hydG* plasmid in this paper.

The strain information appears in Supplementary Table 3.

### Plasmid construction

(i) Construction of Tc-inducible plasmid (pZA-CmR-rr12-pL (tetO)-*csgA_His_*). pZA-CmR-rr12-pL (tetO)-*csgA_His_* plasmid was a generous gift from Dr. Timothy K. Lu’s research group at MIT. The detailed information for pZA-CmR-rr12-pL (tetO)-*csgA_His_* plasmid was described in detail in Lu’s previous work [68].

(ii) Construction of hydrogenase gene cluster (pAEFG:pACE-*hydA*-DC-*hydE*-DK-*hydF*-DS-*hydG*).

The ACEMBL system was a gift from Dr. Yan Nie at the Kornberg Lab, SIAIS, Shanghaitech University [61]. To create a single-fusion plasmid co-expression of the hydrogenase A (HydA) and its maturases (HydE, HydF and HydG), four genes encoding *hydA*, *hydE*, *hydF* and *hydG* originating from *Clostridium acetobutylicum* [62] were synthesized and optimized by GenScript for better heterologous expression in *E. coli*. To achieve the composite device with one acceptor (pACE) and three donor plasmids (pDC, pDK and pDS), the subcloning of *hydA* to pACE, *hydE* to pDC, *hydF* to pDK and *hydG* to pDS were performed individually through restriction-ligation or the Gibson Assembly approach to create pACE-*hydA*, pDC-*hydE*, pDK-*hydF* and pDS-*hydG*, respectively. These four plasmids were sequenced by Genewiz. The 10 μL of the mixture solution with an equivalent mole number of four recombinant plasmids pACE-*hydA*, pDC-*hydE*, pDK-*hydF* and pDS-*hydG* were fused for 1 h at 37°C with 0.5 μL of cyclization recombination enzyme (Cre) (New England Biolabs #M0298S) to produce a single-fusion plasmid pACE-*hydA*-DC-*hydE*-DK-*hydF*-DS-*hydG* (hereafter referred to as pAEFG) with four resistance genes including ampicillin resistance gene (AmpR), kanamycin resistant gene (KanaR), chloramphenicol resistant gene (CmR) and Spectinomycin resistant gene (SpeR). The primers and synthesized sequences are described in Supplementary Table 1. The sequencing results are described in Supplementary Figs 27–30. The agarose-gel electrophoresis result also confirmed the successful construction of the resulting plasmid pACE-*hydA*-DC-*hydE*-DK-*hydF*-DS-*hydG* (Supplementary Fig. 25b).

Special notes: The donor plasmids should be transformed into specific strains such as PirLC or PirHC (both containing a *pir* knock-in in their genomes) and the acceptor plasmid should be transformed into regular *E. coli* strains such as DH5α.

All gene sequences, protein sequences and plasmid information are summarized in Supplementary Tables 1, 2 and 4.

### Biofilm-cultivation conditions

Tc_Receiver_/CsgA_His_ seeds were inoculated from frozen glycerol stocks and grown for 12 h at 37°C with a 220-rpm shaking speed. Then, the bacterial cells were centrifuged and resuspended in the same volume of M63 growth medium with 1 mM MgSO_4_, 0.2% w/v glucose, 34 ug/mL chloramphenicol and 250 ng/mL tetracycline (Tc) as inducer (hereafter referred to as glucose-supplemented M63). The resuspended cultures were used as the seed solution and added to glucose-supplemented M63 growth medium at a volume ratio of 1:100. The experimental cultures were put into an incubator (Shanghai Yiheng) and cultivated for 48 h at 30°C without shaking.

The control experiments were performed under the same conditions without Tc in the culture medium.

### Hydrogenase expression and purification conditions

Protein expression: Seed cultures BL21(DE3)/pAEFG were inoculated from frozen glycerol stocks and grown in Luria-Bertani (LB) medium containing carbenicillin (50 μg/mL). The cultures were grown for 12 h at 37°C in a 14-mL tube, with constant shaking at 220 rpm; 5 mL of activated cells were added to 100 mL of LB broth containing carbenicillin (50 μg/mL) and ferric citrate (100 μM). These strains were grown to OD_600_ = 0.8 in LB broth at 37°C with a shaking speed of 220 rpm. Hydrogenase (HydA) and maturases (HydE, HydF and HydG) expression was induced with 1 mM isopropy-β-D-thiogalactoside (IPTG) for 1 h at 28°C with a shake speed of 100 rpm. The LB broth was then put into an anaerobic workstation (Whitley DG250) for 12 h and kept ready for the hydrogen-production experiment.

Protein purification: Cell pellets were collected by centrifugation at 4000 *g* for 20 min and resuspended with KPI buffer (3.16 g/L of KH_2_PO_4_, 5.16 g/L of K_2_HPO_4_, 33.75 g/L NaCl, pH = 7.20). Then, the cells were washed two or three times using KPI buffer. The cell wall was disrupted through ultrasonication with the lysis buffer (50 mM of Tris-HCl, 2 mM of EDTA, 100 mM of NaCl, lysozyme to 100 μg/ml, 0.1% Triton X-100, pH = 8.0) using a sonicator (Fisher Scientific, FIS#FB120220). The resultant solution was centrifuged at 15 000 *g* for 20 min. The supernatant was incubated with 5 mL of nickel resin (GenScript) for 0.5 h at room temperature. Resin beads were washed twice using KPI buffer and loaded onto the gravity column. Then, the resin beads were washed using washing buffer (20 mM imidazole, 300 mM NaCl and 50 mM potassium phosphate buffer, pH = 7.2) to remove nonspecific bound proteins. HydA proteins were collected with elution buffer (80 mM imidazole, 300 mM NaCl and 50 mM potassium phosphate buffer, pH = 7.2) for five consecutive steps. The purified proteins were then verified through SDS-PAGE analysis (Supplementary Fig. 25a).

### Decoration of nano-objects on *E. coli* biofilms

*In situ* one-pot cultivation method: Cell-seeds solution (Tc_Receiver_/CsgA_His_ cells) in glucose-supplemented M63 growth medium (at an initial density of 1.2 × 10^9^ cfu/mL), Co-NTA Au NPs, Ni-NTA Cd_0.9_Zn_0.1_S QDs or Ni-NTA CdSeS@ZnS QDs (~500 nmol/mL) aqueous solution and fresh glucose-supplemented M63 culture medium were mixed at a fixed volume ratio of 1:5:100. The mixture was thus used as experimental cultures and placed in a Petri dish for biofilm growth. Biofilm growth was induced in the presence of 250 ng/mL Tc for 48 h at 30°C. The nano-object-decorated *E. coli* biofilms were collected for subsequent catalytic reactions including PNP reduction and H_2_ evolution, as shown in Figs 2 and 4.

Post-cultivation modification method: Biofilms were grown as described above without nano-objects in the culture medium. Then, the specific amounts of nano-objects (Au NPs, Cd_0.9_Zn_0.1_S QDs or CdSeS@ZnS QDs) were mixed with biofilms. The mixed solution was centrifuged and the pellets were resuspended for subsequent catalytic reaction for the photodegradation of organic dyes, as shown in Fig. 3.

### PNP-reduction experiment

*E. coli* biofilms decorated with Au NPs were cultivated in a 12-well plate for 48 h at 30°C. The supernatant was discarded and functional biofilms were washed twice using ddH_2_O. Then, 2 mL of ddH_2_O, 600 μL of 0.1 M NaBH_4_ and 200 μL of 1 mM PNP were added to the wells with functional biofilms. The UV-Vis spectra were recorded using CYTATION (BioTek) every 10 min until the complete reduction of PNP. The reduction of PNP could be regarded as a pseudo-first-order reaction because the concentration of NaBH_4_ was significantly higher than the concentration of PNP. The kinetic equation could be written as:

$$-\ln{\left({A}_{400 nm}\right)}_{\mathrm{t}}={\mathrm{k}}^{\prime}\mathrm{t}-\ln{\left({A}_{400 nm}\right)}_0$$

This equation could be obtained based on the following deduction steps:

As first-order kinetics:

$$r=\frac{-\mathrm{d}\left[\mathrm{PNP}\right]}{\mathrm{d}t}={k}^{\prime}\left[\mathrm{PNP}\right]$$

where ${k}^{\prime }$is a rate constant of the reduction reaction. Upon integration, the equation becomes:

$$-\ln{\left[\mathrm{PNP}\right]}_t={k}^{\prime }t-\ln{\left[\mathrm{PNP}\right]}_0$$

[PNP]*_t_* is the concentration of PNP at time *t*.

[PNP]_0_ is the initial concentration of PNP.

[PNP] is proportional to *A*_400nm_, which is the absorption of PNP at 400 nm.

Then the last equation can thus be expressed as:

$$-\ln{\left({A}_{400\mathrm{nm}}\right)}_t={k}^{\prime }t-\ln{\left({A}_{400\mathrm{nm}}\right)}_0$$

Therefore, ${\mathrm{k}}^{\prime }$ can be obtained from the slope of the plot of –ln (*A*_400nm_) versus time, directly.

### Recyclable catalytic ability of *E. coli* biofilm-anchored Au NPs

Polypropylene (PP) blades were obtained from polyhedron empty balls, which were industrial water-purification materials. PP blades were grown in glucose-supplemented M63 medium with Au NPs (5.2 nm) so as to coat Au NPs on the surfaces of the PP blades; 9 mL of 0.1 M NaBH_4_ and 700 μL of 1 mM PNP were diluted to 50 mL with ddH_2_O and then the reaction mixture was added to the chromatographic column, which was filled with Au NP-decorated PP blades. The initial and final concentrations of PNP were detected through *A*_400nm_ and the reacted PNP was determined by subtracting *A*_final_ from *A*_initial_, where *A*_final_ is the absorption of PNP at 400 nm before the reaction and *A*_final_ is the absorption of PNP at 400 nm after a 16-min reaction.

### Loading capacity of biofilms for Au NPs

Biofilms were grown for 48 h at 30°C in the 12-well plate with 3 mL M63 culture medium in a well. The biofilms were collected (the wet mass was 3.1 mg), centrifuged and resuspended in 100 μL PBS solution for a well of a 12-well plate.

Then, the standard addition method was adopted to determine the loading capacity of biofilms for 5.2 nm Au NPs. An increasing volume (10 μL to 1 mL) of Au NPs (0.55 mg/mL) was added to the resuspended biofilms. Then, the solution stood for 10 min at room temperature for thorough binding of the Au NPs and biofilms. The absorbance at 518 nm for the supernatant of each sample was measured using a microplate reader (CYTATION, BioTek). The inflection point in the plot of absorbance versus the amount of Au NPs represented the saturated amount of Au NPs anchored to the biofilms (95.1 μg). The loading capacity of the biofilms for Au NPs could be determined as 30.4 μg/mg (Au NPs/biofilms) through the division of a saturated amount of Au NPs (95.1 μg) by the wet mass of the biofilms (3.1 mg).

### Photodegradation of organic dyes

(i) Photodegradation of CR: The biofilm-anchored nano-objects were achieved using a post-cultivation modification method. Tc_Receiver_/CsgA_His_ biofilms were first collected from 80 mL M63 medium after 3 day of cultivation at 30°C and then resuspended in the PBS solution (8 mL); 1.5 mL of QDs and a variable amount of Au NPs (0, 0.2 mL, 0.4 mL, 0.6 mL, 5.2 nm) were added and the mixture was then mixed for 20 min to allow thorough binding of the nano-objects to the biofilms. Afterwards, 0.9 mL of the CR solution (4 mg/mL) and 9 mL of the PBS solution were added. The reaction was performed under the illumination of a Xe lamp (CEL-HXF300) with a current of 15 A. The absorbance at 496 nm was recorded every 20 min by taking 800 μL of reaction solution using CYTATION (BioTek). All experiments had subtracted the background absorption of the biofilms at 496 nm. Recyclable photodegradation of CR with the biofilm-anchored NP structures: Biofilm-anchored QDs were applied for assessing the recycling performances of our hybrid catalytic systems. Specifically, the biofilm-anchored QDs were collected through centrifugation at 4000 *g* for 5 min after an 80-min reaction for the first cycle. A certain amount of CR (4 mg/mL) and PBS solution were added to the collected sample for photodegradation for another round. The absorption of the reaction solution at 496 nm reached the initial level of the first cycle. Three cycles of reactions were performed to demonstrate the robustness of our hybrid system.

(ii) Photodegradation of MO: The biofilm-anchored nano-objects were achieved using a post-cultivation modification method. Tc_Receiver_/CsgA_His_ biofilms were first collected from 120 mL M63 medium after 3-day cultivation at 30°C and then resuspended in the PBS solution (12 mL); 5 mL of Ni-NTA QDs and 1 mL of 250 mg/L MO were added. The reaction solution was supplemented with additional PBS solution to make a total volume of 25 mL. The reaction mixture was then put into a quartz container and stirred gently under the illumination of a xenon light (CEL-HXF300), with a light intensity of a current of 15 A. Absorbance at 464 nm was recorded every 90 min using a Cary 5000 (Agilent). For comparison of the catalytic efficiency between Tc_Receiver_/CsgA_His_ bound with QDs and Tc_Receiver_/CsgA_His_ bound with both Au NPs and QDs: The biofilm-anchored nano-objects were achieved using a post-cultivation modification method. Tc_Receiver_/CsgA_His_ biofilms were collected from 120 mL M63 medium after 3-day cultivation at 30°C, then resuspended in PBS solution (12 mL); 3 mL Ni-NTA QDs and 0.8 mL of 250 mg/L MO with or without 100 μL Au NPs were supplemented with additional PBS solution, to make a total 25 mL of reaction solution. The reaction mixture was put into a quartz container and stirred gently using illumination from a xenon light (CEL-HXF300) which had an intensity of a current of 15 A. Absorbance at 464 nm was recorded every 90 min using a Cary 5000 (Agilent).

(iii) The photodegradation of CR and MO followed first-order kinetics, and the kinetic equation could be written as:

$$-\ln \left({A}_t/{A}_0\right)={k}^{\prime }t$$

This equation could be obtained based on the following deduction steps:

As first-order kinetics:

$$r=\frac{-\mathrm{d}\left[\mathrm{dye}\right]}{\mathrm{d}t}={k}^{\prime}\left[\mathrm{dye}\right]$$

where *k*$^{\prime }$ is the apparent rate constant of the reduction reaction. Upon integration, the equation becomes:

$$-\ln{\left[\mathrm{dye}\right]}_t={k}^{\prime }t-\ln{\left[\mathrm{dye}\right]}_0$$

[dye]*_t_* is the concentration of the dye at time *t*.

[dye]_0_ is the initial concentration of the dye.

[dye] is proportional to its relevant absorbance.

The equation could be expressed as:

$$-\ln \left({A}_t/{A}_0\right)={k}^{\prime }t$$

Therefore, *k*$^{\prime }$ can be obtained from the slope of the plot of –ln (*A_t_*/*A*_0_) versus time, directly.

### Hydrogen-production experiment

The hydrogen-production reaction system is typically composed of 2.6 mL mixed solution of TEOA (1.5%), MV (5 mV) and glycerol (5%) in PBS solution (pH = 8.0), 200 μL of *E. coli* BL21(DE3)/pAEFG (wet cells, collected from 10 mL cultivation solution, were resuspended in 200 μL PBS solution) and biofilm-anchored CdSeS@ZnS QDs suspended in 200 μL PBS (the biofilm-anchored QDs were collected from 20 mL of M63 medium and resuspended in 200 μL of PBS solution). The reaction was performed in a 3-mL penicillin bottle with a small magnet stir bar. The hydrogen microsensor was inserted into the reaction solution. Hydrogen production was induced upon exposure of the reaction systems to light. The process was monitored using a microsensor Unisense H_2_-N and the data were collected by a Unisense microsensor multimeter hub. The light source used here was an artificial blue-light source with a current of 0.3 A.

### Biological and materials-characterization methods

(i) CR and crystal violet (CV) assay: Tc_Receiver_/CsgA_His_ biofilms were collected from 20 mL M63 medium that were cultivated for 72 h at 30°C, centrifuged and resuspended in 4 mL PBS solution (pH = 7.2). Congo red: CR solution (25 mg/mL, 10 μL) was added to the biofilm solution (1 mL) and stood for 10 min under ambient conditions. The absorbance at 495 nm was recorded for the supernatant after centrifugation designated as A_72_ using CYTATION (BioTek). The absorbance of 25 mg/mL CR was recorded and designated as *A*_0_. The amount of CR that bound to biofilms was calculated through the subtraction of A_72_ from A_0_. Crystal violet: CV solution (0.1%, 30 μL) was added to the biofilm solution (1 mL) and stood for 10 min under ambient conditions. The absorbance at 550 nm was recorded for the supernatant after centrifugation designated as A_72_ using CYTATION (BioTek). The absorbance of 0.1% CV was recorded and designated A_0_. The amount of CV that bound to the biofilms was calculated through the subtraction of A_72_ from A_0_. The control experiments were performed without Tc in the culture medium. Each test was repeated four times. The data are presented in Supplementary Fig. 5.

(ii) TEM**:** for TEM, a 10-μL droplet of the sample was directly deposited onto the TEM grid (Zhongjingkeyi Technology) for 3–10 min. Excess solutions were wicked away using pieces of filter paper and the samples were rinsed twice using ddH_2_O by placing 10 μL ddH_2_O onto the TEM grid and quickly wicking it off using filter paper. Samples were negatively stained using 5 μL of 2 wt% uranyl acetate for 1 min. Excess uranyl acetate was wicked off and the grid was desiccated for 20 min under an infrared lamp (Zhongjingkeyi Technology). TEM images were obtained on a FEI T12 transmission electron microscope operated at a 120-kV accelerating voltage. HRTEM, high-angle annular dark-field scanning TEM **(**HAADF STEM) and EDS mapping were performed on a JEM-ARM300F(w_d) electron microscope operating at a 300-kV accelerating voltage.

(iii) Nuclear magnetic resonance: for the nuclear magnetic resonance experiment, samples were dissolved in deuterated solvent. Data were collected at room temperature using an AVANCE III HD 500 MHz (Bruker).

(iv) Fluorescence spectra: the fluorescence spectra of free QDs, biofilm-anchored QDs and biofilm-anchored heterogeneous structures were recorded using a Fluorescence Spectrometer (HORIBA FL-3) with an excitation wavelength of 350 nm.

(v) Time-resolved fluorescence spectroscopy: biofilm-anchored QDs and biofilm-anchored heterogeneous structures composed of QDs and Au NPs were first collected and dried under ambient conditions. Time-resolved fluorescence spectra for the dried samples were recorded using a Fluorescence Spectrometer (HORIBA FL-3), with the excitation wavelength at 360 nm and fluorescence emission set at 461 nm. The data were fitted using DAS6 Analysis software (HORIBA Scientific).

(vi) UV-Vis spectra:UV-Visspectrawererecorded using a UV-Vis-NIR Spectrometer (Agilent Cary 5000).

## Supplementary Material

SI-Immobilization_of_functional_nano-objects_nwz104
