# Supplementary material for: Immobilization of functional nano-objects in living engineered bacterial biofilms for catalytic applications
Source: Natl Sci Rev. 2019 Jul 30;6(5):929–43. doi: 10.1093/nsr/nwz104 (PMC8291418; doi:10.1093/nsr/nwz104)
Supplement: SI-Immobilization_of_functional_nano-objects_nwz104 [file si-immobilization_of_functional_nano-objects_nwz104.docx]

**Supplementary Information**

**Immobilization of functional nano-objects in living engineered bacterial biofilms for catalytic applications**

Xinyu Wang^1,2,3†^, Jiahua Pu^1†^, Yi Liu^1^, Fang Ba^4^, Mengkui Cui^1^, Ke Li^1^, Yu Xie^1^, Yan Nie^5^, Qixi Mi^1^, Tao Li^1^, Lingli Liu^6^, Manzhou Zhu^6^, Chao Zhong^1★^

^1^Materials and Physical Biology Division, School of Physical Science and Technology, ShanghaiTech University, Shanghai 201210, China.

^2^Shanghai Institute of Ceramics, Chinese Academy of Sciences, Shanghai 200050, China.

^3^University of Chinese Academy of Sciences, Beijing 100049, China.

^4^School of Life Science and Technology, ShanghaiTech University, Shanghai 201210, China.

^5^Shanghai Institute for Advanced Immunochemical Studies (SIAIS), ShanghaiTech University, Shanghai 201210, China.

^6^College of Chemistry & Chemical Engineering, Anhui University, Hefei 230039, China.

^†^ These authors contributed equally to this work.

**^★^Corresponding author. Email:** [**zhongchao@shanghaitech.edu.cn**](mailto:zhongchao@shanghaitech.edu.cn)

**Table of Contents:**

Materials and Methods

Supplementary Figures 1 to 30

Supplementary Tables 1 to 4

Supplementary references

**Materials and Methods**

1. Synthesis of HS-NTA and nano-objects
2. Preparation of NTA-decorated nano-objects through ligand exchange

**1.** **Synthesis of HS-NTA and nano-objects**

**Reagents:**

Bromoacetic acid (Energy Chemical), N^6^-Carbobenzyloxy-L-lysine (Cbz-lys, 98%, J&K), NaOH (AR, Sinopharm), HCl (GR, Greagent), Pt/C (10%, Macklin), Methanol (CP, Sinopharm), pentane (AR, Sinopharm), NaHCO_3_ (Aladdin), 4-butyrothiolactone (sigma aldrich), acetic acid (Sangon Biotech). HAuCl_4_^.^3H_2_O (Energy Chemical), 1,2,3,4-tetrahydronaphthalene (tetralin, TCI), Oleylamine (OAm, 70%, Sigma-Aldrich), borane t-butylamine complex (TBAB, 97%, J&K), acetone (AR, Sinopharm), hexane (Titan), 1-Octadecene (ODE, TCI), CdO (99.99% pure, Aladdin), Zinc Oxide (ZnO, 99.7%, Adamas), zinc acetate (Zn(OAc)_2_, 99.999%, Aladdin), cadmium acetate (Cd(OAc)_2_, 99.995%, Sigma-Aldrich), trioctylphosphine (TOP, Sigma-Aldrich), 1-octanethiol (95%, Aladdin), oleic acid (OA, Sigma-Aldrich), 1-octadecene (ODE, >80% pure, Aladdin), Se powder (99.99%, Aladdin), S powder (Sigma-Aldrich), anhydrous ethanol (SRCR), chloroform (CHCl_3_, Greagent), dichloromethane (CH_2_Cl_2_, Energy Chemical). All reagents were employed without additional processes.

**Synthesis of (1S)-N-[5-[(4-Mercaptobutanoyl)amino]-1-carboxypentyl]iminodiacetic Acid (HS-NTA)**.

HS-NTA was synthesized referring to the modified literature procedure [1, 2].

Briefly, N^6^-Carbobenzyloxy-L-lysine (Cbz-lys) (8.4 g, 30 mmol) is reacted with Bromoacetic acid (8.34 g, 60 mmol) under aqueous solution of 2 M NaOH to give the product (1S)-N-(5-Carbobenzyloxyamino-1-carboxypentyl)iminodiacetic Acid (Cbz-NTA). Then Pd/C (0.6 g) is used to reduce Cbz-NTA (6 g, 15 mmol) under H_2_ atmosphere in 100 mL methanol to give the reduction product (1S)-N-(5-Amino-1-carboxypentyl)iminodiacetic Acid (NH_2_-NTA). NaHCO_3_ (1 g, 11.9 mmol) and 4-butyrothiolactone (0.6 g, 5.9 mmol) are added to 10 mL of aqueous solution with NH_2_-NTA (1 g, 3.8 mmol) to give the final product HS-NTA. ^1^H NMR (500 MHz, D_2_O): δ 3.93 – 3.41 (m, 5H), 3.26 – 2.94 (m, 2H), 2.70 (dd, *J* = 15.2, 8.1 Hz, 1H), 2.53 (dt, *J* = 30.9, 7.1 Hz, 1H), 2.39 – 2.22 (m, 2H), 2.00 – 1.64 (m, 5H), 1.62 – 1.10 (m, 4H) (Supplementary Fig. 1-2).

Au_102_ was synthesized according to a modified literature procedure [3].

HAuCl_4_·3H_2_O (209 mg, 0.50 mmol, a non-metal spatula should be used to weigh out HAuCl_4_·3H_2_O) was dissolved in nanopure H_2_O (19.0 mL, 0.028 M based on Au) in a 50 mL conical. In a separate 50 mL conical, p-mercaptobenzoic acid (292 mg, 1.89 mmol) was dissolved in a solution composed of nanopure H_2_O (18.43 mL) and 10 M NaOH (0.57 mL, 5.70 mmol). The pH of the resulting p-mercaptobenzoic acid/NaOH solution was determined to be >9. A 500mL Erlenmeyer flask was equipped with a stir bar and nanopure H_2_O was added to it (50 mL). In three separate beakers, the following solutions were dispensed: (1) 0.028 M HAuCl_4_ solution (17.8 mL, 0.5 mmol); (2) 0.10 M p-mercaptobenzoic acid / 0.30 M NaOH (15.5 mL, 1.5 mmol; (3) MeOH (75 mL). Under stirring, the HAuCl_4_ solution was poured into the 500mL Erlenmeyer flask (containing H_2_O), this was immediately followed by the addition of the p-mercaptobenzoic acid/NaOH solution. The solution turned from yellow to orange, then immediately afterwards the beaker of MeOH was also added to the 500 mL flask. The reaction was allowed to stir at room temperature for 1 h and then the solution turned from dark orange to light orange. After 1 hour, the solid NaBH_4_ (20.8 mg, 0.55 mmol) was added to the stirring reaction to reduce the polymer; the reaction continued to stir at room temperature for 17 h. The reaction turned black upon the addition of solid NaBH_4_. MeOH was added to the 500mL flask until the total volume was approximately 400 mL, then NH_4_OAc (20 mL, 5 M) was also added and stirred for another 20 min. The precipitation was got through centrifugation. The method of further purification was as follows: the obtained precipitate was washed by adding purified water for several times and split each into four, then 0.5 mL of 2 M NH_4_OAc solution and MeOH was added until the total volume in each conical was about 45 mL. The conicals were shaken to mix and were centrifuged at 4,000 rpm and 4ºC for 10 min. The resulting supernatant was decanted and the precipitates were dried in vacuum at room temperature for at least 6 h. The statistical diameter of the synthesized Au_102_ was 2.1 ± 0.5 nm (Supplementary Fig. 3a).

Au NPs synthesis. Au NPs were synthesized following reported protocols [2, 4].

Briefly, TBAB (43.48 mg, 0.5 mmol) was dissolved in the solution of tetralin (1 mL) and OAm (1 mL), then injected into a HAuCl_4_ solution where HAuCl_4_^.^3H_2_O (200 mg, 0.508 mmol) was dissolved in the solution of tetralin (10 mL) and OAm (10 mL) under ice bath and N_2_ flow. The reaction solution was stirred for 1 h under ice bath and N_2_ flow. Acetone was added to the reaction solution and centrifuged (8500 g, 10 min) to get the precipitate Au NPs. The product was dispersed in hexane (20 mL) and washed with 40 mL ethanol, then the product was centrifuged and redispersed in hexane (30 mL). The statistical diameter was 5.2 ± 0.5 nm (Supplementary Fig. 3b).

30 mg 5.2 nm Au NPs was dissolved in the mixture solution of ODE (10 mL), OAm (10 mL) and HAuCl_4_^.^3H_2_O (0.1 g, 0.254 mmol) under N_2_ flow. The reaction solution was heated to 80°C and kept at 80°C for 2 h under N_2_ flow. The reaction solution was cooled to room temperature and acetone (60 mL) was added to precipitate the product through centrifugation. The product was dispersed in hexane (20 mL) and washed with ethanol (40 mL) through centrifugation. The precipitation was redispersed in hexane (30 mL). The statistical diameter was 7.9 ± 0.6 nm (Supplementary Fig. 3c).

Synthesis of Zn_0.1_Cd_0.9_S QDs. QDs were synthesized following the previously reported protocols [2, 5]. A mixture of CdO (0.032 g, 0.25 mmol), ZnO (0.0405 g, 0.5 mmol), oleic acid (2.5 mL), and octadecene (20 mL) was added to flask and heated to 80°C. The flask was degassed under vacuum for 20 min at the same temperature and heated to 310°C under N_2_ flow. The temperature was lowered to 300°C when precursors were totally dissolved. The solution mixture of sulfur (0.016 g, 0.5 mmol) in octadecene (5 mL) was quickly injected into the reaction solution. Then the reaction solution was kept at 300°C for 3 h for subsequent nucleation and growth. Cold CHCl_3_ (20 mL) was added to quench the reaction. The resulting nanocrystals were washed with the mixture CHCl_3_/CH_3_CH_2_OH. The pellets were dispersed in CH_2_Cl_2_ (18 mL) kept for ligand exchange (Supplementary Fig. 3d).

Synthesis of CdSeS@ZnS QDs. CdSeS@ZnS QDs were synthesized following reported protocols [2]. A mixture solution of Cd(OAc)_2_ (258.6 mg, 1.1 mmol) and OA (5 mL) was first degassed and then heated to 150°C under N_2_ atmosphere. ODE (20 mL) was added and further heated to 305°C. Se (21.3 mg, 0.27 mmol) and S (0.96 mg, 0.03 mmol) dissolved in 0.3 mL of TOP was quickly injected into the reaction solution under N_2_ atmosphere. The reaction solution was maintained for 90 s at 150°C. 1-octanethiol (248.69 mg, 1.7 mmol) was added dropwise to the reaction solution and the temperature was lowered to 270°C. Then, Zn(OAc)_2_ (524.75 mg, 2.86 mmol) dissolved in a mixed solvent containing OA (4 mL) and ODE (1 mL), and S (216 mg, 6.75 mmol) dissolved in TOP (3.5 mL) solvent was sequentially injected into the above reaction solution. The reaction took place at a constant temperature of 270°C for 10 min. Ethanol was then added to precipitate the product and cooled down to room temperature. The synthesized QDs were dispersed in hexane and washed three times with hexane/ethanol, finally redispersed and stored in 20 mL of hexane (Supplementary Fig. 3e).

**2. Preparation of NTA-decorated nano-objects through ligand exchange**

**Ligand exchange was performed following our recently published literature [2].**

Ligand exchange for Au NPs. 2 mg/mL HS-NTA in phosphate buffered saline (PBS) solution (10 mL) was mixed with OAm-capped Au NPs (1 mL) (5.2 nm or 7.9 nm). The mixed solution was stirred overnight, then the aqueous layer was collected and filtered with 0.2 μm Acrodisc Syringe Filters (Pall) to get HS-NTA Au NPs.

Ligand exchange for Zn_0.1_Cd_0.9_S QDs. 2 ml of CH_2_Cl_2_ was added to Zn_0.1_Cd_0.9_S QDs (1 ml). HS-NTA solution (1 mL) (20 mg/ml in CH_3_OH, pH=12), along with 2 ml of CH_2_Cl_2_, was mixed with Zn_0.1_Cd_0.9_S QDs (1 mL). PBS (pH=7.4, 4 mL) was added after vortex for 2 min. The aqueous layer was diluted to 10 mL with PBS solution and filtered with 0.2 μm Acrodisc Syringe Filters (Pall) to get HS-NTA Zn_0.1_Cd_0.9_S QDs.

Ligand exchange for CdSeS@ZnS QDs. HS-NTA solution (1 mL) (20 mg/ml in CH_3_OH, pH=12) was mixed with CdSeS@ZnS QDs (1 mL). The pellets were dispersed in 10 mL of PBS solution after vortex for 3 min. The aqueous solution was filtered with 0.2 μm Acrodisc Syringe Filters (Pall) to get HS-NTA CdSeS@ZnS QDs.

Co-NTA Au NPs. 10 μL of 50 mM CoCl_2_ solution was added to 1 mL of HS-NTA Au NPs. The mixed solution was vortexed and filtered with 0.2 μm Acrodisc Syringe Filters (Pall).

Ni-NTA Zn_0.1_Cd_0.9_S QDs. 10 μL of 50 mM NiCl_2_ solution was added to 1 mL of HS-NTA Zn_0.1_Cd_0.9_S QDs. The mixed solution was vortexed and filtered with 0.2 μm Acrodisc Syringe Filters (Pall).

Ni-NTA CdSeS@ZnS QDs. 10 μL of 50 mM NiCl_2_ solution was added to 1 mL of HS-NTA CdSeS@ZnS QDs. The mixed solution was vortexed and filtered with 0.2 μm Acrodisc Syringe Filters (Pall).


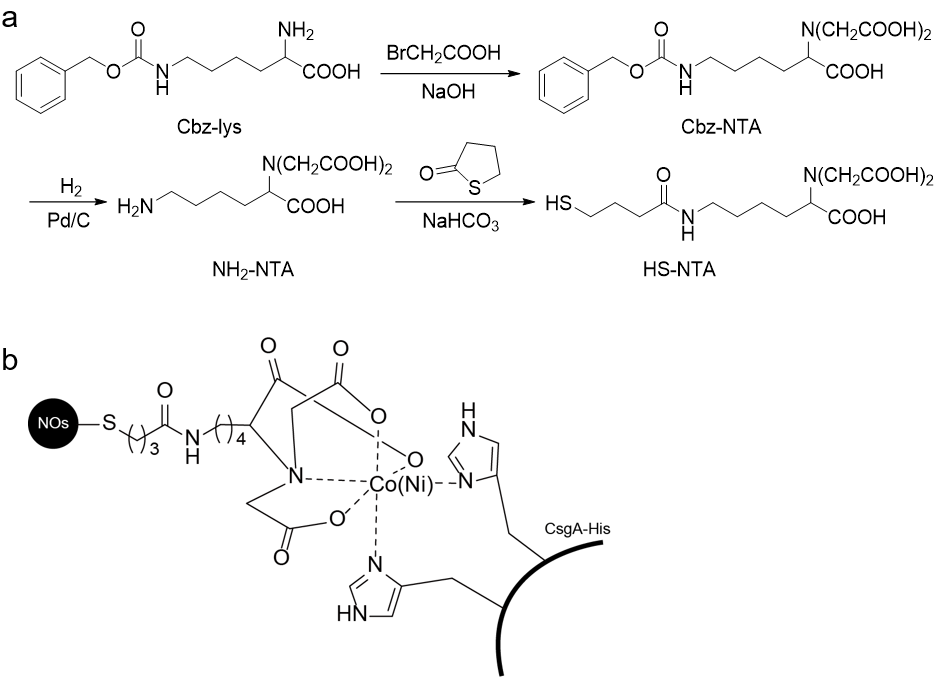


**Supplementary Figure 1. NTA-Metal-His coordination chemistry used for specific recognition and assembly of nano-objects along curli fibers displaying polyhistidine tag.** (a) Synthetic procedure of HS-NTA. (b) Schematic of molecular recognition between NTA ligand, covalently linked to nano-objects through a thiol group and imidazole group at the polyhistidine tags of CsgA_His_ protein.


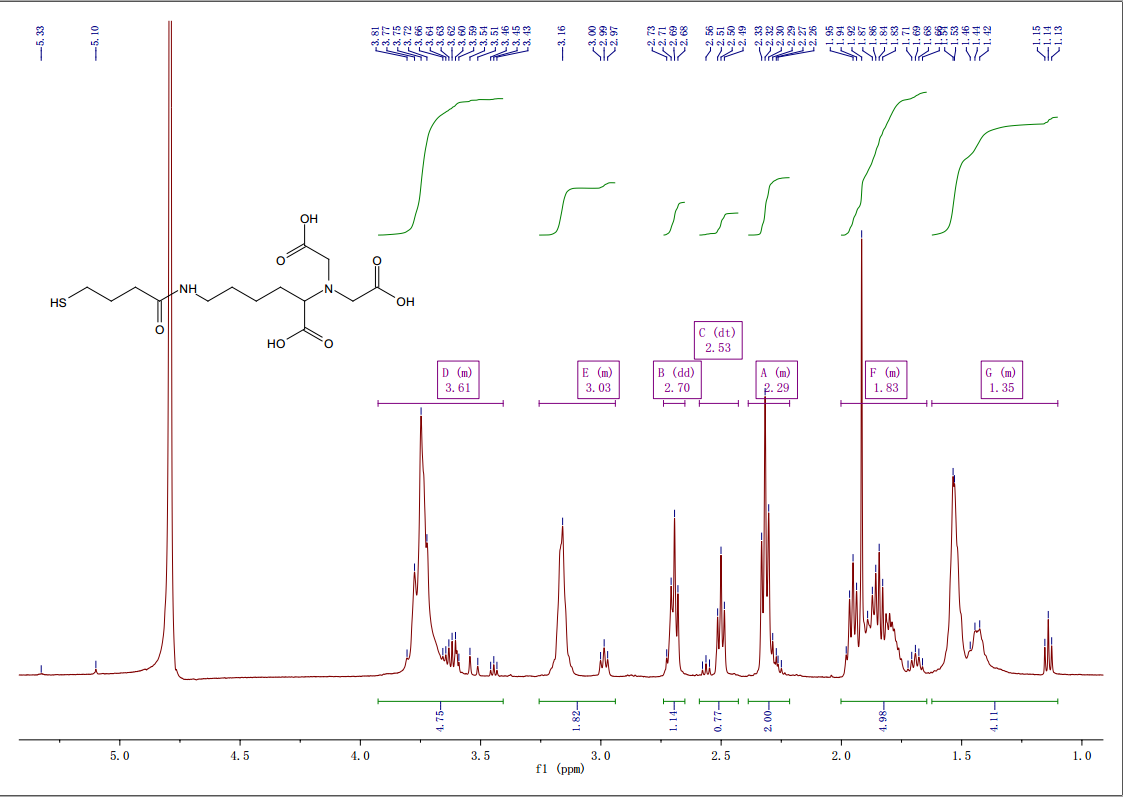


**Supplementary Figure 2. ^1^H NMR spectra of HS-NTA.**


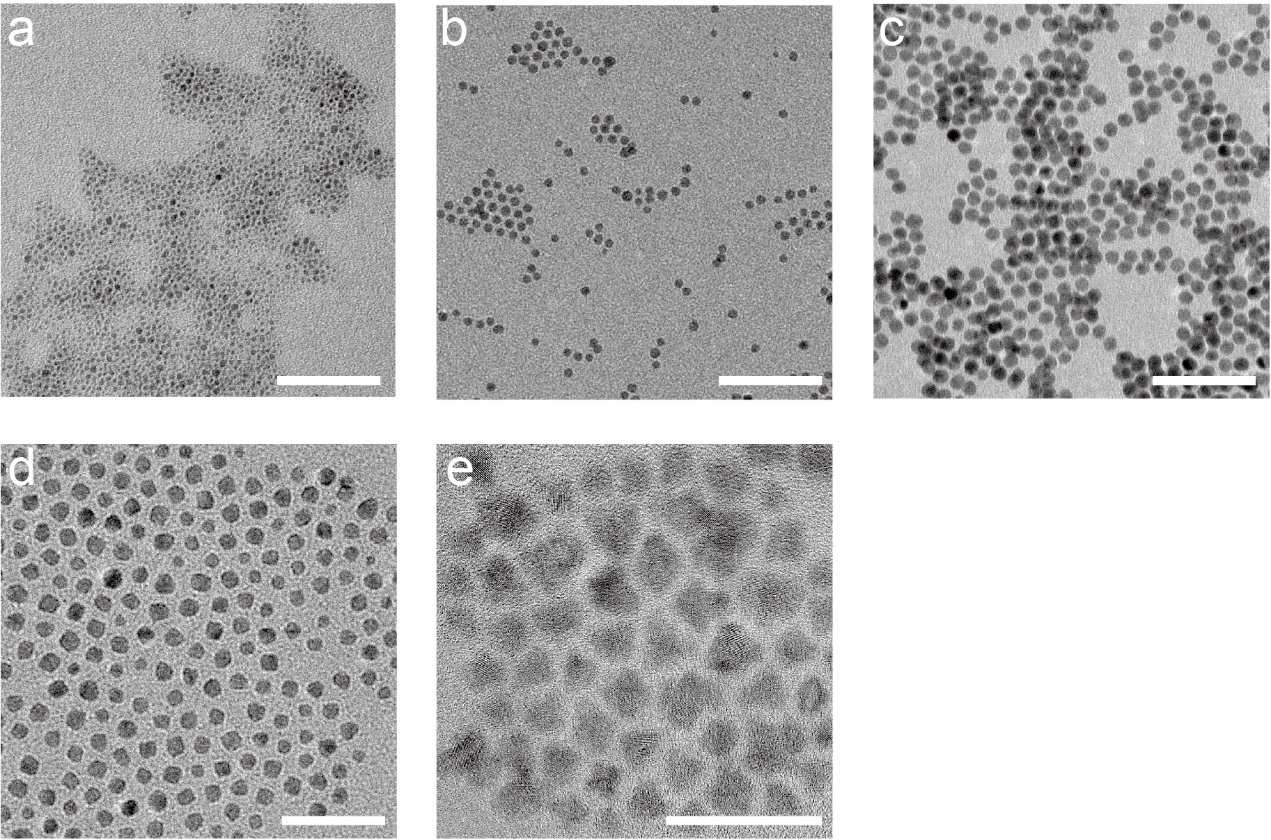


**Supplementary Figure 3. Morphological characterization of as-synthesized nano-objects.** (a) Au NPs with a diameter of 2.1 ± 0.5 nm. (b) Au NPs with a diameter of 5.2 ± 0.5 nm. (c) Au NPs with a diameter of 7.9 ± 0.6 nm. (d) Cd_0.9_Zn_0.1_S QDs. (e) CdSeS@ZnS QDs. All scale bars, 50 nm.


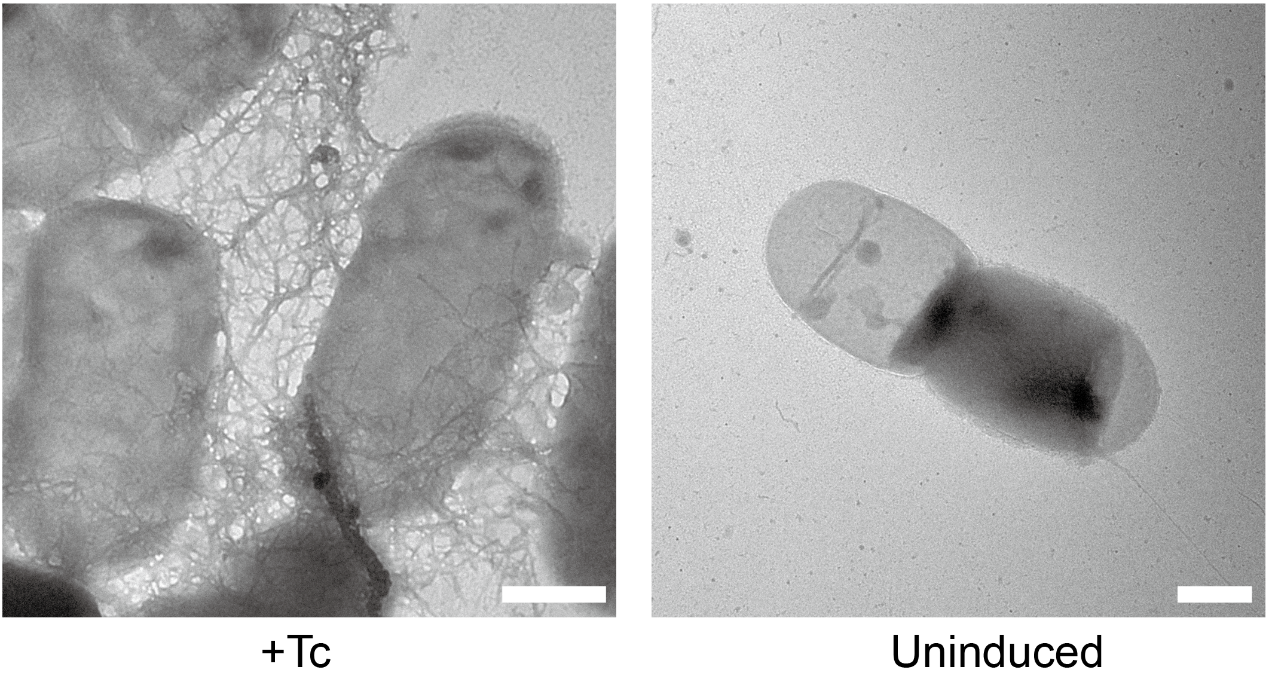


**Supplementary Figure 4. TEM images of *E. coli* biofilms with (left) and without Tc (right) induction.** The results clearly suggested that the biofilm formation was tightly regulated by the Tc-inducible riboregulator. All scale bars, 500 nm.


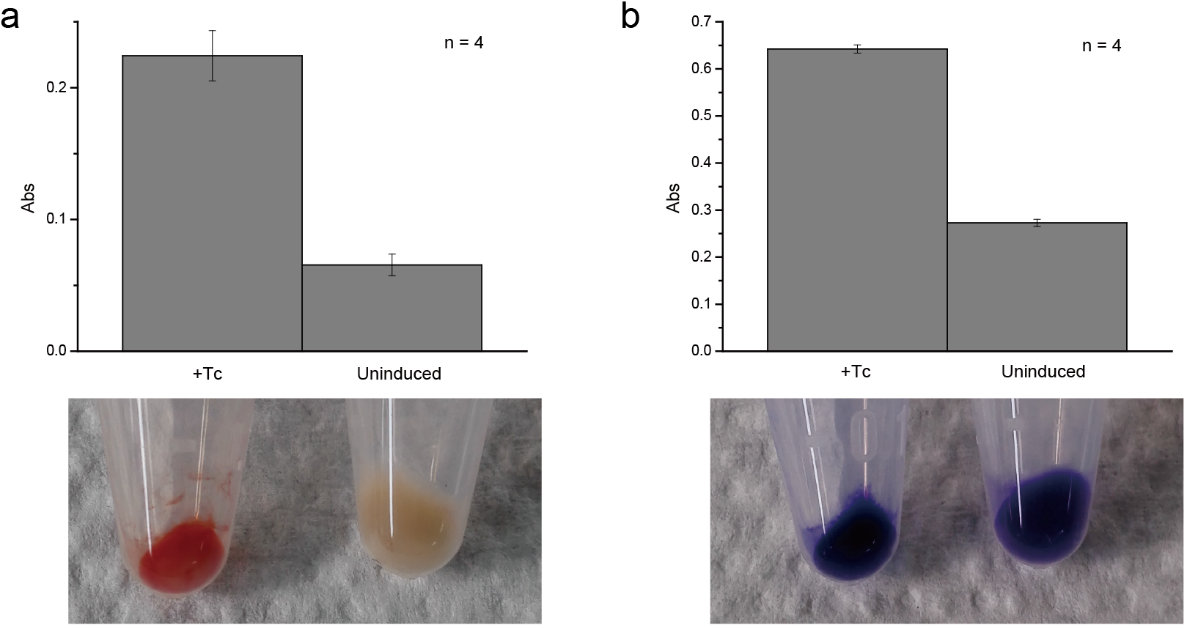


**Supplementary Figure 5. Congo red (CR) and crystal violet (CV) assay for *E. coli* biofilms.** (a) CR binding for *E. coli* biofilms, which clearly shown the CR would specifically bind to amyloid curli fibers. (b) CV staining of E. coli biofilms. The absorption was higher for biofilms due to the binding of CV to amyloid curli fibers. CR binding and CV staining were recorded through absorption of 495 nm and 550 nm, respectively.


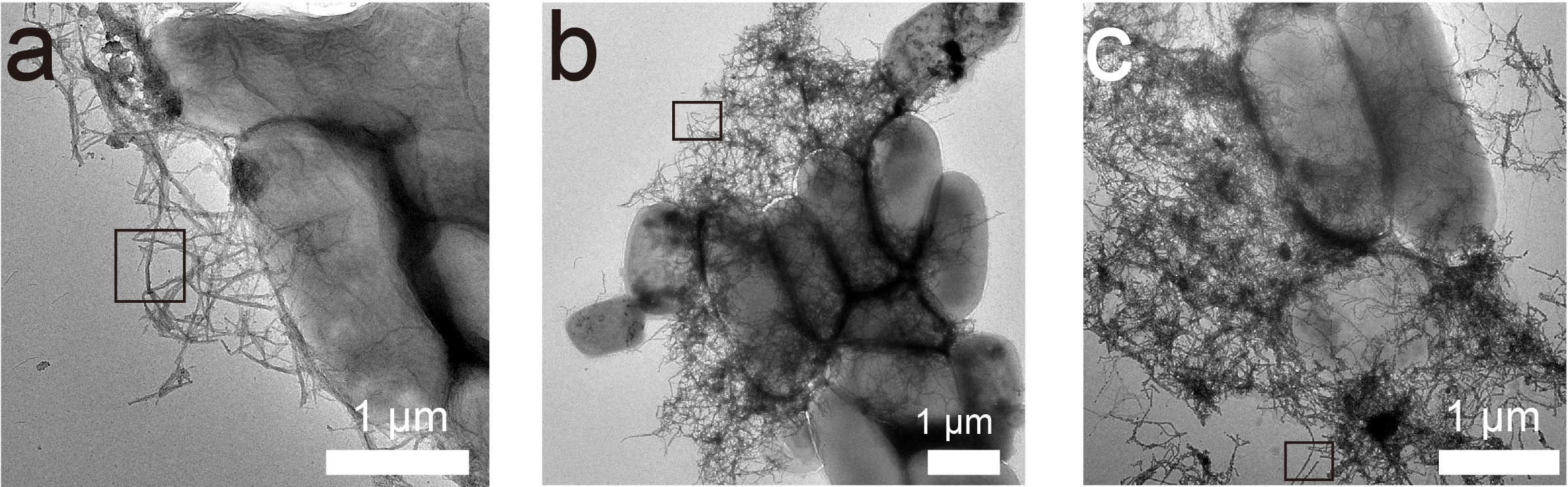


**Supplementary Figure 6. TEM images of *E. coli* biofilm-anchored Au NPs with diameter of 2.1 ± 0.5 nm (a), 5.2 ± 0.5 nm (b) and 7.9 ± 0.6 nm (c).** The enlarged images in the black boxes were displayed in Fig. 2f-h.


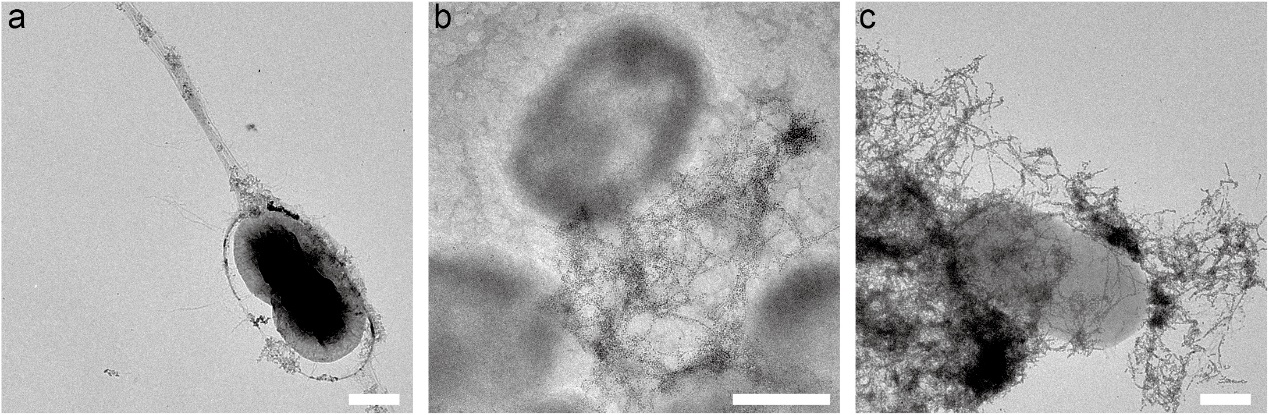


**Supplementary Figure 7. TEM images of *E. coli* biofilms decorated with Au NPs after 1^st^ catalytic p-nitrophenol reduction reaction.** Au NPs with diameters of 2.1 nm (a), 5.2 nm (b), 7.9 nm (c). The results clearly showed that after reaction, Au NPs were also tightly bound to curli fibers displayed by *E. coli*.


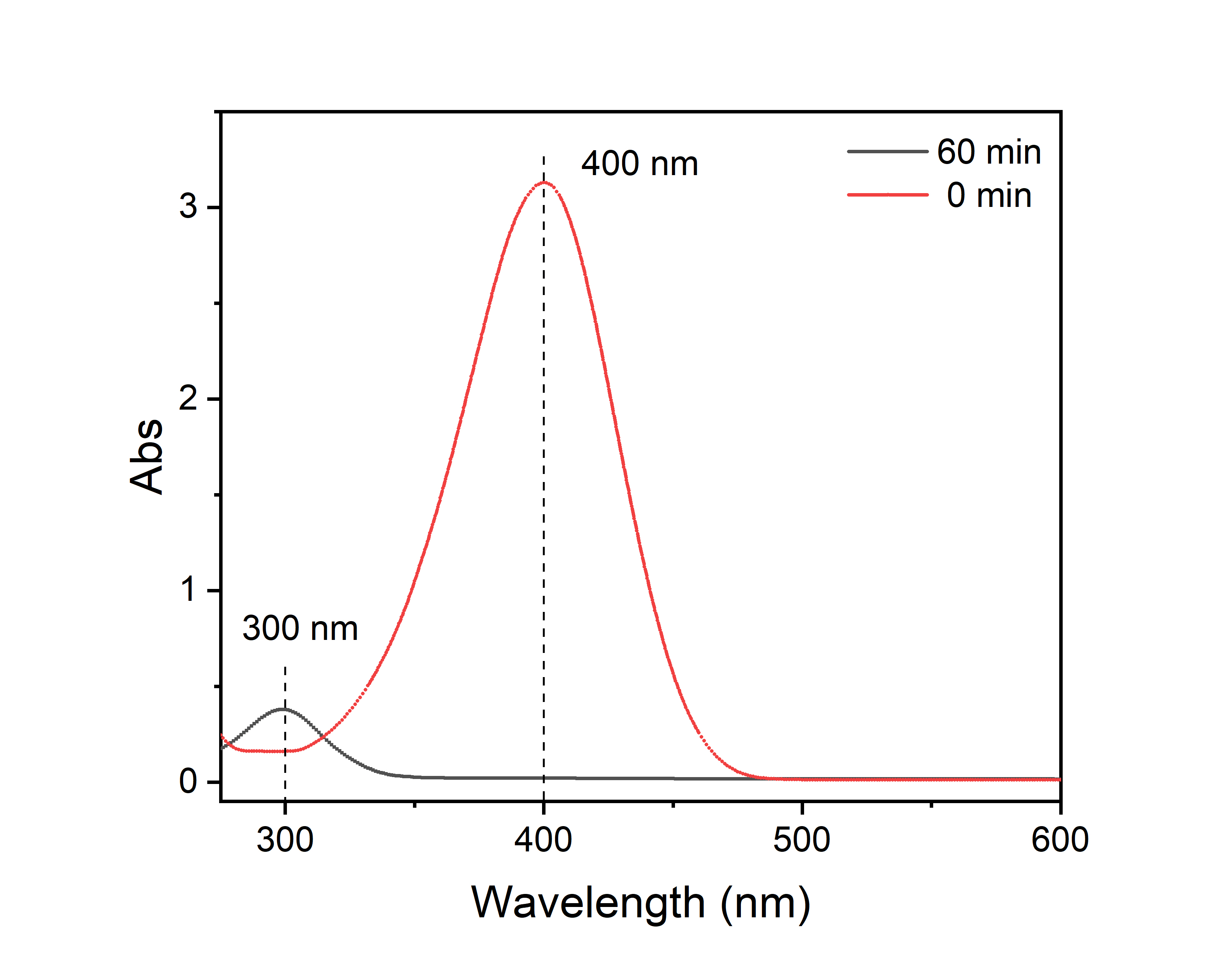


**Supplementary Figure 8. UV-Vis spectra monitoring the reduction of PNP to PAP.** The characteristic absorption peak of PNP was at 400 nm, while the characteristic absorption peak of PAP was at 300 nm. The data were collected using a cuvette with a standard optical path length of 1 cm (Agilent Cary 5000).

**
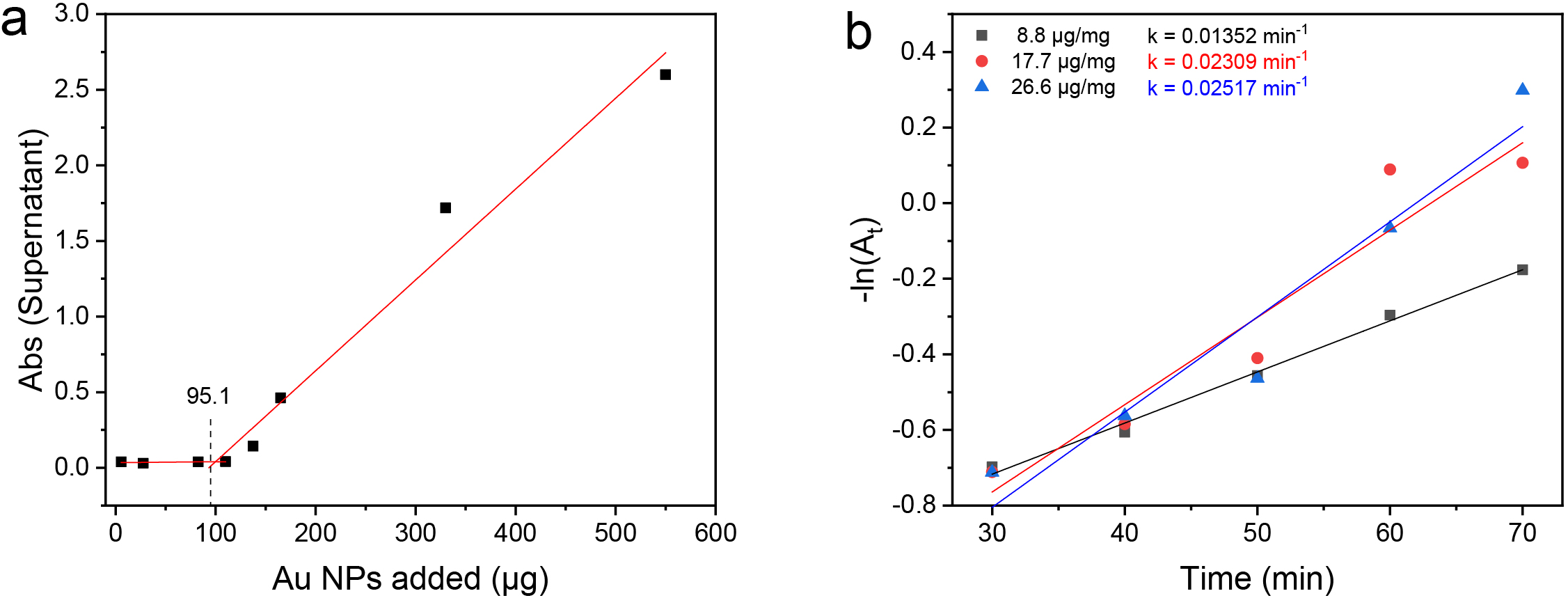
**

**Supplementary Figure 9.** (a) The adsorption of the supernatant at 518 nm for samples with growing amount of Au NPs added to the biofilms with a wet mass of 3.1 mg. (b) Rate constant with different loading of Au NPs on the biofilms.


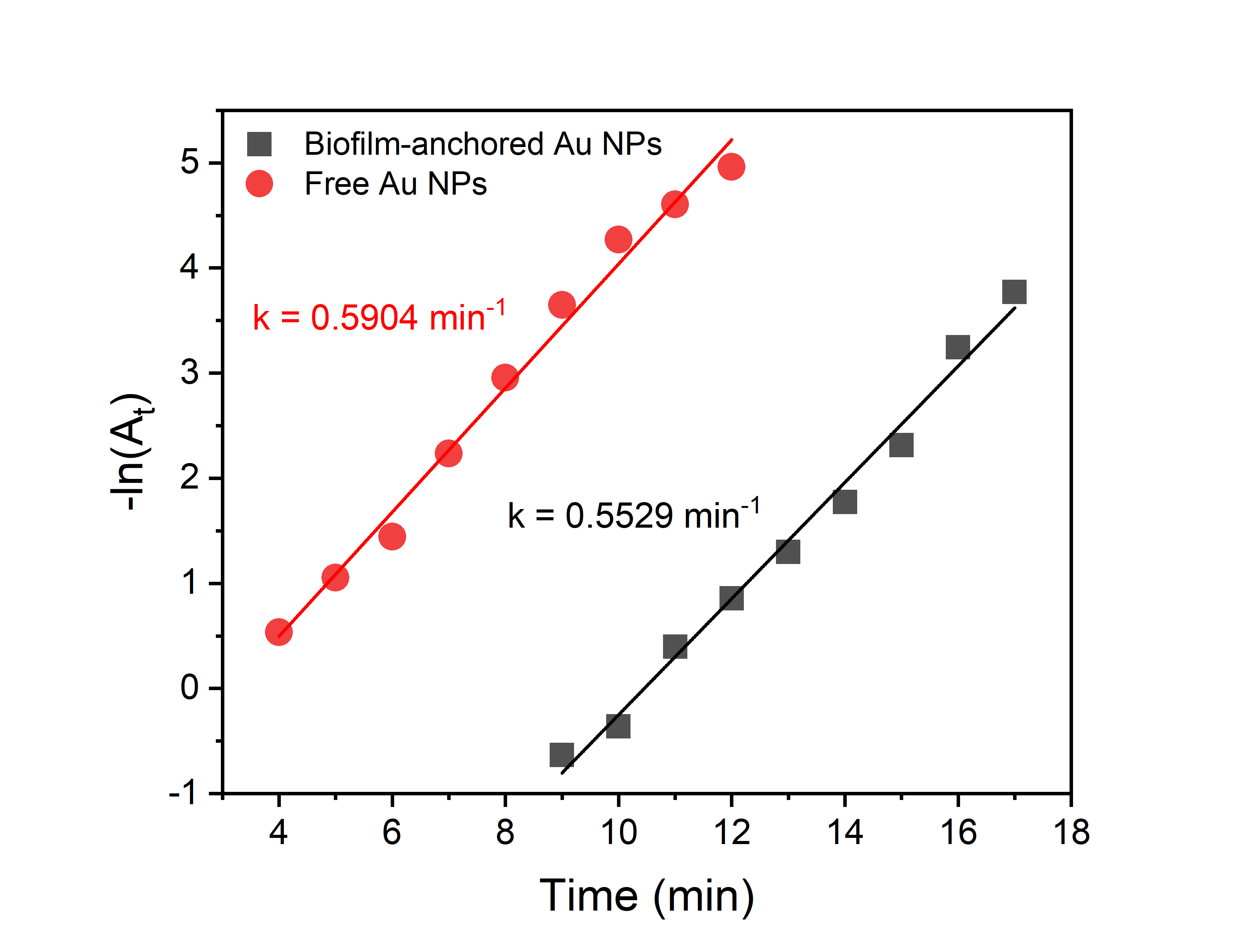


**Supplementary Figure 10. Rate constant for free Au NPs and biofilm-anchored Au NPs.** The red represented PNP reduction using free Au NPs as catalysts. The black represented PNP reduction using the freely suspended biofilm-anchored Au NPs as catalysts, in which the biofilm-anchored Au NPs scraped from the substrate were thoroughly mixed with the reaction solution.


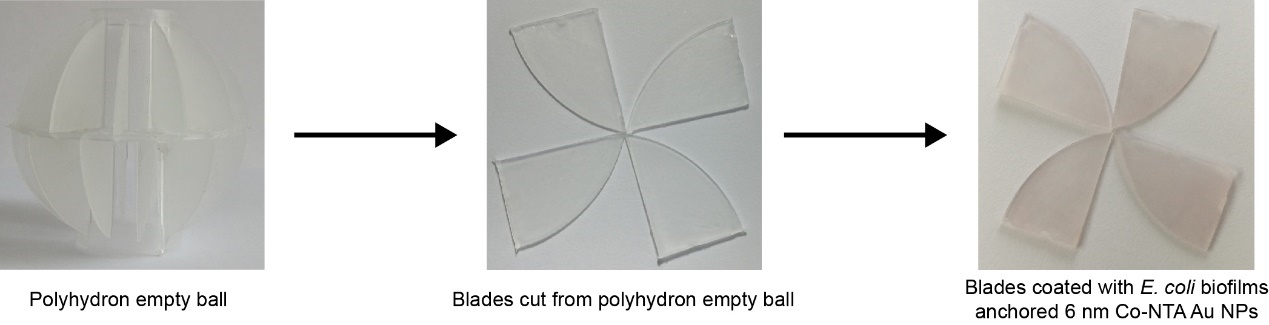


**Supplementary Figure 11. A three-step process to prepare blades coated with *E. coli* biofilm-anchored Au NPs (5.2 nm).** Blades were cut from polyhydron empty ball, the blades were then sterilized and added in glucose-supplemented M63 culture medium to produce *E. coli* biofilm-anchored Au NPs (5.2 nm) on the blades.





**Supplementary Figure 12. UV-vis spectrum of HS-NTA Cd_0.9_Zn_0.1_S QDs, which showed that quantum dots had a strong absorbtion in the region of visible light.**


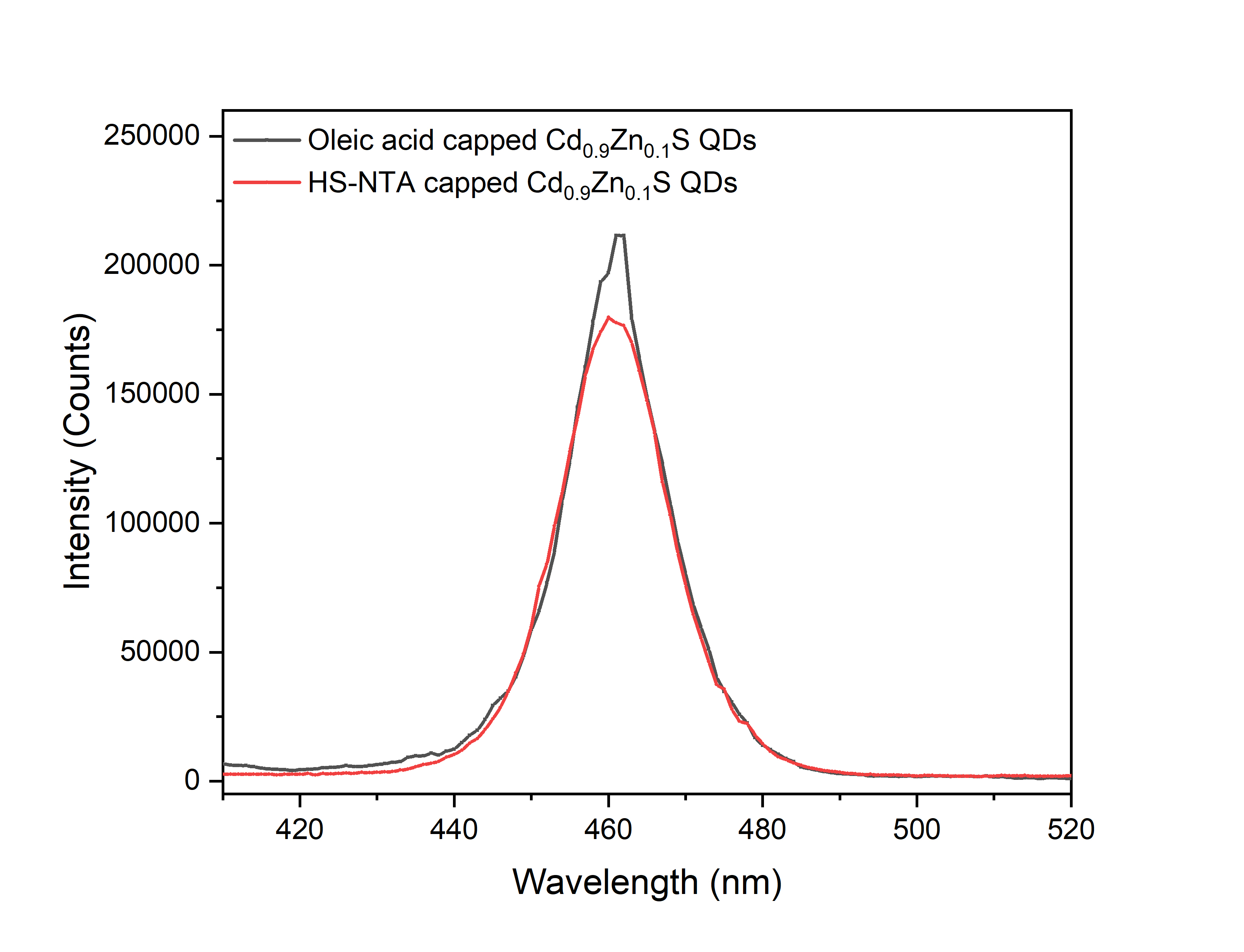


**Supplementary Figure 13. Fluorescence spectra of oil-soluble Cd_0.9_Zn_0.1_S QDs (black curve) and water-soluble Cd_0.9_Zn_0.1_S QDs (red curve).** The results showed that the emission peaks of QDs did not change after ligand change.


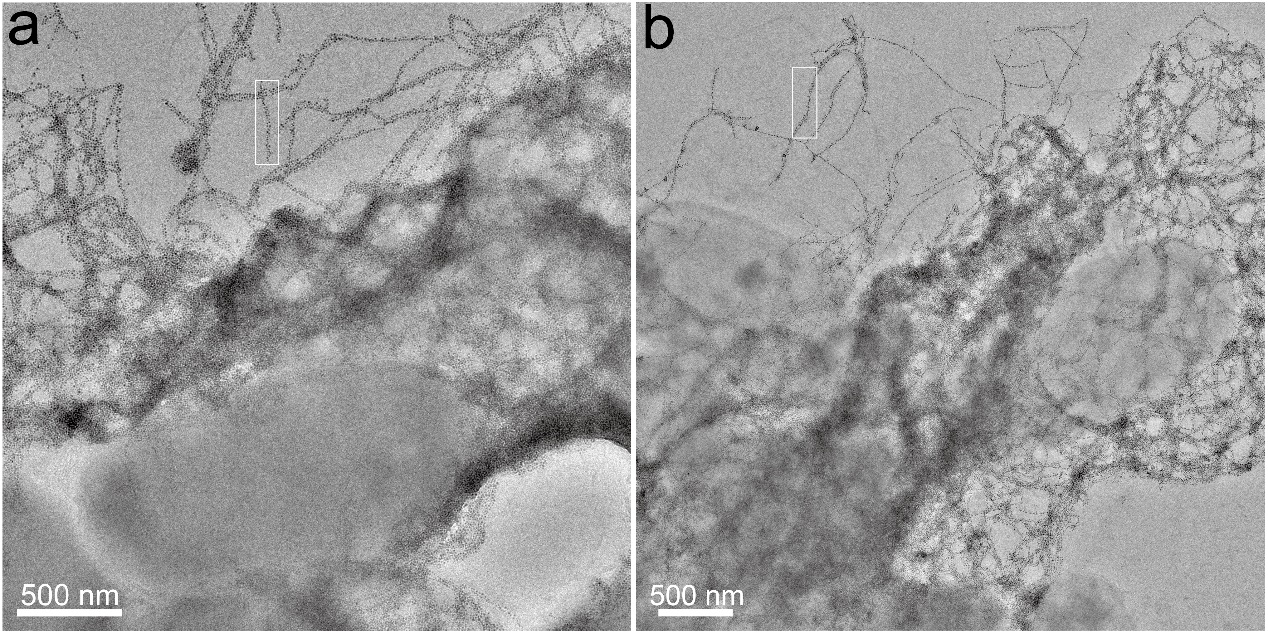


**Supplementary Figure 14.TEM images of Tc_Receiver_/CsgA_His_ biofims bound with Cd_0.9_Zn_0.1_S QDs (a) or both Au NPs (5.2 nm) and QDs (b).** The enlarged immages in the white box were displayed in Figure 3b and 3e.


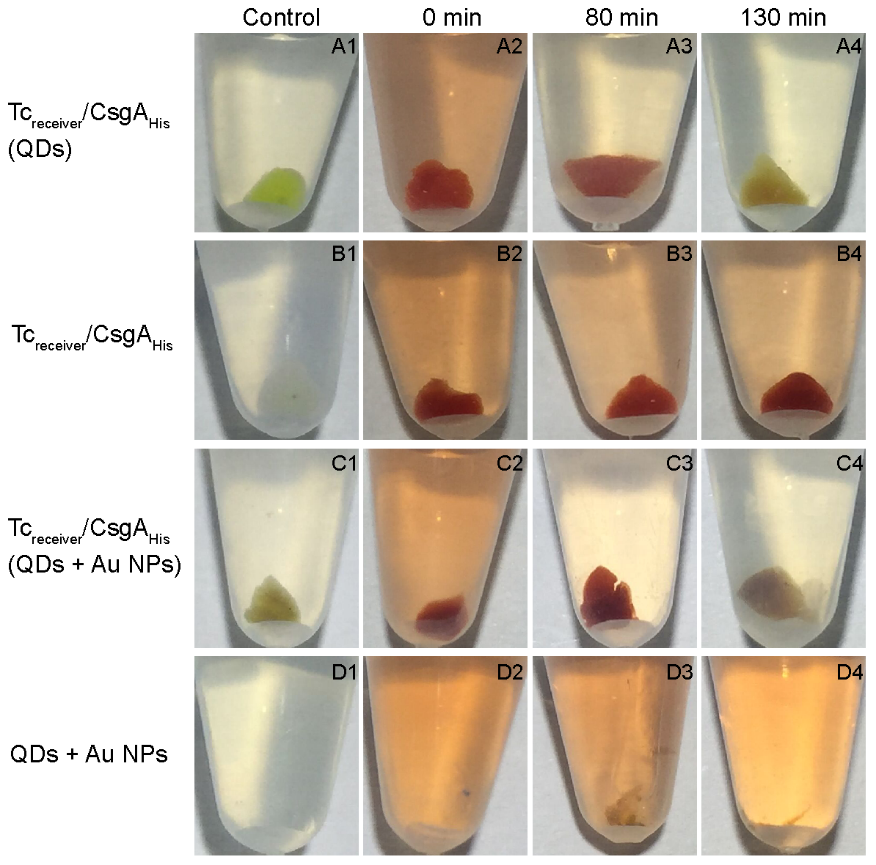


**Supplementary Figure 15. Photographs showing solution changes at different intervals with the different catalyst combinations.** The first column is the photographs of different catalysts after centrifugation without the addition of congo red. The catalytic effciency was significantly higher when Cd_0.9_Zn_0.1_S QDs were anchored to *E. coli* biofilms due to the enrichment of CR to curli fibers.

**
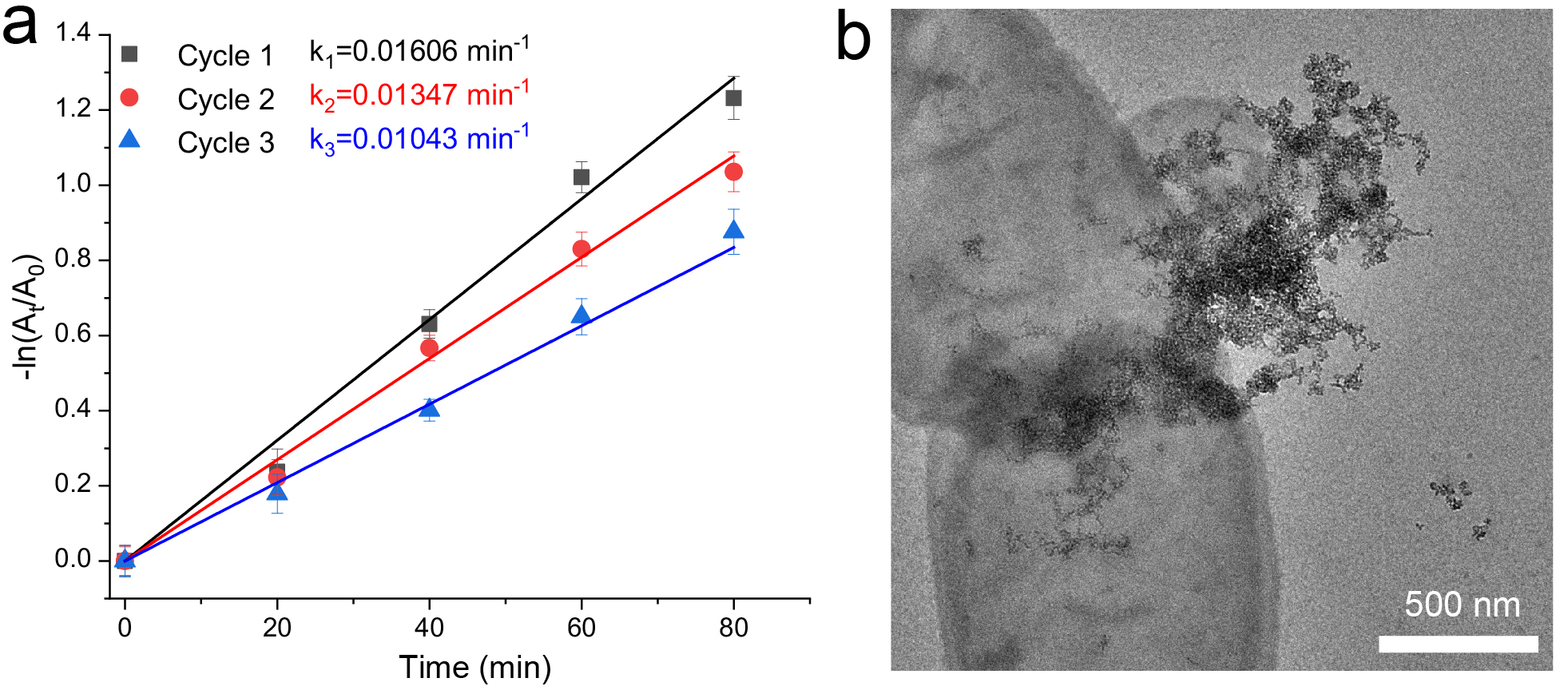
**

**Supplementary Figure 16. Robustness of biofilm-anchored Cd_0.9_Zn_0.1_S QDs.** (a) The repeated photodegradation of CR was conducted by adding new reaction solution to the hybrid structures. (b) TEM image of the hybrid structures afte three cycles of CR photodegradation.

**
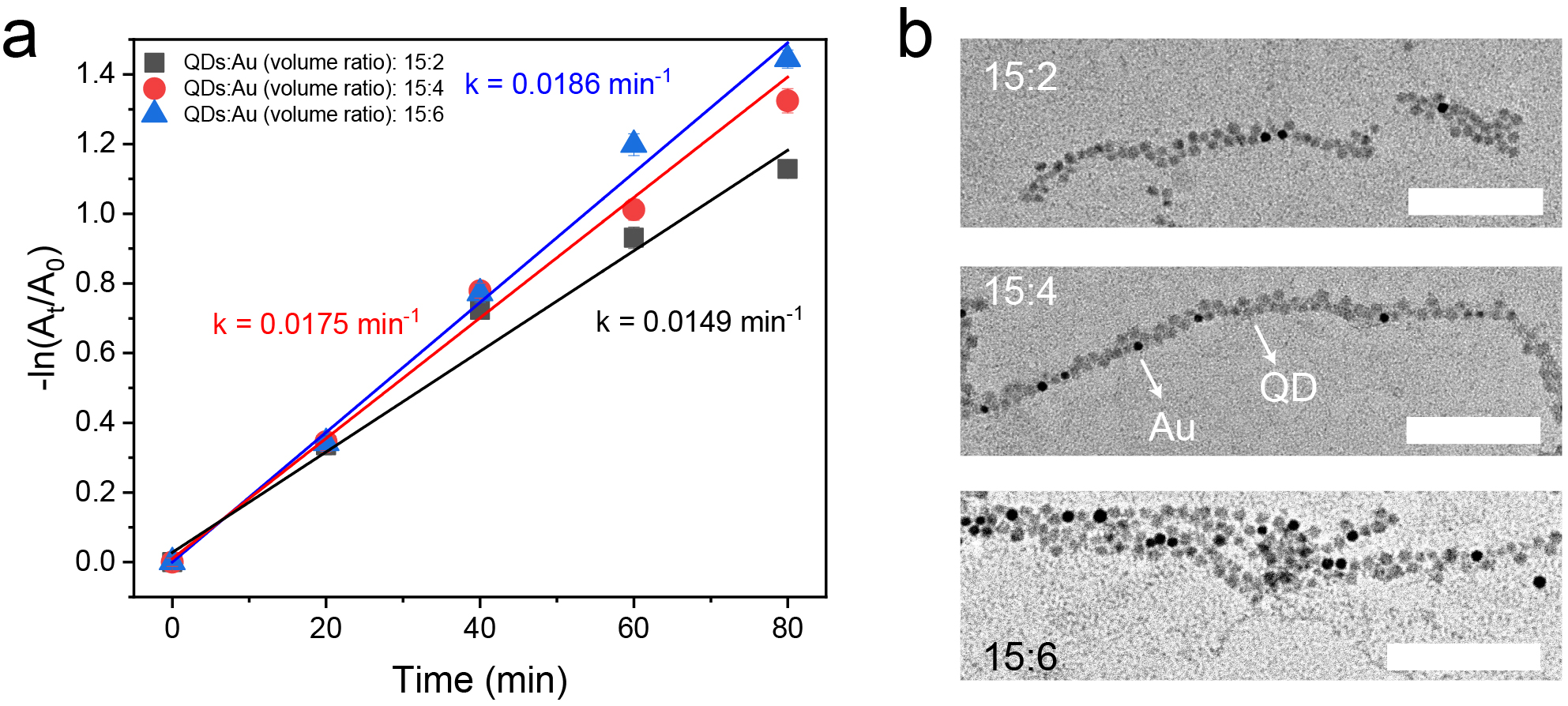
**

**Supplementary Figure 17.** (a) Photo-degradation of Congo red (CR) using *E. coli* biofilm-anchored heterogeneous structures as catalysts. The degradation rate increased with increased amount of Au NPs anchored on the biofilms. (b) TEM images of *E. coli* biofilm-anchored heterogeneous structures. Scale bars, 100 nm. Note: The initial volume ratio of QDs to Au NPs added to biofilm culture solution was 15:2, 15:4, and 15:6, respectively. The volume of QDs initially used was fixed at 1.5 mL.


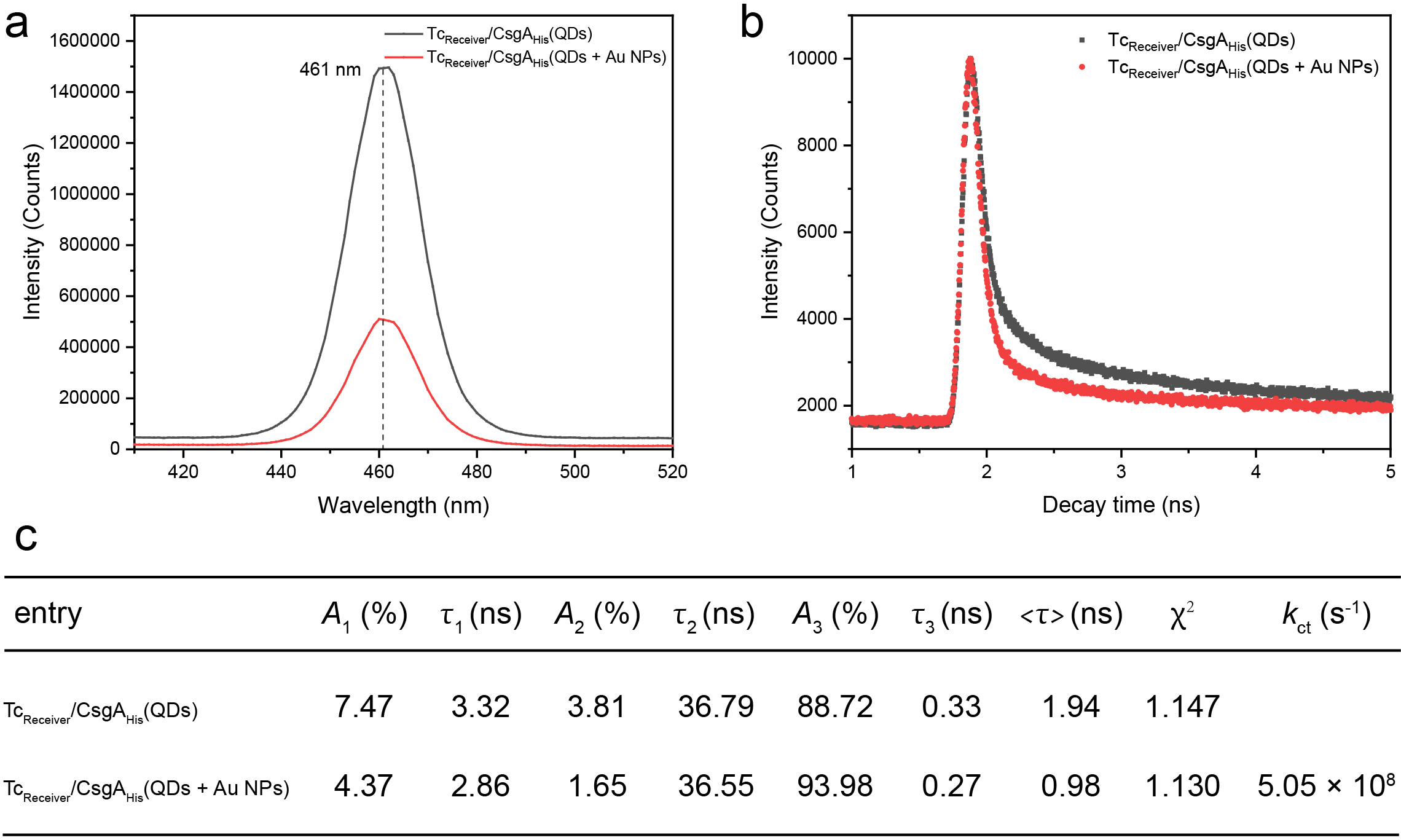


**Supplementary Figure 18.** Stead-state fluorescent spectra (a) and time-resolved fluorescent spectra (b) for the different biofilm-anchored nano-objects systems. (c) Fitting results of the time-resolved fluorescent spectra for the different biofilm-anchored nano-objects systems. Tc_Receiver_/CsgA_His_(QDs) represents biofilms decorated with Cd_0.9_Zn_0.1_S QDs, while Tc_Receiver_/CsgA_His_(QDs + Au NPs) represents biofilms decorated with both Cd_0.9_Zn_0.1_S QDs and Au NPs. The initial volume ratio of QDs to Au NPs was 15:6. The estimation method for the rate constant of charge transfer was adopted from a previously reported method [6]. Note: the decay curves were fitted with a triexponential function to generate an intensity-average emission lifetime <τ>.


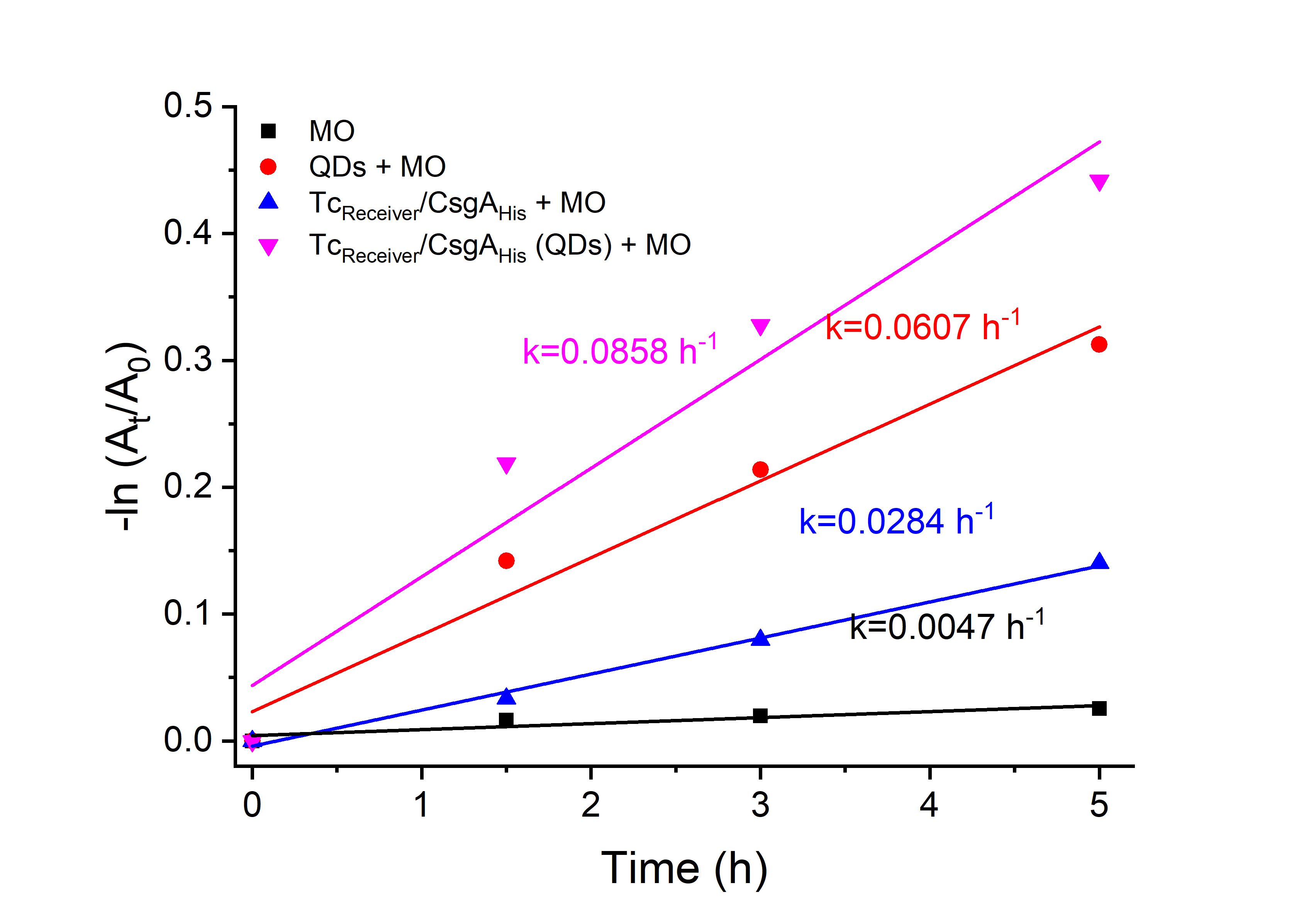


**Supplementary Figure 19. Photo-degradation of methyl orange (MO) using *E. coli* biofilm-anchored nano-objects as catalysts.** The highest catalytic effciency was obtained based on *E. coli* biofilm-anchored Cd_0.9_Zn_0.1_S QDs owing to the synergistic contributions from both QDs and *E. coli*.


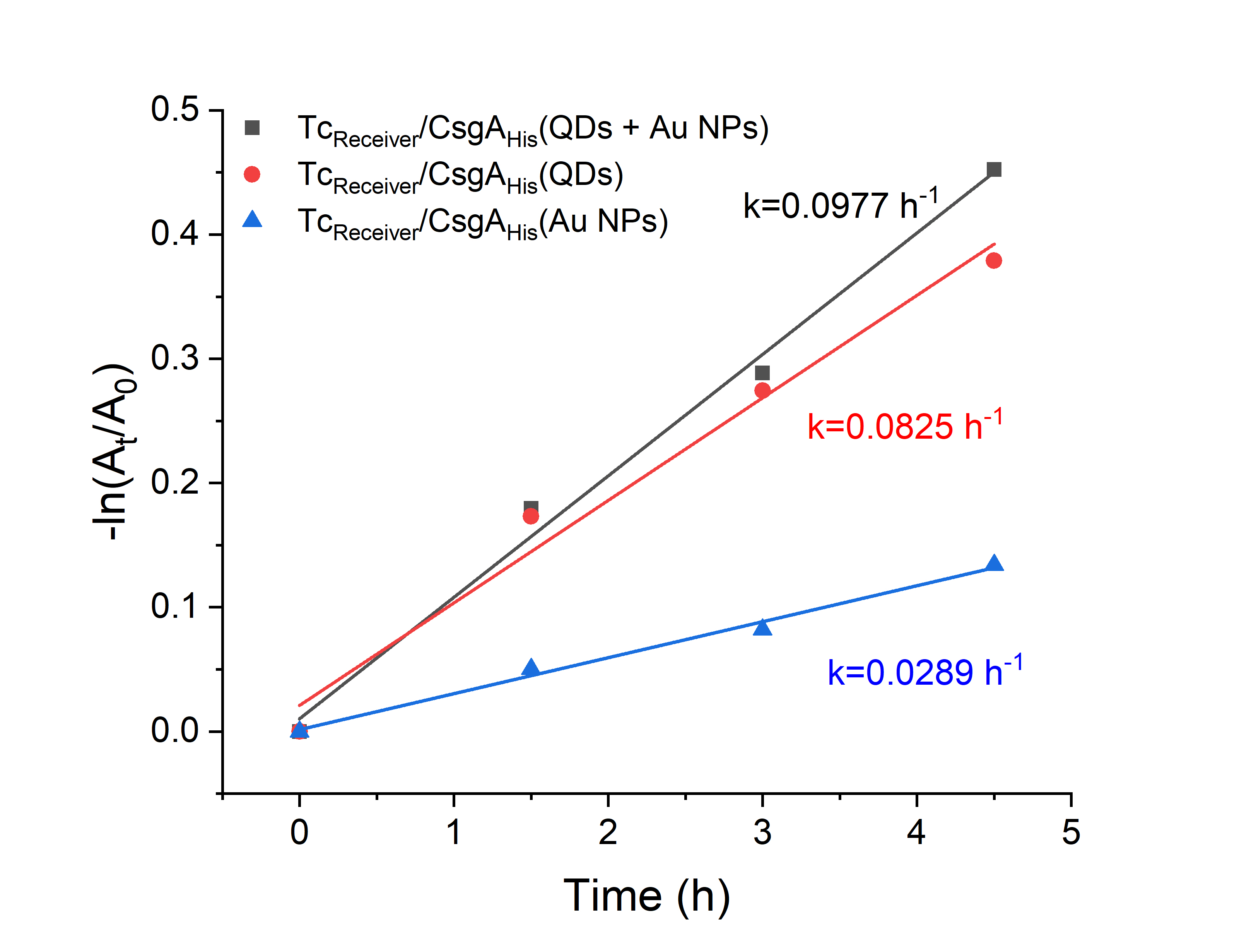


**Supplementary Figure 20. Photo-degradation of MO using *E. coli* biofilm-anchored NOs (Au, QDs or hybrid structures) as catalysts.** *E. coli* biofilms anchored with both Cd_0.9_Zn_0.1_S QDs and Au NPs (5.2 nm) showed enhanced catalytic activity compared to *E. coli* biofilms anchored with only Zn_0.1_Cd_0.9_S QDs or Au NPs. The enhanced catalytic activity was attributed to electron transfer from Zn_0.1_Cd_0.9_S QDs to Au NPs.


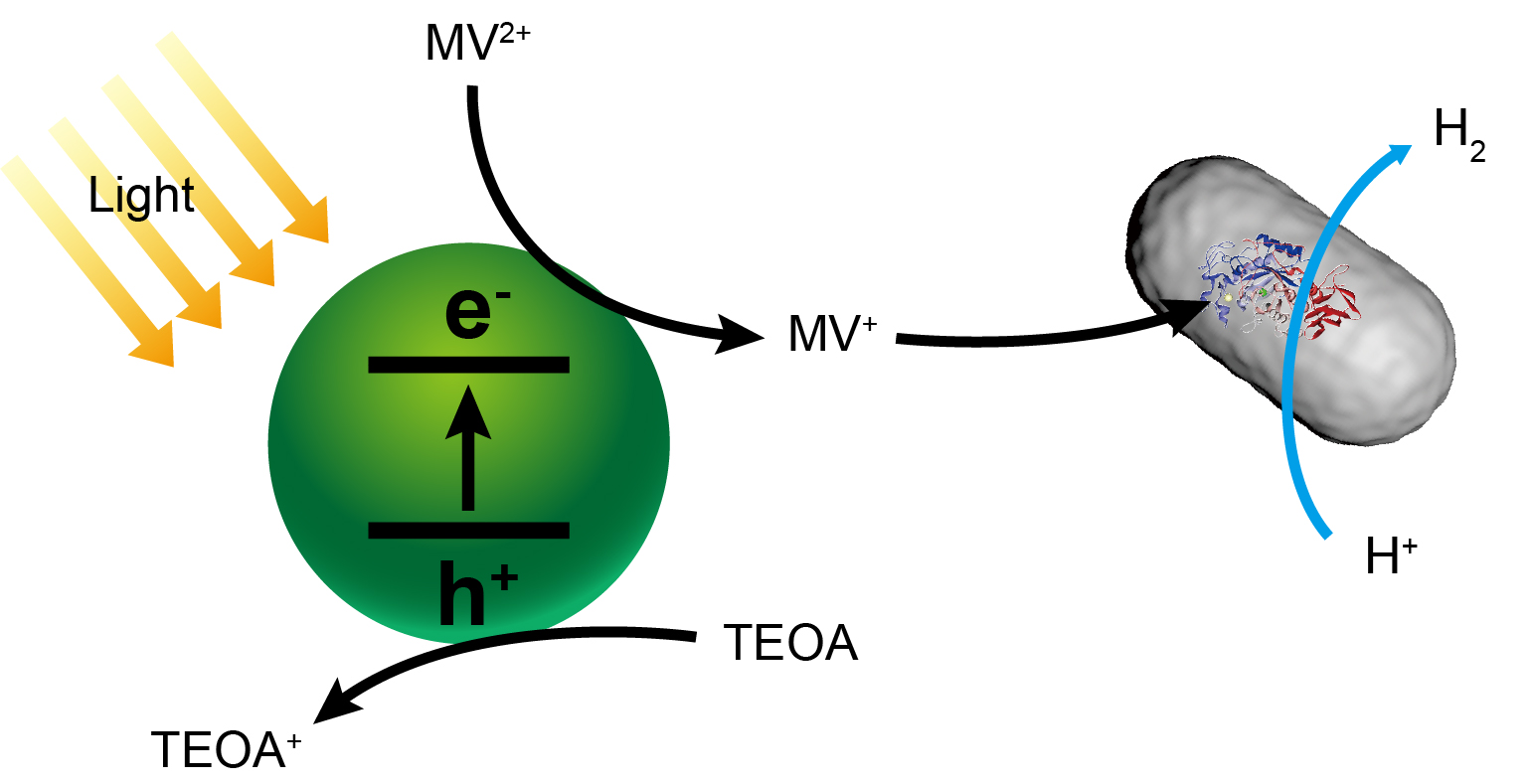


**Supplementary Figure 21. Schematic of electron transfer pathway for photo-generated electrons.** Uppon illumination for QDs, the photo-generated electrons were transferred to MV to form MV radicals. Then MV radicals were diffused in solution and transferred to intracellular hydrogenase for the production of H_2_. The photo-generated holes were quenched by TEOA.


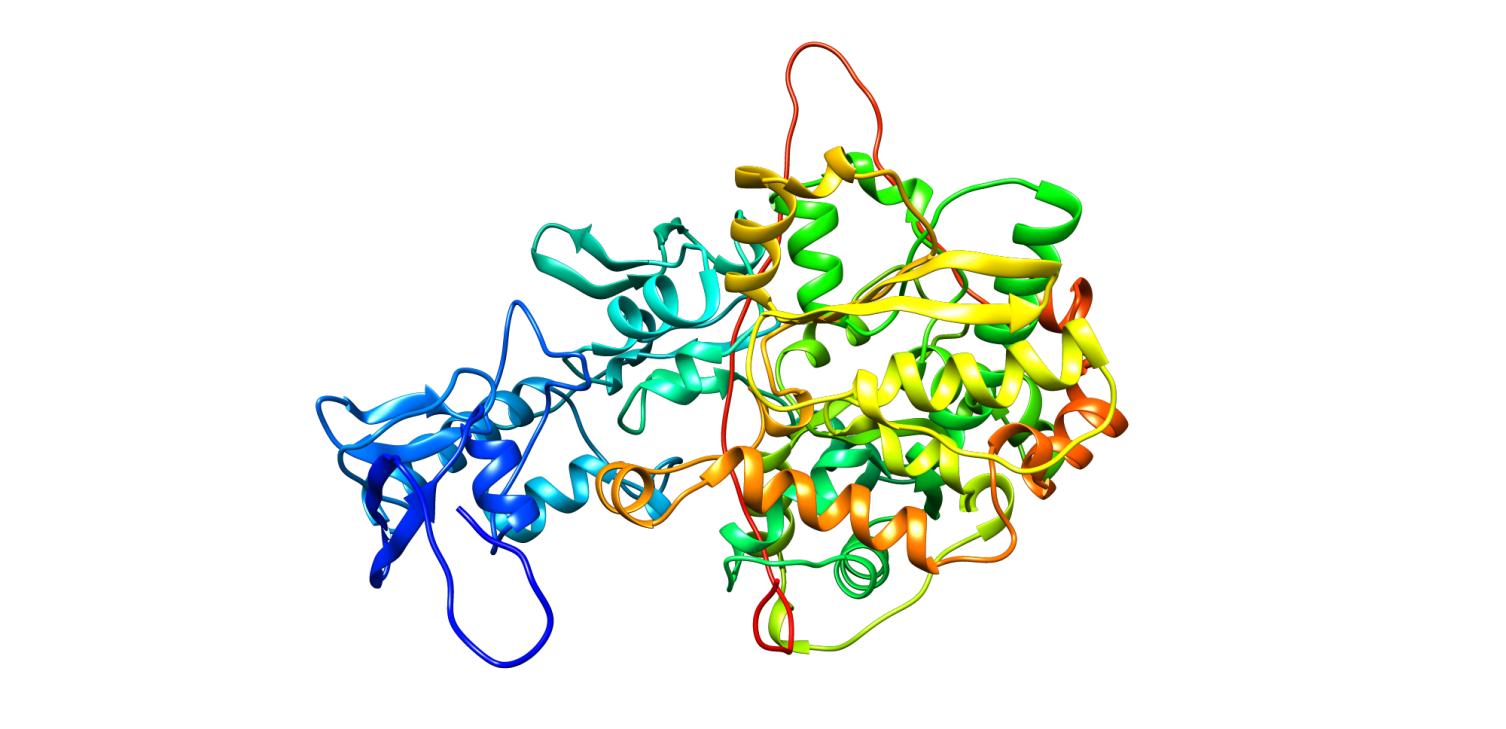


**Supplementary Figure 22. HydA strucure predicted through RaptorX [7].**


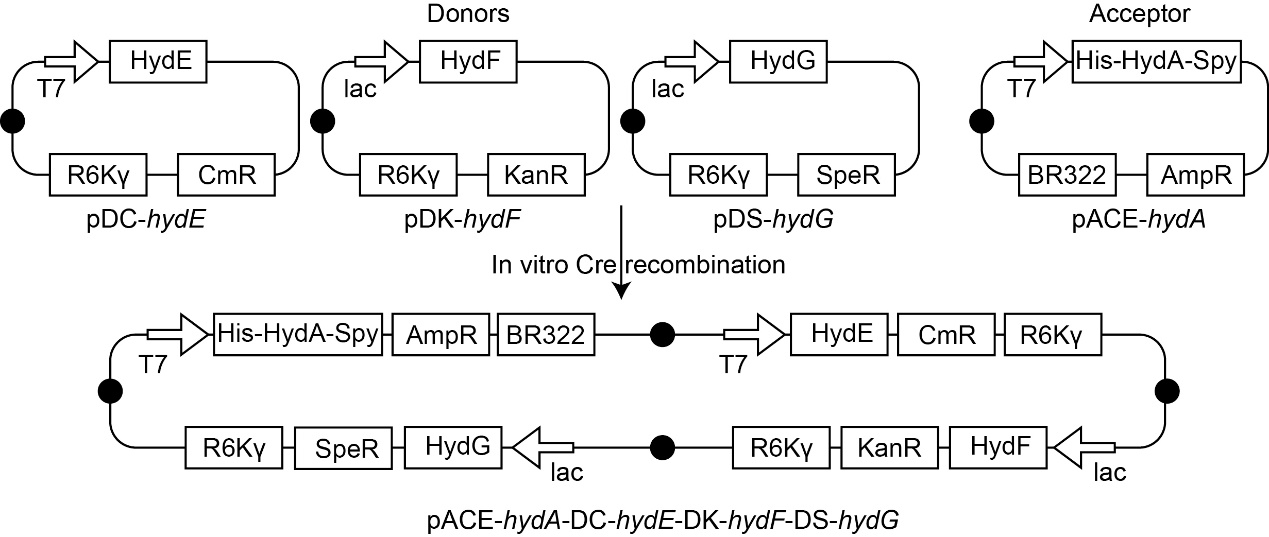


**Supplementary Figure 23. ASSEMBL system for the construction of a single fusion plasmid pAEFG co-expressing hydrogenase HydA and maturases HydE, HydF and HydG.** Three donor plasmids and one acceptor plasmid were recombined through *in vitro* Cre enzyme.


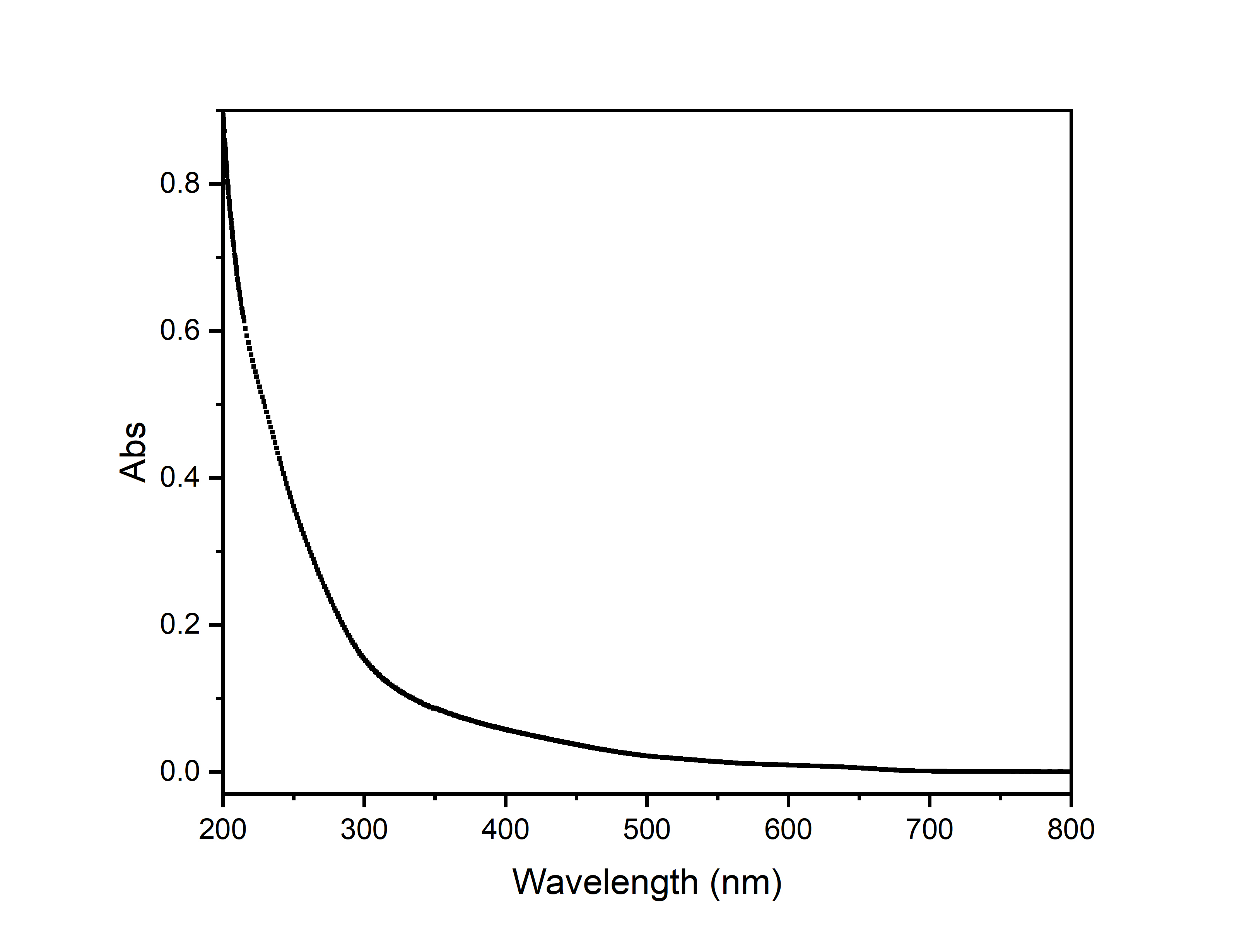


Supplementary Figure 24. The absorbance spectra of NTA capped CdSeS@ZnS QDs.


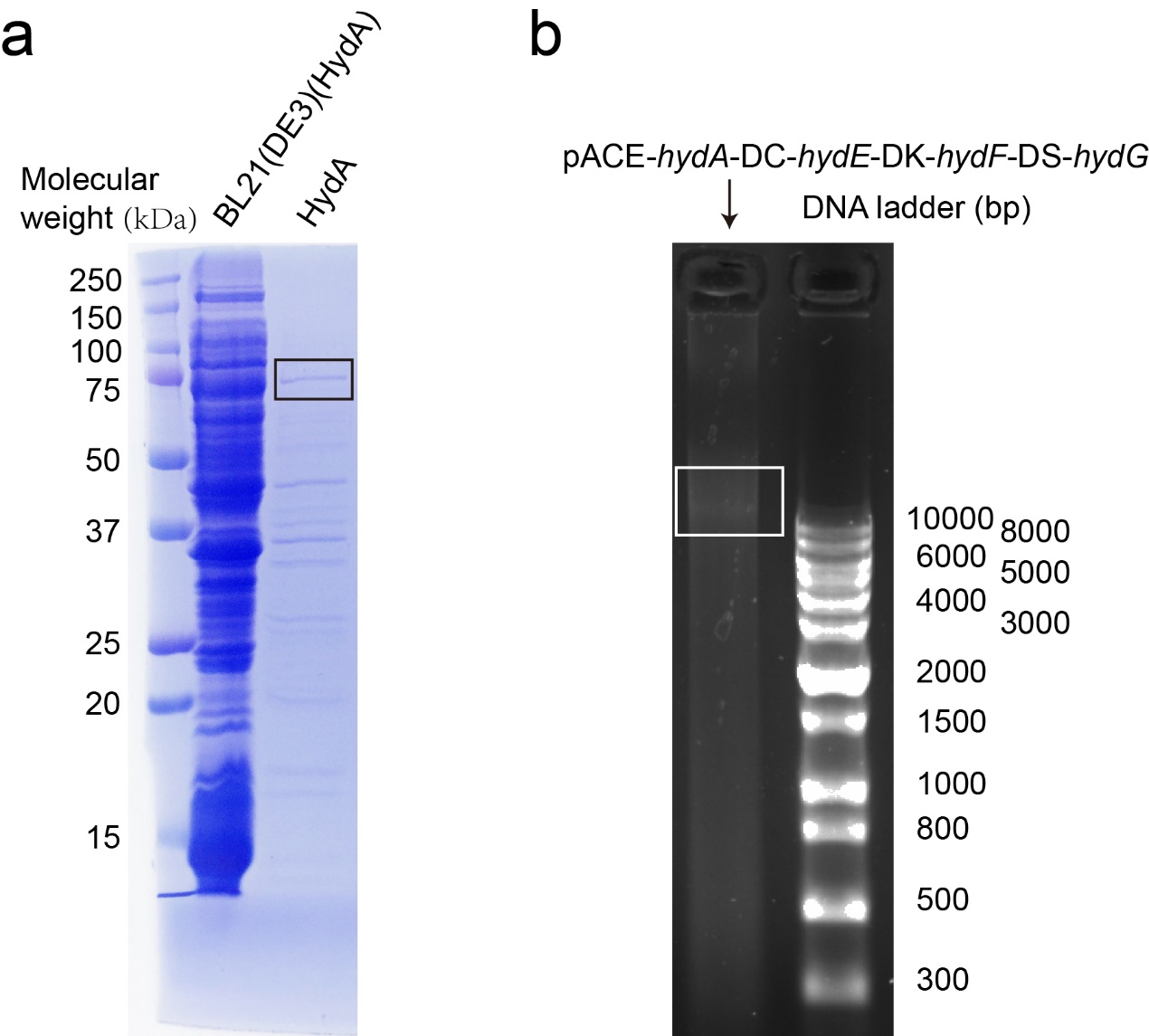


Supplementary Figure 25. Characterization of purified HydA protein and recombinant plasmid pACE-*hydA*-DC-*hydE*-DK-*hydF*-DS-*hydG*. (a) SDS-PAGE picture showed the successful expression of HydA protein. The molecular weight of HydA is 68.2 kDa. The band in rectangle box refers to purified HydA protein. (b) Agarose gel electrophoresis for the recombinant plasmid pACE-*hydA*-DC-*hydE*-DK-*hydF*-DS-*hydG*. The band in rectangle box refers to the fusion plasmid.


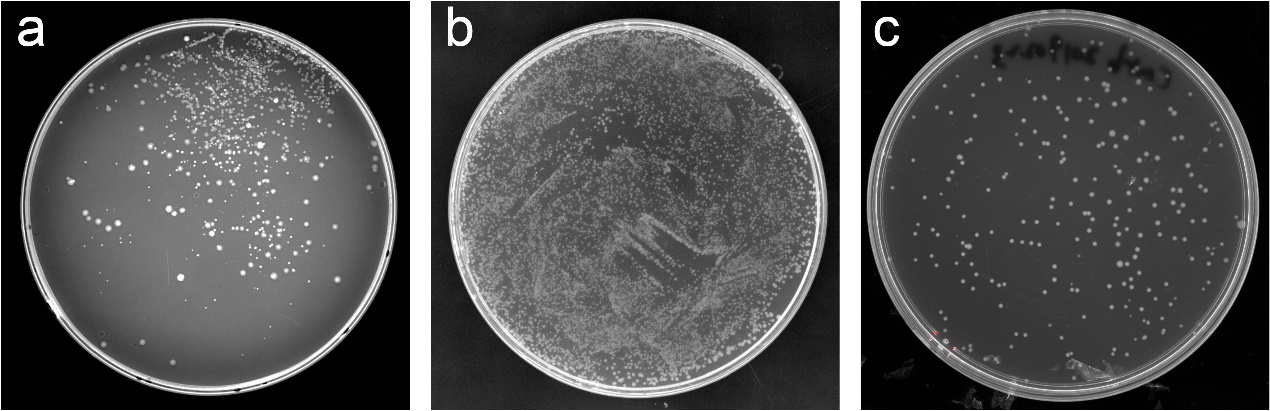


**Supplementary Figure 26. The regenerative properties of biofilm-based catalytic systems.** (a) Digital image of bacterial regrowth on agar plate for Tc_Receiver_/CsgA_His_ after PNP reduction. (b) Digital image of bacterial regrowth on agar plate for Tc_Receiver_/CsgA_His_ after photo-degradation of CR. (c) Digital image of bacterial regrowth on agar plate for BL21(DE3)/pAEFG after 12 h of photocatalytic hydrogen production. The solid agar plates were imaged using a ChemiDoc MP Imaging System (Bio-RAD). In a-b, the solid plate was supplemented with 34 μg/mL chloramphenicol. In the third reaction system for H_2_ production, the solid plate contained 50 μg/mL carbenicillin.


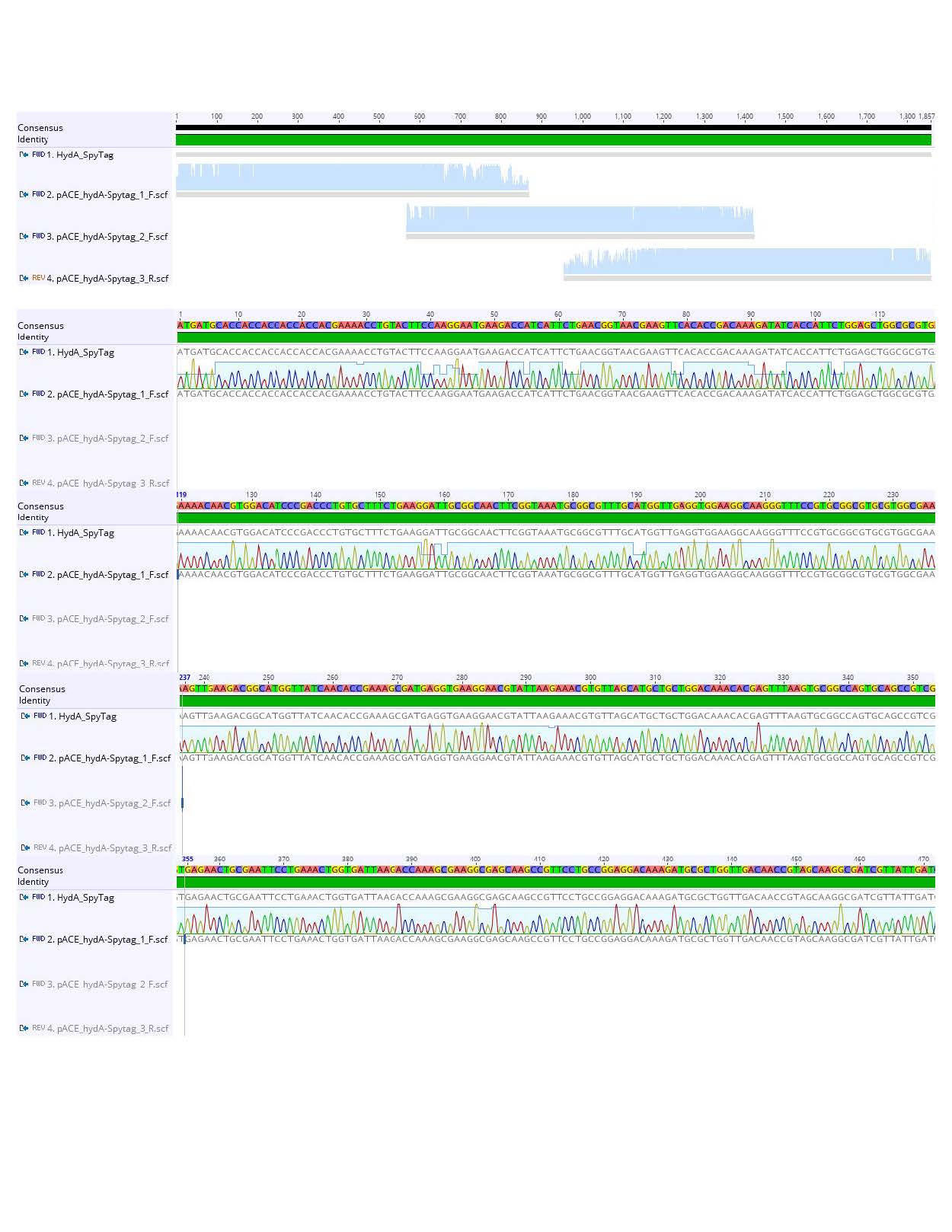


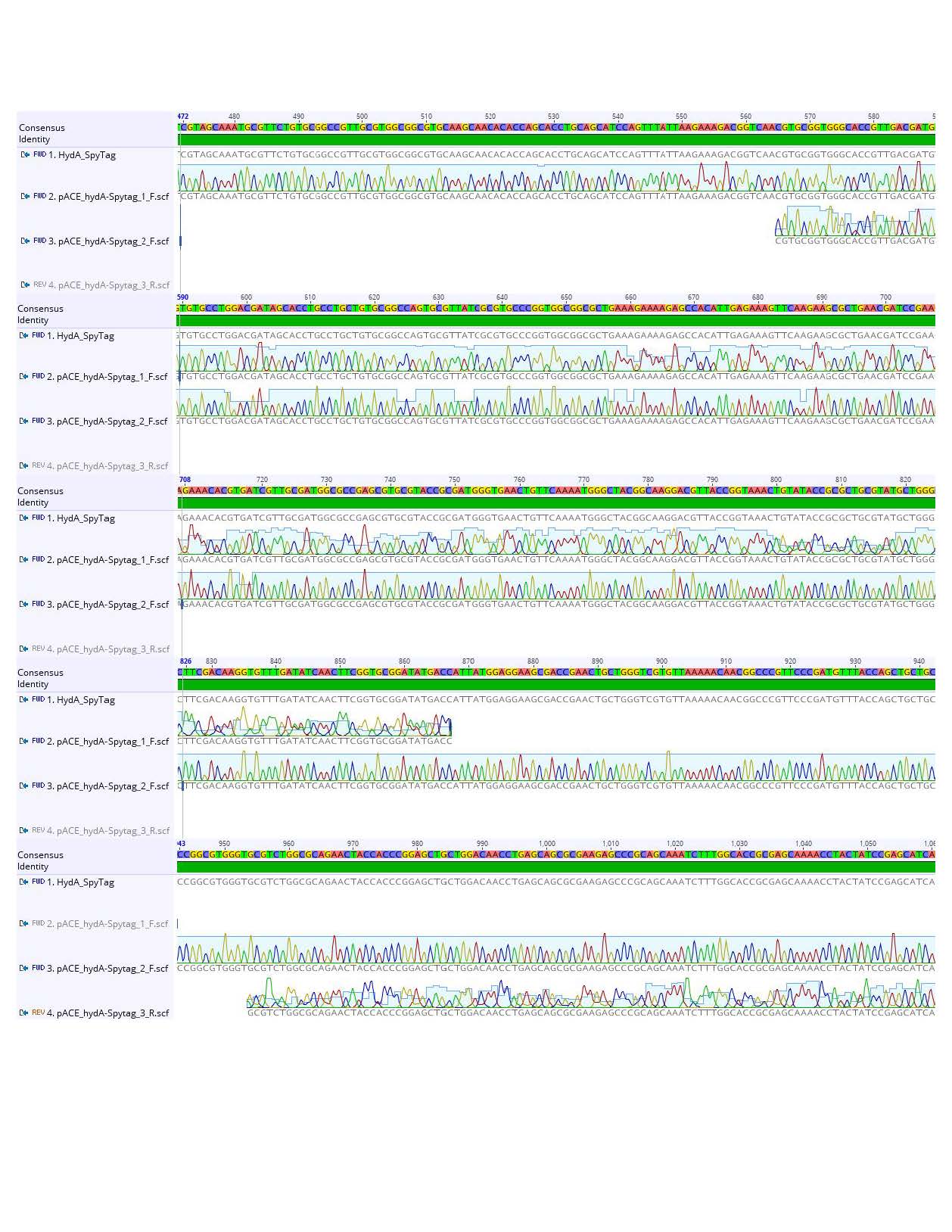

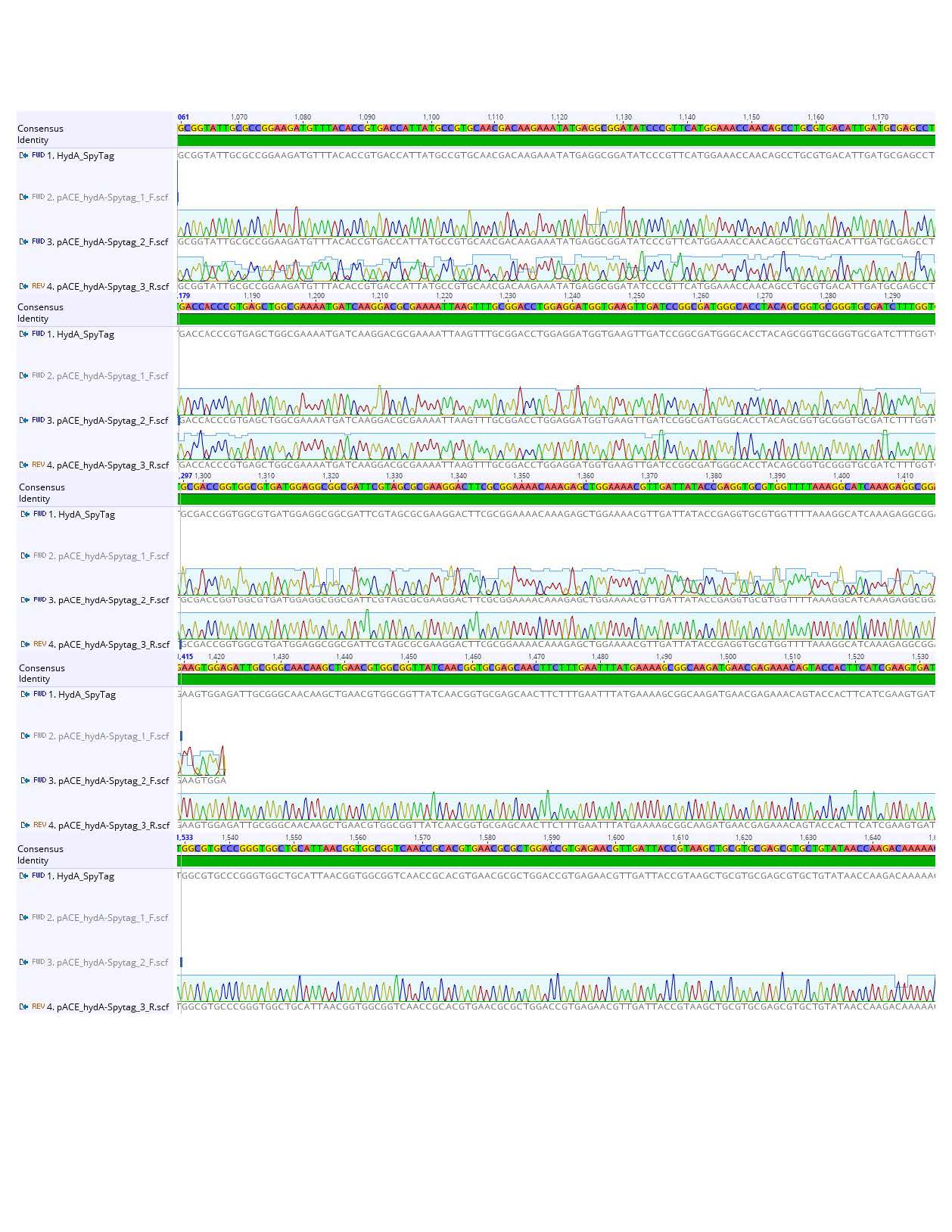

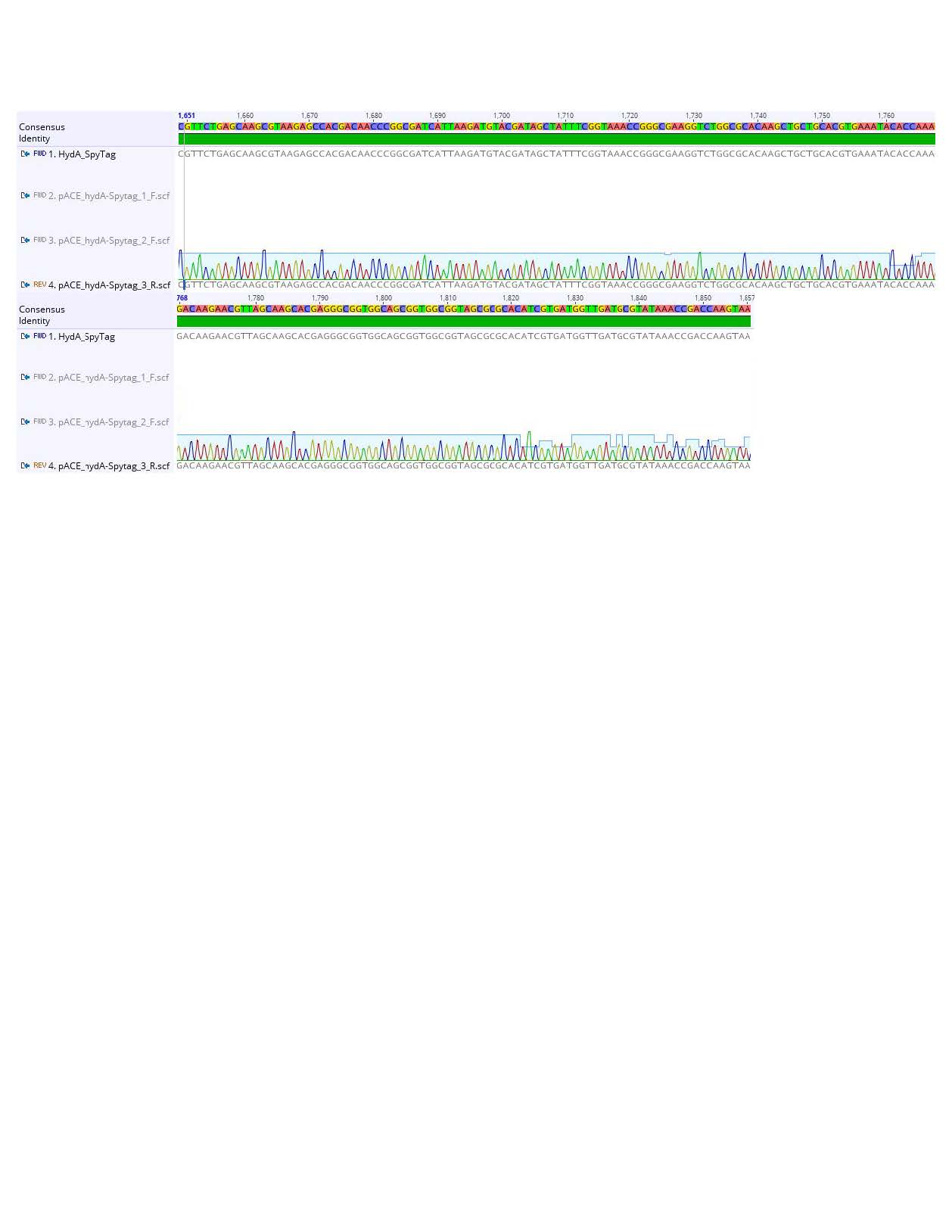


Supplementary Figure 27. Sequencing results for pACE-*hydA*.


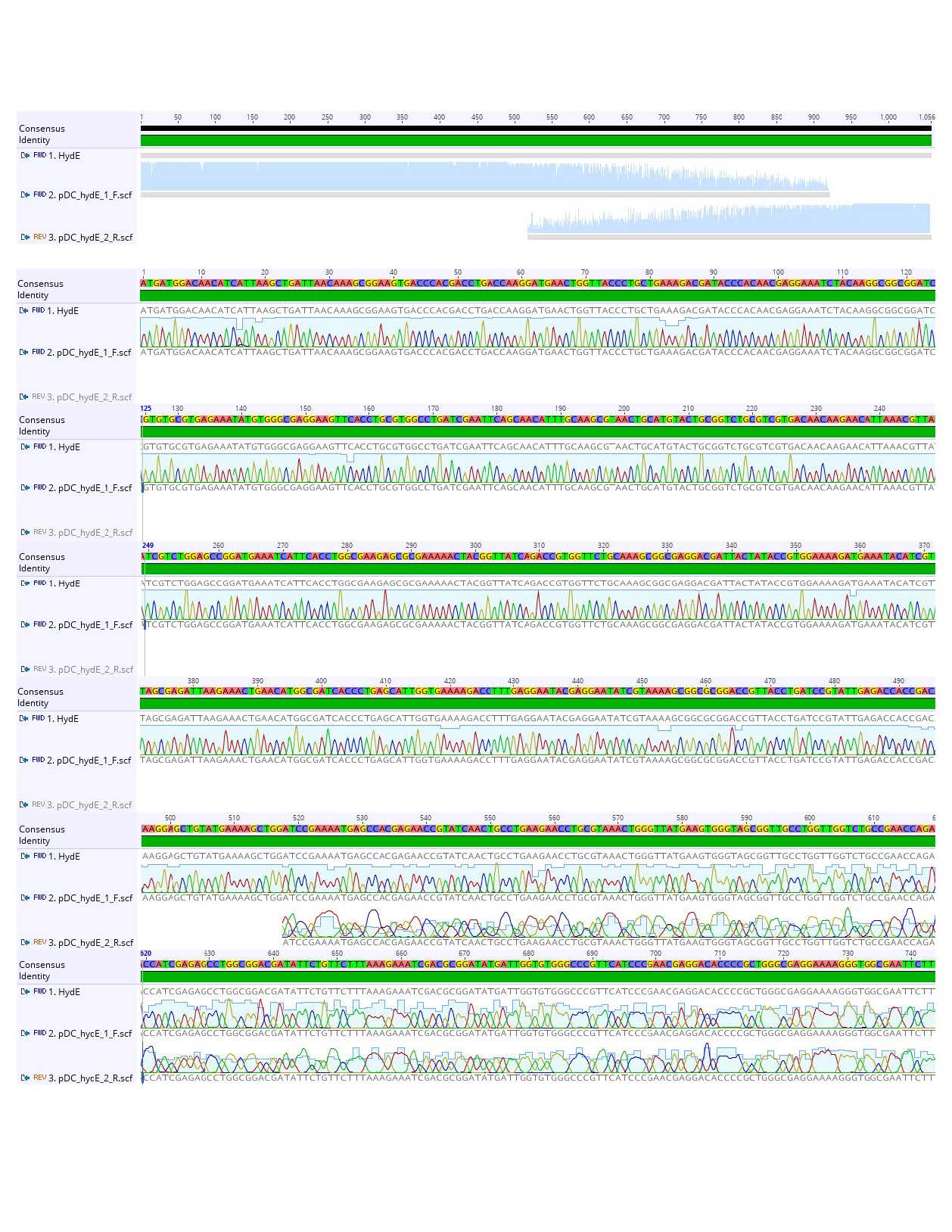

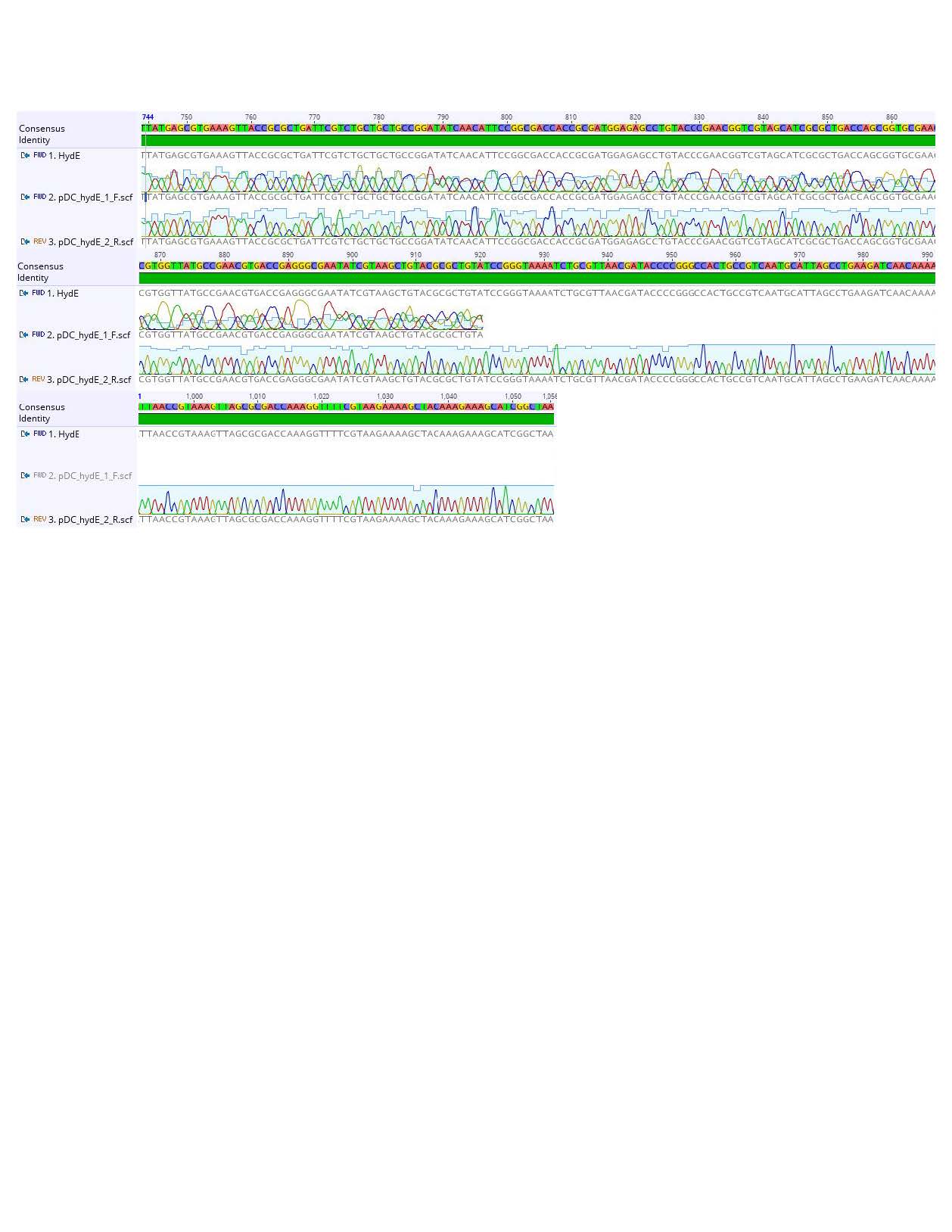


Supplementary Figure 28. Sequencing results of pDC-*hydE*.


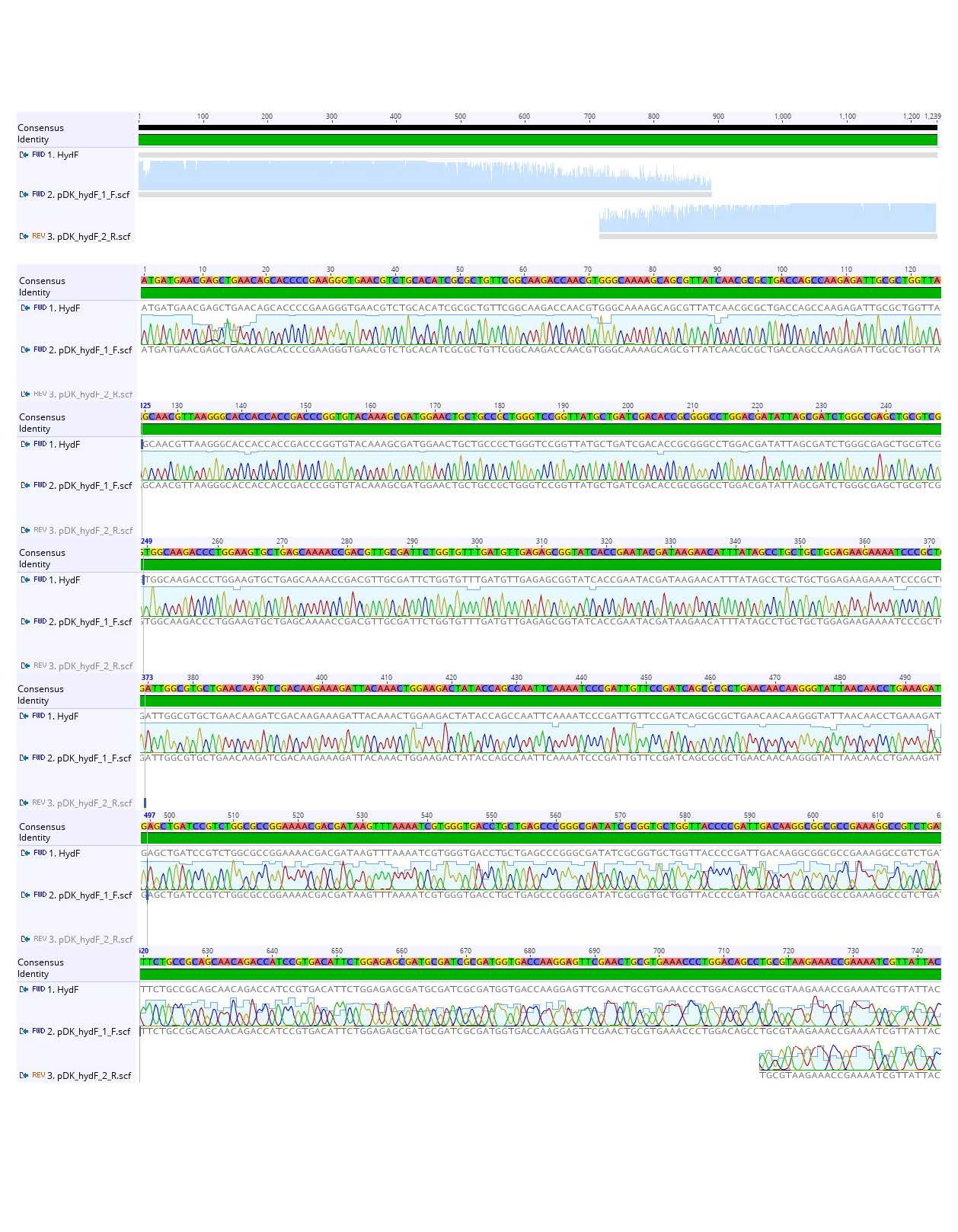

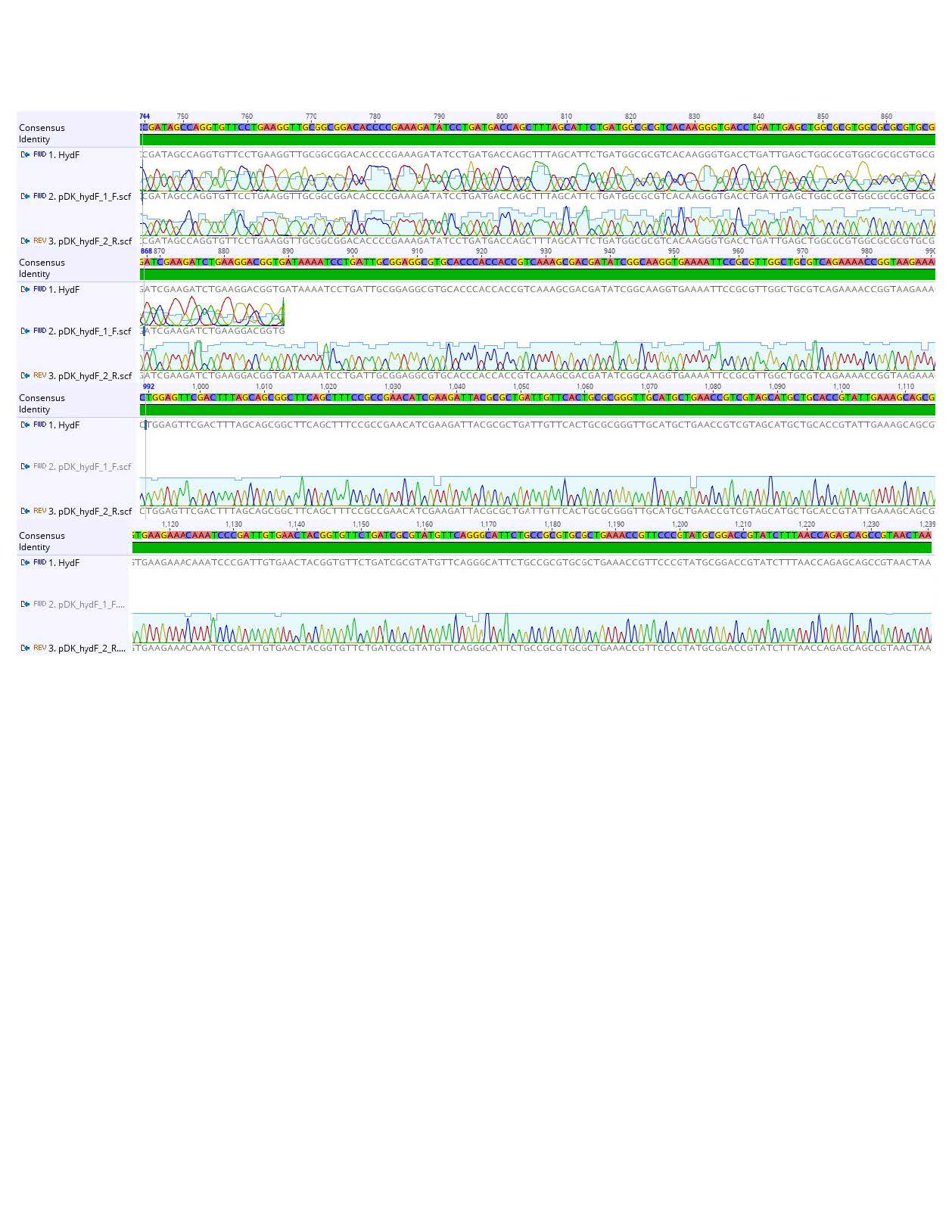


Supplementary Figure 29. Sequencing results of pDK-*hydF*.


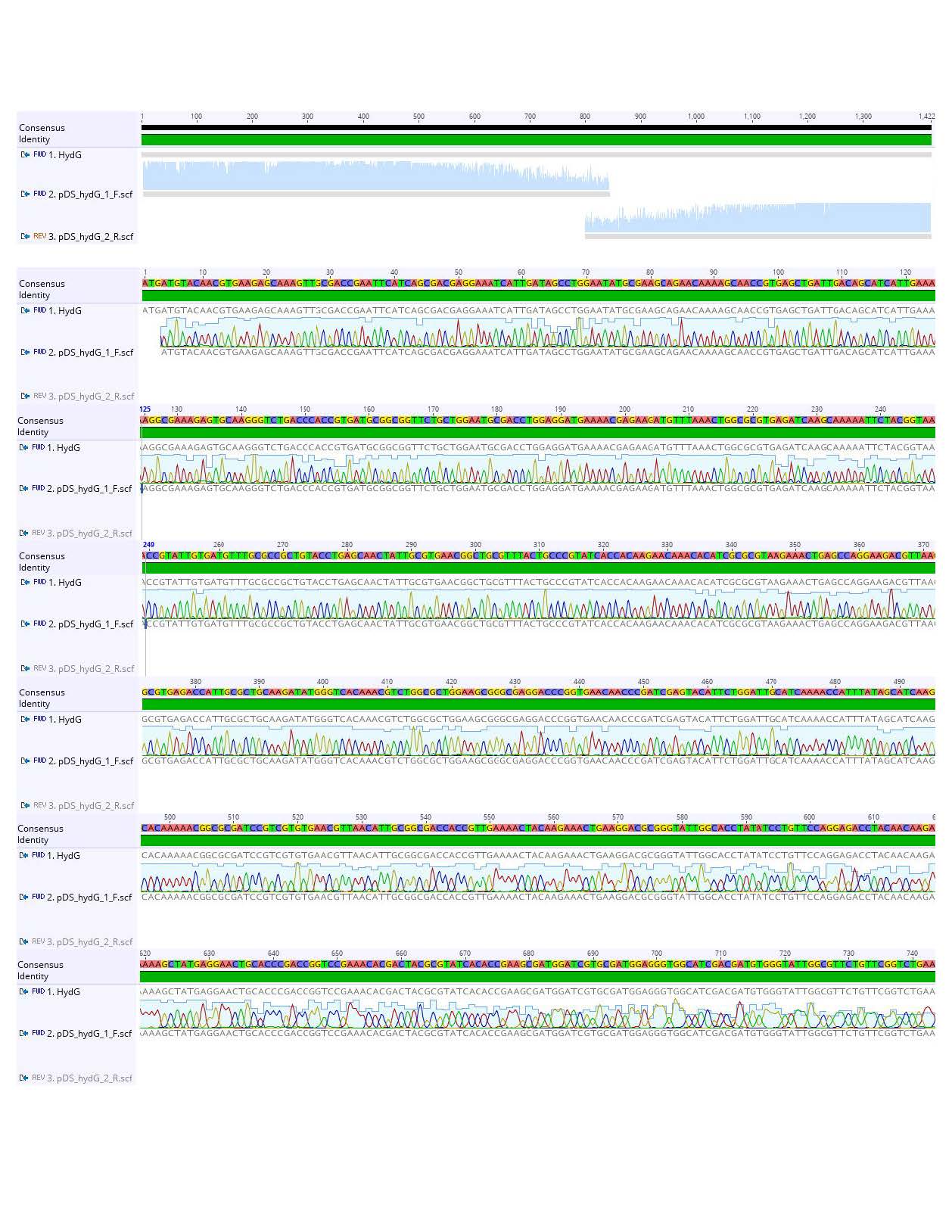

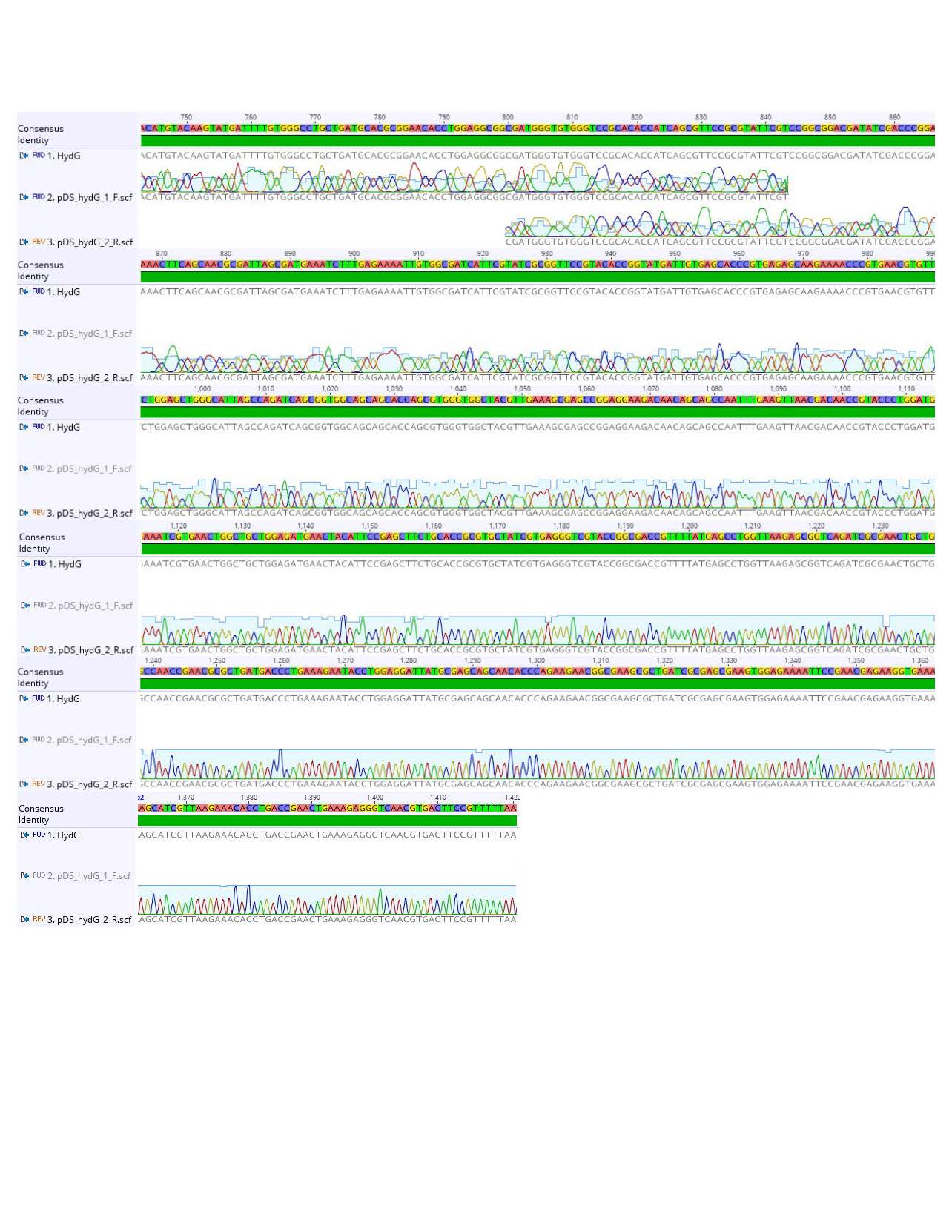


Supplementary Figure 30. Sequencing results of pDS-*hydG*.

**Supplementary Table 1. Synthetic primers and gene parts used in this work.**

| Name | Type | Sequence | Source |
| --- | --- | --- | --- |
| HydA-primer-F-with NdeI | HydA gene clone forward primer with Histag | GGAATTCCATATGATGCACCACCACCACCACCACGA | This work |
| HydA-primer-R-with XhoI | HydA gene clone reverse primer | CCGCTCGAGTTACTTGGTCGGTTTATACGCAT | This work |
| HydE-primer-F-with NdeI | HydE gene clone forward primer | GGAATTCCATATGATGGACAACATCATTAAGCTGATTAACAAAGCG | This work |
| HydE-primer-R-with XhoI | HydE gene clone reverse primer | CCGCTCGAGTTAGCCGATGCTTTCTTTG | This work |
| HydF-primer-F-with NdeI | HydF gene clone forward primer | GGAATTCCATATGATGAACGAGCTGAACAGCACCC | This work |
| HydF-primer-R-with XhoI | HydF gene clone reverse primer | CCGCTCGAGTTAGTTACGGCTGCTCT | This work |
| HydG-primer-F | HydG gene clone forward primer | GGAGATATACATATGAGGCCATGTACAACGTGAAGAGCAA | This work |
| HydG-primer-R | HydG gene clone reverse primer | TAAACGGAACTAGTCTCGAGTTAAAAACGGAAGTCACGTTG | This work |
| pDS -primer-F | pDS backbone clone forward primer | GGCCTCATATGTATATCTCC | This work |
| pDS -primer-R | pDS backbone clone reverse primer | CTCGAGACTAGTTCCGTTTA | This work |
| HydA-Ace-A-F | sequencing primer for middle sequence of HydA | GTGCAAGCAACACACCAG | This work |
| HydA | Synthesis by GenScript with optimal codon | ATGATGCACCACCACCACCACCACGAAAACCTGTACTTCCAAGGAATGAAGACCATCATTCTGAACGGTAACGAAGTTCACACCGACAAAGATATCACCATTCTGGAGCTGGCGCGTGAAAACAACGTGGACATCCCGACCCTGTGCTTTCTGAAGGATTGCGGCAACTTCGGTAAATGCGGCGTTTGCATGGTTGAGGTGGAAGGCAAGGGTTTCCGTGCGGCGTGCGTGGCGAAAGTTGAAGACGGCATGGTTATCAACACCGAAAGCGATGAGGTGAAGGAACGTATTAAGAAACGTGTTAGCATGCTGCTGGACAAACACGAGTTTAAGTGCGGCCAGTGCAGCCGTCGTGAGAACTGCGAATTCCTGAAACTGGTGATTAAGACCAAAGCGAAGGCGAGCAAGCCGTTCCTGCCGGAGGACAAAGATGCGCTGGTTGACAACCGTAGCAAGGCGATCGTTATTGATCGTAGCAAATGCGTTCTGTGCGGCCGTTGCGTGGCGGCGTGCAAGCAACACACCAGCACCTGCAGCATCCAGTTTATTAAGAAAGACGGTCAACGTGCGGTGGGCACCGTTGACGATGTGTGCCTGGACGATAGCACCTGCCTGCTGTGCGGCCAGTGCGTTATCGCGTGCCCGGTGGCGGCGCTGAAAGAAAAGAGCCACATTGAGAAAGTTCAAGAAGCGCTGAACGATCCGAAGAAACACGTGATCGTTGCGATGGCGCCGAGCGTGCGTACCGCGATGGGTGAACTGTTCAAAATGGGCTACGGCAAGGACGTTACCGGTAAACTGTATACCGCGCTGCGTATGCTGGGCTTCGACAAGGTGTTTGATATCAACTTCGGTGCGGATATGACCATTATGGAGGAAGCGACCGAACTGCTGGGTCGTGTTAAAAACAACGGCCCGTTCCCGATGTTTACCAGCTGCTGCCCGGCGTGGGTGCGTCTGGCGCAGAACTACCACCCGGAGCTGCTGGACAACCTGAGCAGCGCGAAGAGCCCGCAGCAAATCTTTGGCACCGCGAGCAAAACCTACTATCCGAGCATCAGCGGTATTGCGCCGGAAGATGTTTACACCGTGACCATTATGCCGTGCAACGACAAGAAATATGAGGCGGATATCCCGTTCATGGAAACCAACAGCCTGCGTGACATTGATGCGAGCCTGACCACCCGTGAGCTGGCGAAAATGATCAAGGACGCGAAAATTAAGTTTGCGGACCTGGAGGATGGTGAAGTTGATCCGGCGATGGGCACCTACAGCGGTGCGGGTGCGATCTTTGGTGCGACCGGTGGCGTGATGGAGGCGGCGATTCGTAGCGCGAAGGACTTCGCGGAAAACAAAGAGCTGGAAAACGTTGATTATACCGAGGTGCGTGGTTTTAAAGGCATCAAAGAGGCGGAAGTGGAGATTGCGGGCAACAAGCTGAACGTGGCGGTTATCAACGGTGCGAGCAACTTCTTTGAATTTATGAAAAGCGGCAAGATGAACGAGAAACAGTACCACTTCATCGAAGTGATGGCGTGCCCGGGTGGCTGCATTAACGGTGGCGGTCAACCGCACGTGAACGCGCTGGACCGTGAGAACGTTGATTACCGTAAGCTGCGTGCGAGCGTGCTGTATAACCAAGACAAAAACGTTCTGAGCAAGCGTAAGAGCCACGACAACCCGGCGATCATTAAGATGTACGATAGCTATTTCGGTAAACCGGGCGAAGGTCTGGCGCACAAGCTGCTGCACGTGAAATACACCAAAGACAAGAACGTTAGCAAGCACGAGGGCGGTGGCAGCGGTGGCGGTAGCGCGCACATCGTGATGGTTGATGCGTATAAACCGACCAAGTAA | This work |
| HydE | Synthesis by GenScript with optimal codon | ATGGACAACATCATTAAGCTGATTAACAAAGCGGAAGTGACCCACGACCTGACCAAGGATGAACTGGTTACCCTGCTGAAAGACGATACCCACAACGAGGAAATCTACAAGGCGGCGGATCGTGTGCGTGAGAAATATGTGGGCGAGGAAGTTCACCTGCGTGGCCTGATCGAATTCAGCAACATTTGCAAGCGTAACTGCATGTACTGCGGTCTGCGTCGTGACAACAAGAACATTAAACGTTATCGTCTGGAGCCGGATGAAATCATTCACCTGGCGAAGAGCGCGAAAAACTACGGTTATCAGACCGTGGTTCTGCAAAGCGGCGAGGACGATTACTATACCGTGGAAAAGATGAAATACATCGTTAGCGAGATTAAGAAACTGAACATGGCGATCACCCTGAGCATTGGTGAAAAGACCTTTGAGGAATACGAGGAATATCGTAAAAGCGGCGCGGACCGTTACCTGATCCGTATTGAGACCACCGACAAGGAGCTGTATGAAAAGCTGGATCCGAAAATGAGCCACGAGAACCGTATCAACTGCCTGAAGAACCTGCGTAAACTGGGTTATGAAGTGGGTAGCGGTTGCCTGGTTGGTCTGCCGAACCAGACCATCGAGAGCCTGGCGGACGATATTCTGTTCTTTAAAGAAATCGACGCGGATATGATTGGTGTGGGCCCGTTCATCCCGAACGAGGACACCCCGCTGGGCGAGGAAAAGGGTGGCGAATTCTTTATGAGCGTGAAAGTTACCGCGCTGATTCGTCTGCTGCTGCCGGATATCAACATTCCGGCGACCACCGCGATGGAGAGCCTGTACCCGAACGGTCGTAGCATCGCGCTGACCAGCGGTGCGAACGTGGTTATGCCGAACGTGACCGAGGGCGAATATCGTAAGCTGTACGCGCTGTATCCGGGTAAAATCTGCGTTAACGATACCCCGGGCCACTGCCGTCAATGCATTAGCCTGAAGATCAACAAAATTAACCGTAAAGTTAGCGCGACCAAAGGTTTTCGTAAGAAAAGCTACAAAGAAAGCATCGGCTAA | This work |
| HydF | Synthesis by GenScript with optimal codon | ATGAACGAGCTGAACAGCACCCCGAAGGGTGAACGTCTGCACATCGCGCTGTTCGGCAAGACCAACGTGGGCAAAAGCAGCGTTATCAACGCGCTGACCAGCCAAGAGATTGCGCTGGTTAGCAACGTTAAGGGCACCACCACCGACCCGGTGTACAAAGCGATGGAACTGCTGCCGCTGGGTCCGGTTATGCTGATCGACACCGCGGGCCTGGACGATATTAGCGATCTGGGCGAGCTGCGTCGTGGCAAGACCCTGGAAGTGCTGAGCAAAACCGACGTTGCGATTCTGGTGTTTGATGTTGAGAGCGGTATCACCGAATACGATAAGAACATTTATAGCCTGCTGCTGGAGAAGAAAATCCCGCTGATTGGCGTGCTGAACAAGATCGACAAGAAAGATTACAAACTGGAAGACTATACCAGCCAATTCAAAATCCCGATTGTTCCGATCAGCGCGCTGAACAACAAGGGTATTAACAACCTGAAAGATGAGCTGATCCGTCTGGCGCCGGAAAACGACGATAAGTTTAAAATCGTGGGTGACCTGCTGAGCCCGGGCGATATCGCGGTGCTGGTTACCCCGATTGACAAGGCGGCGCCGAAAGGCCGTCTGATTCTGCCGCAGCAACAGACCATCCGTGACATTCTGGAGAGCGATGCGATCGCGATGGTGACCAAGGAGTTCGAACTGCGTGAAACCCTGGACAGCCTGCGTAAGAAACCGAAAATCGTTATTACCGATAGCCAGGTGTTCCTGAAGGTTGCGGCGGACACCCCGAAAGATATCCTGATGACCAGCTTTAGCATTCTGATGGCGCGTCACAAGGGTGACCTGATTGAGCTGGCGCGTGGCGCGCGTGCGATCGAAGATCTGAAGGACGGTGATAAAATCCTGATTGCGGAGGCGTGCACCCACCACCGTCAAAGCGACGATATCGGCAAGGTGAAAATTCCGCGTTGGCTGCGTCAGAAAACCGGTAAGAAACTGGAGTTCGACTTTAGCAGCGGCTTCAGCTTTCCGCCGAACATCGAAGATTACGCGCTGATTGTTCACTGCGCGGGTTGCATGCTGAACCGTCGTAGCATGCTGCACCGTATTGAAAGCAGCGTGAAGAAACAAATCCCGATTGTGAACTACGGTGTTCTGATCGCGTATGTTCAGGGCATTCTGCCGCGTGCGCTGAAACCGTTCCCGTATGCGGACCGTATCTTTAACCAGAGCAGCCGTAACTAA | This work |
| HydG | Synthesis by GenScript with optimal codon | ATGTACAACGTGAAGAGCAAAGTTGCGACCGAATTCATCAGCGACGAGGAAATCATTGATAGCCTGGAATATGCGAAGCAGAACAAAAGCAACCGTGAGCTGATTGACAGCATCATTGAAAAGGCGAAAGAGTGCAAGGGTCTGACCCACCGTGATGCGGCGGTTCTGCTGGAATGCGACCTGGAGGATGAAAACGAGAAGATGTTTAAACTGGCGCGTGAGATCAAGCAAAAATTCTACGGTAACCGTATTGTGATGTTTGCGCCGCTGTACCTGAGCAACTATTGCGTGAACGGCTGCGTTTACTGCCCGTATCACCACAAGAACAAACACATCGCGCGTAAGAAACTGAGCCAGGAAGACGTTAAGCGTGAGACCATTGCGCTGCAAGATATGGGTCACAAACGTCTGGCGCTGGAAGCGGGCGAGGACCCGGTGAACAACCCGATCGAGTACATTCTGGATTGCATCAAAACCATTTATAGCATCAAGCACAAAAACGGCGCGATCCGTCGTGTGAACGTTAACATTGCGGCGACCACCGTTGAAAACTACAAGAAACTGAAGGACGCGGGTATTGGCACCTATATCCTGTTCCAGGAGACCTACAACAAGAAAAGCTATGAGGAACTGCACCCGACCGGTCCGAAACACGACTACGCGTATCACACCGAAGCGATGGATCGTGCGATGGAGGGTGGCATCGACGATGTGGGTATTGGCGTTCTGTTCGGTCTGAACATGTACAAGTATGATTTTGTGGGCCTGCTGATGCACGCGGAACACCTGGAGGCGGCGATGGGTGTGGGTCCGCACACCATCAGCGTTCCGCGTATTCGTCCGGCGGACGATATCGACCCGGAAAACTTCAGCAACGCGATTAGCGATGAAATCTTTGAGAAAATTGTGGCGATCATTCGTATCGCGGTTCCGTACACCGGTATGATTGTGAGCACCCGTGAGAGCAAGAAAACCCGTGAACGTGTTCTGGAGCTGGGCATTAGCCAGATCAGCGGTGGCAGCAGCACCAGCGTGGGTGGCTACGTTGAAAGCGAGCCGGAGGAAGACAACAGCAGCCAATTTGAAGTTAACGACAACCGTACCCTGGATGAAATCGTGAACTGGCTGCTGGAGATGAACTACATTCCGAGCTTCTGCACCGCGTGCTATCGTGAGGGTCGTACCGGCGACCGTTTTATGAGCCTGGTTAAGAGCGGTCAGATCGCGAACTGCTGCCAACCGAACGCGCTGATGACCCTGAAAGAATACCTGGAGGATTATGCGAGCAGCAACACCCAGAAGAACGGCGAAGCGCTGATCGCGAGCGAAGTGGAGAAAATTCCGAACGAGAAGGTGAAAAGCATCGTTAAGAAACACCTGACCGAACTGAAAGAGGGTCAACGTGACTTCCGTTTTTAA | This work |
| CsgA_His_ | Curli major component genes | ATGAAACTTTTAAAAGTAGCAGCAATTGCAGCAATC  GTATTCTCCGGTAGCGCTCTGGCAGGTGTTGTTCCTC  AGTACGGCGGCGGCGGTAACCACGGTGGTGGCGGTAATAATAGCGGCCCAAATCACCATCACCATCACCACC  ATTCTGAGCTGAACATTTACCAGTACGGTGGCGGTA  ACTCTGCACTTGCTCTGCAAACTGATGCCCGTAACTC  TGACTTGACTATTACCCAGCATGGCGGCGGTAATGG  TGCAGATGTTGGTCAGGGCTCAGATGACAGCTCAAT  CGATCTGACCCAACGTGGCTTCGGTAACAGCGCTAC  TCTTGATCAGTGGAACGGCAAAAATTCTGAAATGAC  GGTTAAACAGTTCGGTGGTGGCAACGGTGCTGCAGT  TGACCAGACTGCATCTAACTCCTCCGTCAACGTGACT  CAGGTTGGCTTTGGTAACAACGCGACCGCTCATCAG  TACCACCATCACCATCACCACCATTAA | [8] |

Supplementary Table 2. Plasmids used in this work.

| Plasmid name | Plasmid ID | Description | Source |
| --- | --- | --- | --- |
| pZA-CmR-rr12-pL(tetO)-  *csgA_His_* | pAYC003 | p15A origin, Cm resistance, rr12 riboregulator, pL(tetO) promoter, CsgA_His_ output gene | [8] |
| pACE-*hydA* | pIMBE001 | Acceptor plasmid | This work |
| pDC-*hydE* | pIBME002 | Donor plasmid | This work |
| pDK-*hydF* | pIBME003 | Donor plasmid | This work |
| pDS-*hydG* | pIBME004 | Donor plasmid | This work |
| pACE-*hydA-*DC-*hydE-*DK-*hydF-*DS-*hydG* | pIBME005 | Resulting expression plasmid for HydA, HydE, HydF and HydG. | This work |

Supplementary Table 3. Strains used in this work.

| Strain name | Strain ID | Description | Antibiotic resistance | Source |
| --- | --- | --- | --- | --- |
| MG1655 PRO ∆*CsgA*  *ompR234*  *Referred to as “*∆*CsgA*  *ompR234” in figures* | fAYC002 | E. coli strain with constitutive high level expression of *tetR* and *lacI* from PRO cassette derived from pZS4Int-lacI/tetR, with *CsgA* knocked out, and with *ompR234* mutation that confers ability to produce curli fibrils in liquid M63 minimal media. | Spec, Kan | [8] |
| Tc_Receiver_/CsgA_His_ | fAYC003 | *E. coli* strain that expresses CsgA_His_ under tight regulation by a tetracycline (Tc) inducer-responsive riboregulator. Made by transforming pZA-CmR-rr12-  pL(tetO)-CsgA_His_ plasmid into MG1655 PRO ∆CsgA ompR234. | Spec, Kan,  Cm | [8] |
| BL21(DE3)(HydA) | fLY001 | *E. coli* strain that expresses HydA, HydE, HydF and HydG by a T7 promoter. | Amp, Kan, Cm, Spec | This work |

Supplementary Table 4. Protein sequences in this work.

| Protein name | Protein sequence | Source |
| --- | --- | --- |
| CsgA-His | 1 MKLLKVAAIA AIVFSGSALA  21 GVVPQYGGGG NHGGGGNNSG  41 PNHHHHHHHS ELNIYQYGGG  61 NSALALQTDA RNSDLTITQH  81 GGGNGADVGQ GSDDSSIDLT  101 QRGFGNSATL DQWNGKNSEM  121 TVKQFGGGNG AAVDQTASNS  141 SVNVTQVGFG NNATAHQYHH  161 HHHHH* | [8] |
| HydA | 1 MMHHHHHHEN LYFQGMKTII  21 LNGNEVHTDK DITILELARE  41 NNVDIPTLCF LKDCGNFGKC  61 GVCMVEVEGK GFRAACVAKV  81 EDGMVINTES DEVKERIKKR  101 VSMLLDKHEF KCGQCSRREN  121 CEFLKLVIKT KAKASKPFLP  141 EDKDALVDNR SKAIVIDRSK  161 CVLCGRCVAA CKQHTSTCSI  181 QFIKKDGQRA VGTVDDVCLD  201 DSTCLLCGQC VIACPVAALK  221 EKSHIEKVQE ALNDPKKHVI  241 VAMAPSVRTA MGELFKMGYG  261 KDVTGKLYTA LRMLGFDKVF  281 DINFGADMTI MEEATELLGR  301 VKNNGPFPMF TSCCPAWVRL  321 AQNYHPELLD NLSSAKSPQQ  341 IFGTASKTYY PSISGIAPED  361 VYTVTIMPCN DKKYEADIPF  381 METNSLRDID ASLTTRELAK  401 MIKDAKIKFA DLEDGEVDPA  421 MGTYSGAGAI FGATGGVMEA  441 AIRSAKDFAE NKELENVDYT  461 EVRGFKGIKE AEVEIAGNKL  481 NVAVINGASN FFEFMKSGKM  501 NEKQYHFIEV MACPGGCING  521 GGQPHVNALD RENVDYRKLR  541 ASVLYNQDKN VLSKRKSHDN  561 PAIIKMYDSY FGKPGEGLAH  581 KLLHVKYTKD KNVSKHEGGG  601 SGGGSAHIVM VDAYKPTK* | This work |
| HydE | 1 MDNIIKLINK AEVTHDLTKD  21 ELVTLLKDDT HNEEIYKAAD  41 RVREKYVGEE VHLRGLIEFS  61 NICKRNCMYC GLRRDNKNIK  81 RYRLEPDEII HLAKSAKNYG  101 YQTVVLQSGE DDYYTVEKMK  121 YIVSEIKKLN MAITLSIGEK  141 TFEEYEEYRK SGADRYLIRI  161 ETTDKELYEK LDPKMSHENR  181 INCLKNLRKL GYEVGSGCLV  201 GLPNQTIESL ADDILFFKEI  221 DADMIGVGPF IPNEDTPLGE  241 EKGGEFFMSV KVTALIRLLL  261 PDINIPATTA MESLYPNGRS  281 IALTSGANVV MPNVTEGEYR  301 KLYALYPGKI CVNDTPGHCR  321 QCISLKINKI NRKVSATKGF  341 RKKSYKESIG * | This work |
| HydF | 1 MNELNSTPKG ERLHIALFGK  21 TNVGKSSVIN ALTSQEIALV  41 SNVKGTTTDP VYKAMELLPL  61 GPVMLIDTAG LDDISDLGEL  81 RRGKTLEVLS KTDVAILVFD  101 VESGITEYDK NIYSLLLEKK  121 IPLIGVLNKI DKKDYKLEDY  141 TSQFKIPIVP ISALNNKGIN  161 NLKDELIRLA PENDDKFKIV  181 GDLLSPGDIA VLVTPIDKAA  201 PKGRLILPQQ QTIRDILESD  221 AIAMVTKEFE LRETLDSLRK  241 KPKIVITDSQ VFLKVAADTP  261 KDILMTSFSI LMARHKGDLI  281 ELARGARAIE DLKDGDKILI  301 AEACTHHRQS DDIGKVKIPR  321 WLRQKTGKKL EFDFSSGFSF  341 PPNIEDYALI VHCAGCMLNR  361 RSMLHRIESS VKKQIPIVNY  381 GVLIAYVQGI LPRALKPFPY  401 ADRIFNQSSR N* | This work |
| HydG | 1 MYNVKSKVAT EFISDEEIID  21 SLEYAKQNKS NRELIDSIIE  41 KAKECKGLTH RDAAVLLECD  61 LEDENEKMFK LAREIKQKFY  81 GNRIVMFAPL YLSNYCVNGC  101 VYCPYHHKNK HIARKKLSQE  121 DVKRETIALQ DMGHKRLALE  141 AGEDPVNNPI EYILDCIKTI  161 YSIKHKNGAI RRVNVNIAAT  181 TVENYKKLKD AGIGTYILFQ  201 ETYNKKSYEE LHPTGPKHDY  221 AYHTEAMDRA MEGGIDDVGI  241 GVLFGLNMYK YDFVGLLMHA  261 EHLEAAMGVG PHTISVPRIR  281 PADDIDPENF SNAISDEIFE  301 KIVAIIRIAV PYTGMIVSTR  321 ESKKTRERVL ELGISQISGG  341 SSTSVGGYVE SEPEEDNSSQ  361 FEVNDNRTLD EIVNWLLEMN  381 YIPSFCTACY REGRTGDRFM  401 SLVKSGQIAN CCQPNALMTL  421 KEYLEDYASS NTQKNGEALI  441 ASEVEKIPNE KVKSIVKKHL  461 TELKEGQRDF RF* | This work |

**References**

1. Du Roure O, Debiemme-Chouvy C, Malthete J *et al.* Functionalizing surfaces with nickel ions for the grafting of proteins. *Langmuir*. 2003; **19**(10): 4138-43.

2. Wang X, Pu J, An B *et al.* Programming Cells for Dynamic Assembly of Inorganic Nano-Objects with Spatiotemporal Control. *Adv Mater*. 2018; **30**(16): e1705968.

3. Wong OA, Heinecke CL, Simone AR *et al.* Ligand symmetry-equivalence on thiolate protected gold nanoclusters determined by NMR spectroscopy. *Nanoscale*. 2012; **4**(14): 4099-102.

4. Zhu W, Michalsky R, Metin O *et al.* Monodisperse Au nanoparticles for selective electrocatalytic reduction of CO2 to CO. *J Am Chem Soc*. 2013; **135**(45): 16833-6.

5. Zhong X, Feng Y, Knoll W *et al.* Alloyed Zn(x)Cd(1-x)S nanocrystals with highly narrow luminescence spectral width. *J Am Chem Soc*. 2003; **125**(44): 13559-63.

6. Chiu Y-H and Hsu Y-J. Au@Cu 7 S 4 yolk@shell nanocrystal-decorated TiO 2 nanowires as an all-day-active photocatalyst for environmental purification. *Nano Energy*. 2017; **31**: 286-95.

7. Kallberg M, Wang H, Wang S *et al.* Template-based protein structure modeling using the RaptorX web server. *Nat Protoc*. 2012; **7**(8): 1511-22.

8. Chen AY, Deng Z, Billings AN *et al.* Synthesis and patterning of tunable multiscale materials with engineered cells. *Nat Mater*. 2014; **13**(5): 515-23.
